# *Ikhnos*: A Novel Software to Register and Analyze Bone Surface Modifications Based on Three-Dimensional Documentation

**DOI:** 10.3390/ani12202861

**Published:** 2022-10-20

**Authors:** Rocío Mora, Julia Aramendi, Lloyd A. Courtenay, Diego González-Aguilera, José Yravedra, Miguel Ángel Maté-González, Diego Prieto-Herráez, José Mª Vázquez-Rodríguez, Isabel Barja

**Affiliations:** 1 Department of Cartographic and Land Engineering, Higher Polytechnic School of Avila, Universidad de Salamanca, Hornos Caleros 50, 05003 Ávila, Spain; 2 Department of Geology, Facultad de Ciencia y Tecnología, Universidad del País Vasco-Euskal Herriko Unibertsitatea (UPV/EHU), Barrio Sarriena s/n, 48940 Leioa, Spain; 3 Department of Prehistory, Ancient History and Archaeology, Universidad Complutense de Madrid, Prof. Aranguren 8 s/n, 28040 Madrid, Spain; 4 C.A.I. Archaeometry and Archaeological Analysis, Universidad Complutense de Madrid, 28040 Madrid, Spain; 5 Department of Topographic and Cartography Engineering, Higher Technical School of Engineers in Topography, Geodesy and Cartography, Universidad Politécnica de Madrid, Mercator 2, 28031 Madrid, Spain; 6 Institute of Fundamental Physics and Mathematics, Merced Building, Universidad de Salamanca, Plaza de la Merced 1, 37008 Salamanca, Spain; 7 Department of Prehistory and Archaeology, Humanities Faculty, UNED, C/Senda del Rey, 7, 28040 Madrid, Spain; 8 Zoology Unit, Department of Biology, Universidad Autónoma de Madrid, C/Darwin 2, Campus Universitario de Cantoblanco, 28049 Madrid, Spain; 9 Centre of Investigation in Biodiversity and Global Change (CIBC-UAM), Universidad Autónoma de Madrid, 28049 Madrid, Spain

**Keywords:** taphonomy, bone surface modifications, breakage patterns, 3D registration and analysis

## Abstract

**Simple Summary:**

Here we present a novel open-access 3D software called *Ikhnos* to register and analyze bone surface modifications in bone assemblages using a sample of wild and captive wolf populations as an example to demonstrate the possibilities offered by this newly developed toolkit. The study of bone surface modifications has been proven crucial in understanding archaeological and paleontological site formation processes, including the identification of the assemblage causal agent. The use of the tools provided by *Ikhnos* will help interpret the fossil record by allowing the generation of accurate biological and physicochemical referential models as well as the precise documentation of bone surface modifications in archaeological and paleontological assemblages.

**Abstract:**

The study of bone surface modifications (BSM) is crucial in understanding site formation processes and the identification of the causal agent behind bone assemblages in the fossil record. In that line, many efforts have been made to generate referential models based on feeding experiments and human butchery simulations that can then be used to interpret the patterns observed in archaeological and paleontological sites. Considering these needs, we developed a novel open-access three-dimensional (3D) software called *Ikhnos* for the study of BSM distribution patterns on limb long bones. This software is comprised of all the necessary tools for the 3D documentation of BSM and bone breakage patterns, as well as the subsequent statistical analysis of this data due to the integration of an exclusive R library, the *IkhnosToolBox*. Additionally, *Ikhnos* integrates tools for bone survivorship calculations that could facilitate the estimation of the minimum number of elements (MNE) and minimum number of individuals (MNI). As a demonstration of its precision, here we present a case study analyzing the modifications produced by wild and captive wolf (*Canis lupus signatus*) populations of the Iberian Peninsula on deer carcasses.

## 1. Introduction

In taphonomy, the study of bone surface modifications (BSMs) has proven extremely important to identify the causal agent behind bone assemblages in archaeological and paleontological records. Mark morphology (considering shape and size features), as well as the identification of specific microscopic features, has allowed for the differentiation between human, carnivore, and other biotic or abiotic agents, by means of direct approaches based on the use of hand lenses, microscopes, or molds [1,2,3,4,5,6,7,8,9,10,11]. Likewise, three-dimensional (3D) modeling has also proven to be a powerful tool for the digitization and analysis of BSMs [12,13,14,15,16,17]. Data in both two and three dimensions have allowed the application of cutting-edge techniques such as geometric morphometrics [18,19,20,21,22,23,24] and artificially intelligent (AI) algorithms [25,26,27,28,29], for the identification of different taphonomic agents. The evaluation of BSMs results is crucial to understanding site formation processes, as well as to evaluating possible bias that might affect site integrity and, subsequently, its interpretation.

Nevertheless, not only the analysis of BSM morphology has resulted to be relevant for agency determination. The study of carnivore and anthropogenic mark frequencies [30,31,32,33], as well as their location on specific bones such as vertebrae or ribs [34], and the anatomical distribution within skeletal elements, are key in the identification of potential agents and the order in which they access carcasses [35,36,37]. Each of these variables has thus resulted in being useful in the proposal of multiple models to determine agency. Existing models based on observations made on bone damage patterns created by modern carnivores or after human butchery activities are meant to help understand carnivore behavior as well as the human–carnivore interactions over the course of human evolution.

Regarding the anatomical distribution of BSMs, previous models have proposed the definition of the so-called “hot and cold zones” to assess primary, secondary, and marginal access to carcasses in assemblages recovered from areas subject to strong trophic competition [38]. To date, research considering BSM anatomical distribution has been restricted to bidimensional documentation, either based on the use of graphic templates of bones that enable the visual comparison of mark distribution patterns in different scenarios [38,39,40], or through a digitized geographical information system (GIS)-based approach that also allows for statistical comparison [41,42,43,44,45,46,47,48]. GIS-based approaches have also served the quantification of essential variables in taphonomic studies such as the minimum number of elements (MNE) and the minimum number of individuals (MNI) [45,49]. The application of such techniques has provided a graphical means of representing differences in BSM scattering patterns based on the modifying agent, and has significantly improved the quantitative assessment of the initial manual documentation of BSMs (Table 1), though not without generating certain concerns regarding methodological appropriateness [50], and accuracy throughout the documentation process [51]. Additionally, the recently developed TIPZOO software [52] provides a standardized solution for archaeological documentation and expands the statistical repertoire through its link to the open-access statistical software R and QGIS-based graphical output. However, despite its significant contribution, TIPZOO is not only limited to two-dimensional recording, but BSM registration is constrained to the selection of specific bone segments (e.g., proximal or distal epiphysis and diaphysis), providing a less precise way of documenting marks (Table 1). Despite being open-access, TIPZOO presents some accessibility problems as it only runs on FileMaker, a non-open-access software. 

Considering all the challenges involved in the study and documentation of BSM distributions, and the great potential that such analyses might offer for agency determination in fossil assemblages, a beta version of a novel open-access 3D software (*Ikhnos*) for the study of BSM distribution patterns on limb long bones has been successfully used for the documentation and analysis of experimental and archaeological samples [50,54,55]. In the present study, we introduce the official release of the *Ikhnos* software that comprises all the necessary tools for the 3D documentation and statistical analysis of BSMs and bone breakage patterns. Here we also demonstrate its utility, and statistical and graphic power, through a case study on the modifications made by wild and captive wolves on middle-size carcasses. More technical information about the software and its use can be found in the supplementary material (Supplementary Files S1 and S2).

Canids, in general, have not been the focus of attention in taphonomic and experimental studies (mostly based on African contexts), as opposed to felids or hyaenidae, though recently a wider range of studies on bone modification and prey consumption by wolf populations have been developed [45,56,57,58,59,60,61,62,63,64,65,66,67,68,69,70]. Nevertheless, canids constitute a significant part of the Pleistocene carnivore guild. The extent to which canids might have overlapped and competed with other carnivores, including humans, is not fully understood yet due to the lack of referential studies. Additionally, our understanding of canid bone damage is affected by the experimentation with animals in captivity, which has been said to generate more intense modification patterns, and thus does not provide an appropriate referential framework that could help identify the action of canids in the fossil record [57,60,65,71]. In the present study, we further assess this statement using the 3D documentation and statistical tools provided by *Ikhnos* in order to contribute to the development of proper frames of reference that might serve archaeological and paleontological interpretations.

## 2. Materials and Methods

### 2.1. Ikhnos and IkhnosToolBox

*Ikhnos* is a new 3D-geospatial software for taphonomic and zooarchaeological analysis (Supplementary File S1) that integrates all the necessary tools for bone survivorship documentation, and BSM registration and statistical analysis. *Ikhnos* is written in the C language (C++ 11), with a Graphical User Interface (GUI) implemented using the Qt framework, and employing MySQL as a relational database management system. The available statistical analyses were programmed in the R programming language (v.4.0, www.rproject.org (accessed on 15 June 2022)) [72], and compiled in the form of an R library called *IkhnosToolBox* [73]. *IkhnosToolBox* provides tools for the handling of 3D spatial and circular statistics, while tests for frequency analyses have also been included (Supplementary File S2). *Ikhnos* can incorporate additional data (i.e., metadata), including basic information about the site and the finding, as well as data that might be valuable to evaluate the assemblage integrity (e.g., fracture type). All the recorded information is stored in a local relational database, which also allows for future consultation, editing, and selection for statistical analyses.

3D models of all limb long bones (including the left and right sides) of a horse, deer and human are available and serve as template for the documentation of fragmentation patterns and BSM presence and location. Cervid bone models are meant to be used to register BSM anatomical distribution on all kinds of animals with the exception of equids and primates, regardless of the carcass size. However, a wider range of 3D models will be included in the future to provide more accurate templates for BSM documentation. BSM digitization requires the registration of some specifics regarding the site (e.g., name), the element (e.g., taxon, anatomical part, age, side, animal size), the item ID, and the specification of the mark type (e.g., cut mark, score, percussion, or tooth pit). Additional data on the site (e.g., country, coordinates), the item (e.g., coordinates, level, square), and observed breakage patterns (e.g., green or dry fracture, shaft circumference type according to Bunn [74]) can also be recorded. 

Bone survivorship can be registered in a visual way using the heatmaps function available in *Ikhnos*. Bone 3D models can be edited and segmented to represent the preserved bone portion in each case. Bone survivorship is documented per specimen through polygonal cropping directly on the 3D point cloud representing the selected long bone. Cropped 3D models can then be reloaded and superimposed by selecting specific conditions (i.e., left femora of size 3 individuals of a specific site). Bone survivorship is visualized through pseudo-colourmaps (i.e., heatmaps). The MNE can be calculated based on the maximum number of fragment overlaps that appears in the interactive 3D viewer and is indicated on the alphanumeric module (see Supplementary File S1 for more details), which ultimately might increase the estimating accuracy of the MNI. However, it is recommended that hands-on examinations are also conducted to avoid element over- or underrepresentation due to cropping misestimations. Heatmaps consider all cropped elements, as well as the complete specimens (without cropping), as long as they meet the selected conditions. 

Among the statistical tests, several options are available for the analysis of individual datasets or the comparison between sets of bones formed for instance by different elements or carcass sizes. Graphics for the visualization of BSM distribution are provided by the creation of “time-series”-inspired datasets and their comparison, whereas statistical exploration of distribution patterns can be performed through complete spatial randomness (CSR) analysis [75,76,77], as well as the wavelet coherence analysis (WCA) [78]. Very powerful dimension reduction techniques aimed at the identification of patterns and clusters are provided by the t-distributed stochastic neighbor embedding (t-SNE) approach [79]. This is a more spatially aware, and non-linear means of performing dimensionality reduction, which could be considered more appropriate for these types of analyses as opposed to principal component analyses (PCA). t-SNE is additionally coupled with a density-based spatial clustering of applications with a noise (DBSCAN) algorithm for pattern recognition and the visualization of spatial clusters [80,81]. Various orientation analyses including the calculation, description, and comparison of linear marks (i.e., cut marks and scores) are also available. Details and other specifics regarding the statistical repertoire offered by *Ikhnos* are described in the *IkhnosToolBox* [73] documentation (Supplementary File S2).

### 2.2. Sample

**Figure 1:**
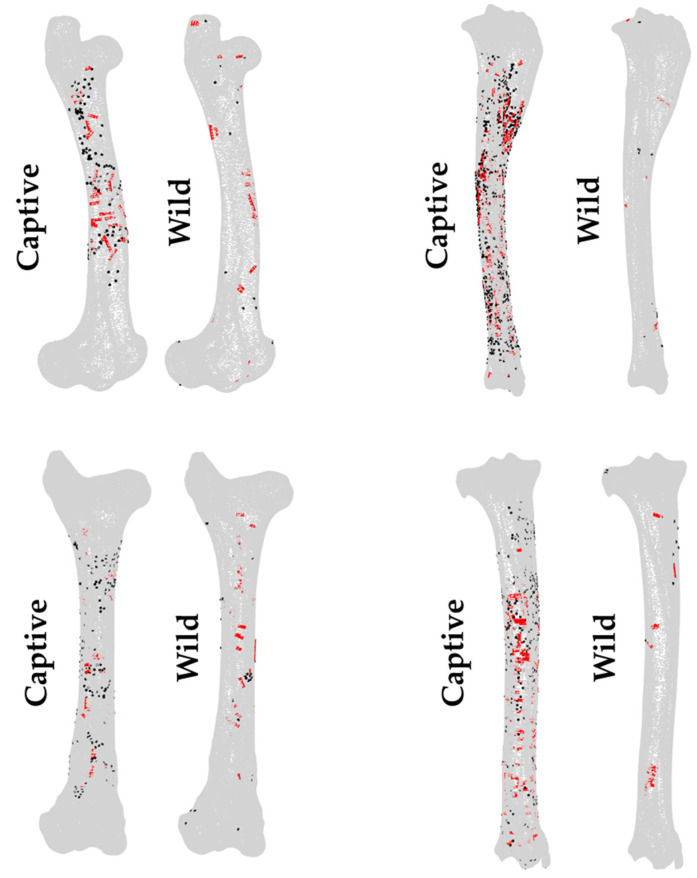
Tooth marks, including pits (black) and scores (red), digitized on the femur and tibia 3D models available in *Ikhnos* for middle-sized carcasses according to their location and orientation in the case of scores.

For the purpose of the present study, a total of 65 red deer (*Cervus elaphus*) hindlimb carcasses (including femora and tibiae) modified by Iberian wolves (*Canis lupus signatus*) were studied (see Supplementary File S3 for further details on the sample). Fifty-four carcasses stem from El Hosquillo, in Cuenca (Spain), a natural park where wolves live in captivity. The remaining 11 carcasses were modified by wild wolves from Pico Vízcares (Asturias, Spain) and Villardeciervos (Zamora, Spain). The captive sample presented a much more intense breakage than the bones consumed by wild wolves. Therefore, a previous work of refitting was performed considering size, lateralization, and bone portion. The femora (*n* = 9) and tibiae (*n* = 2) from Pico Vízcares and Villardeciervos were complete or almost complete. Bone fragments were recorded in *Ikhnos* using the editing model function and were cropped according to the available specimens. MNEs were calculated manually and using the heatmap function in *Ikhnos*, based on the maximum number of overlaps in the point cloud. Tooth pits and scores (Table 2) were also first identified with the aid of a 20x hand lens as proposed by Blumenschine and colleagues [1]. Then, the observed BSMs were documented on the corresponding 3D models (Figure 1) and analyzed using *Ikhnos*.

### 2.3. Statistical Analyses

The statistical analysis consists of three main blocks that consider (1) BSM distribution, (2) the identification and visualization of patterns within the sample, and (3) the examination of the orientation of tooth scores. Analyses were performed at different levels, first comparing the observations made on right and left elements, or within elements through the comparison of the distribution of pits and scores. Nevertheless, the main goal of the present study was the comparison of BSM patterns made by wolves in captivity and in the wild to assess how the use of reference samples obtained in captivity might alter our understanding of bone damage patterns (Figure 1). 

First, distribution patterns of BSMs were explored through CSR analyses, which aimed at describing point distribution considering the randomness, dispersion, and clustering in discrete space portions, through testing the null hypothesis (also known as Poisson point process) of point independence in space [75,76,77,82,83,84,85,86,87]. For this purpose, the 3D versions of four second-order functions were used, including the pair-correlation function (*pcf3est* from the *spatstat* R library), which estimates point intensity within defined ring ranges; the Ripley’s K function (*K3est*), which determines the number of points within multiple defined distances; the F cumulative function (*F3est*), which estimates the empty space function F(r) of a point process; and the G cumulative function (*G3est*), which derives the nearest neighbor distance G(r) of a point pattern. The square root versions of the three latter functions were also applied given the problems they might cause in inhomogeneous scenarios. Even though these tests were successfully used to identify point patterns in different scenarios, caution is required in their application, since functions for inhomogeneous patterns (e.g., *Kinhom*) are not available for 3D datasets yet, and thus all available functions assume that point distributions are homogeneous, which could end up inflating the identification of clustering patterns when inhomogeneous distributions are present. Second-order functions were used to create point envelopes by implementing a Monte Carlo simulation procedure with 500 iterations [88]. 

“Time-series” and bivariate wavelet analyses were conducted to compare the occurrence of tooth marks along a specific long bone axis (*x* = proximo-distal axis, *y* = medio-lateral axis, *z* = cranio-caudal axis), the proximo-distal axis usually being the most informative one, as well as to detect patterns through spatial redundancy in tooth mark distributions [78,89,90,91,92,93,94]. The time-series approach gives preference to the distribution variance before frequency, so that it can be efficiently used for detecting density fluctuations within samples subject to changing noise, providing the necessary basis for subsequent comparison between samples. All long bone portions were considered to bear tooth marks with equal opportunity by dividing each element into a series of units, proportional to the length of the bone. Each BSM, regardless of if it was a tooth score or a pit, was represented by a single point, with linear marks being represented by the central point. Wavelet analyses are based on the generation of sine waves to extract patterns in spectral analysis, and when considered in pairs (bivariate wavelet method) the technique is able to detect the correlation between two signals and translate it into graphics. The resulting plot is a a bidimensional heatmap indicating the oscillation of the intensity of points at specific locations. Calculations were computed through the number of periods and steps in each wavelet spectrum, and a Monte Carlo simulation approach of 10,000 iterations, which was set as the default for each pairwise wavelet analysis. Additionally, mark frequencies among the entire sample were visualized and compared using a multiple time-series plot. 

A t-distributed stochastic neighbor embedding (t-SNE) function was employed for the visualization and identification of trends in the data. The technique is based on a non-linear dimensionality reduction algorithm that constructs a low-dimensional embedding of high-dimensional data, based on the proximity of data points in multivariate feature space [79]. As opposed to PCA, t-SNE is a more spatially aware dimensionality reduction technique, being non-parametric in nature as no linear relationships are required for modeling, while neighbors in the original domain are embedded closer together in the final constructed feature space. This algorithm is accompanied by an additional non-supervised machine learning algorithm (DBSCAN), which helps define groups of marks (clusters) through a color-coded system [80]. The algorithm was trained for 1000 iterations, and the optimal perplexity parameter was calculated for each dataset as the ceiling of the square root of the sample size. The DBSCAN clustering algorithm was performed using an eps parameter of 3 (i.e., indicating how close points should be in order to be considered neighbors). Considering the stochastic nature of the algorithm, t-SNE analyses were executed several times on each dataset to obtain the most reliable overview of the results.

Tooth scores were additionally analyzed considering their orientation with respect to the principal longitudinal axis of the bone, computed using the eigendecomposition of model point clouds. First, the angles between each score and the longitudinal axis were calculated for specific samples, upon which further statistical tests were performed to characterize the distribution and variability of scores along each bone. These include normality distribution tests such as standardized skewness (a measure of asymmetry) and standardized kurtosis (a measure of tailedness and peakedness) adapted for circular data [95], while circular statistics were used to assess the existence of preferential orientations in the location and direction of tooth scores through the calculation and comparison of angles radians, using the Rayleigh uniformity test [96,97,98]. Orientation patterns among the different samples were then compared using a randomized Mardia–Watson–Wheeler test [96,99]. Considering the size differences between the wild and captive samples, a robust statistical evaluation of *p*-values was performed using the FPR (false positive risk) function included in the pValueRobust R library [100]. FPR was used to calculate Type I errors and thus address possible bias in *p*-value estimations on small sample sizes [60,101].

## 3. Results

Registration of marks, model editing, and data input in *Ikhnos* could be performed quite fast regarding the high number of BSMs digitized. The statistical analysis was performed even faster, including the tests outlined in Supplementary File S3. 

**Figure 2:**
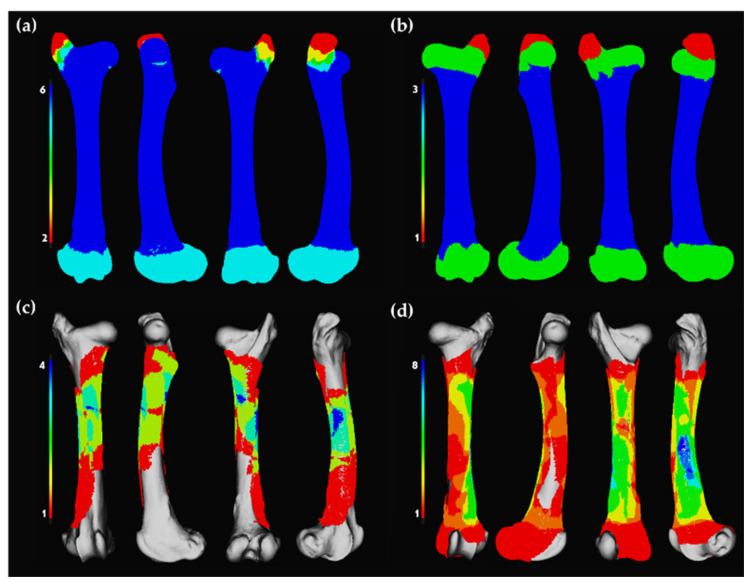
Heatmaps generated in *Ikhnos*. Bone preservation for right and left femora modified by wolf populations divided by (**a**) right femora modified by wild wolves; (**b**) left femora modified by wild wolves; (**c**) right femora modified by captive wolves; (**d**) left femora modified by captive wolves. Red-shaded portions indicate areas of lowest survivorship, whereas blue-shaded portions indicate areas of highest survivorship, namely the MNE.

**Figure 3:**
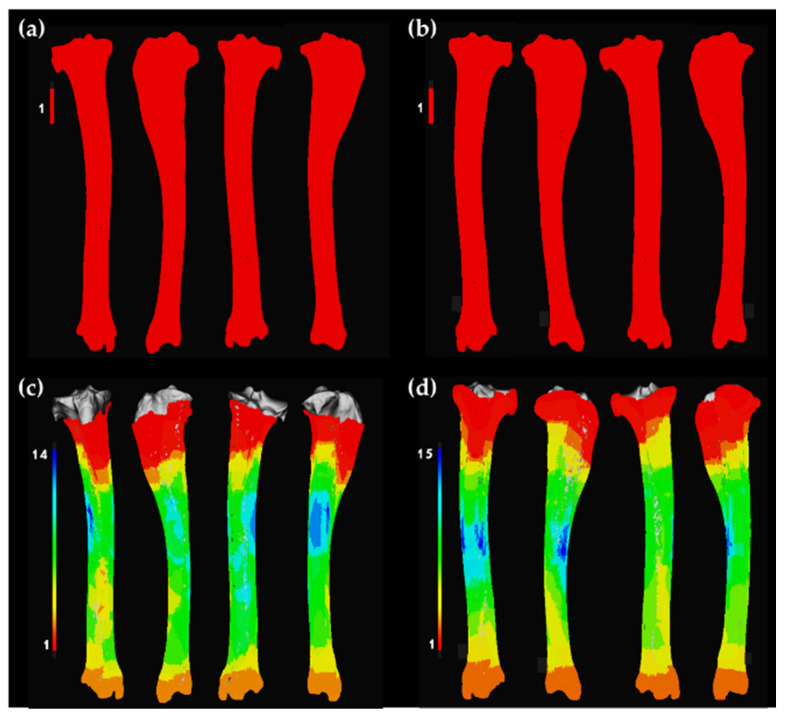
Heatmaps generated in *Ikhnos*. Bone preservation for right and left tibiae modified by wolf populations divided by (**a**) right tibiae modified by wild wolves; (**b**) left tibiae modified by wild wolves; (**c**) right tibiae modified by captive wolves; (**d**) left tibiae modified by captive wolves. Red-shaded portions indicate areas of lowest survivorship, whereas blue-shaded portions indicate areas of highest survivorship, namely the MNE. In the case of the wild wolf populations, the highest and lowest survivorship is the same.

MNE calculations performed with the *Ikhnos* heatmap function matched exactly those calculated manually for the wild sample by experts, with six right femora and three left femora, as well as two tibiae, one left and one right (Figure 2 and Figure 3). For the sample generated by wolves in captivity, the results were found to slightly differ, probably due to the substantial damage inflicted by these populations that resulted in high breakage patterns. While calculations coincide in the MNE for the femora (right = 4 elements; left = 3 elements; Figure 2), in the case of the tibiae, MNE estimates differ by one element (Figure 3), being that the calculation performed by *Ikhnos* was always lower (right = 14 elements; left = 15 elements) than the one calculated manually by experts (right = 15 elements; left = 16 elements). 

**Figure 4:**
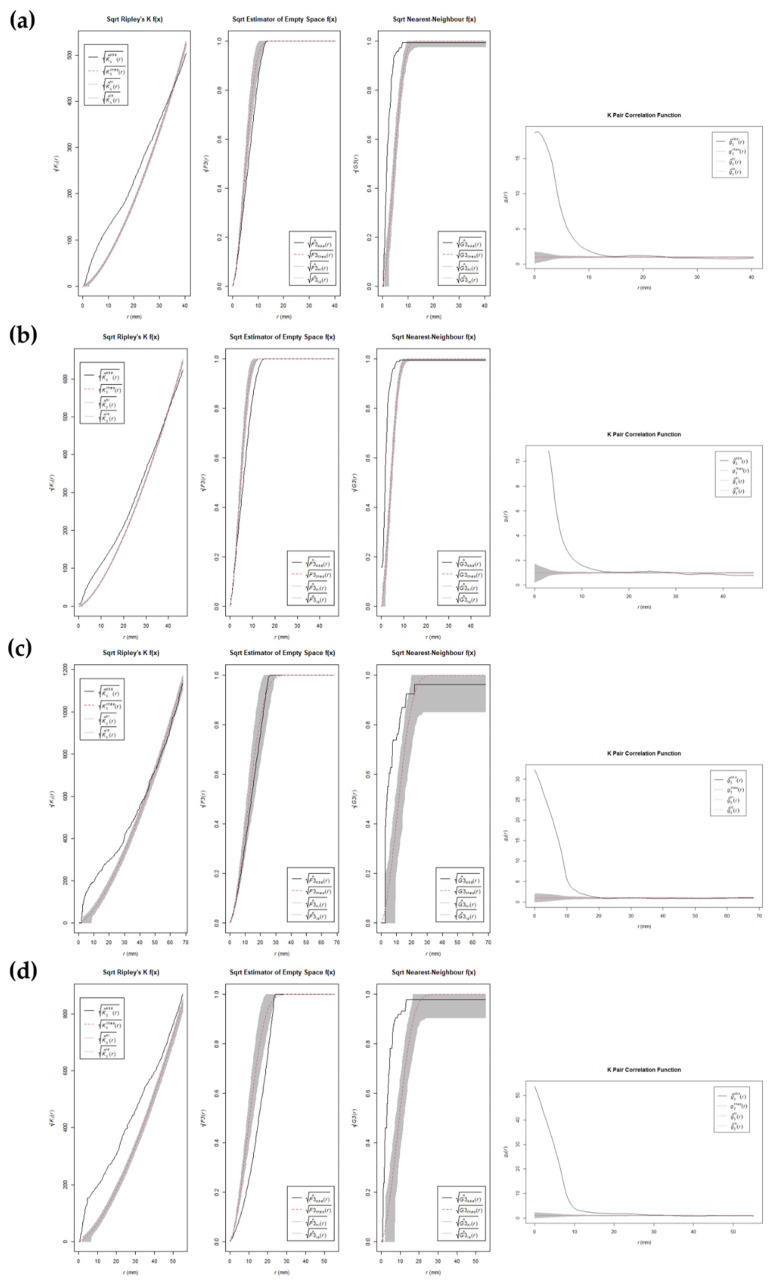
Plots of the square root version of the K-function, F empty space function, G near-neighbor function, and pair-correlation function for (**a**) tooth marks on right femora generated by captive wolves; (**b**) tooth marks on left femora generated by captive wolves; (**c**) tooth marks on right femora generated by wild wolves; (**d**) tooth marks on left femora generated by wild wolves. Dotted red line shows the Poisson complete spatial random (CSR) process, the black line shows the empirical point process of the target sample, and the gray band shows simulated confidence intervals.

**Figure 5:**
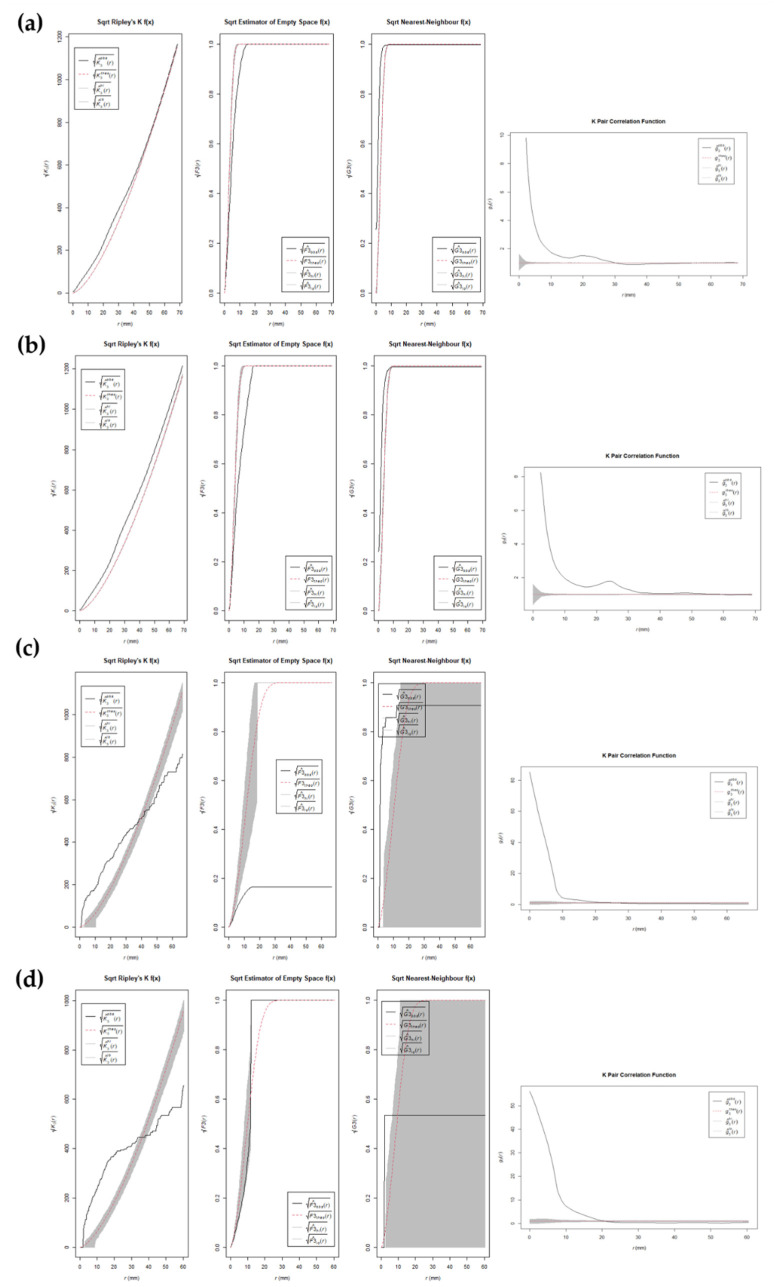
Plots of the square root version of the K-function, F empty space function, G near-neighbor function, and pair-correlation function for (**a**) tooth marks on right tibiae generated by captive wolves; (**b**) tooth marks on left tibiae generated by captive wolves; (**c**) tooth marks on right tibiae generated by wild wolves; (**d**) tooth marks on left tibiae generated by wild wolves. Dotted red line shows the Poisson complete spatial random (CSR) process, the black line shows the empirical point process of the target sample, and the gray band shows simulated confidence intervals.

CSR functions suggest mild clustering trends in the distribution patterns of BSMs regardless of the living context of wolves, the bone element, its laterality, and the type of mark, i.e., tooth score or pit (Figure 4 and Figure 5, and Figure S1–S4). Subtle differences can be observed between left and right elements. However, results must be considered with caution. First of all, the sample size in certain cases is quite small (e.g., tibiae modified by wild wolves), which results in large confidence intervals. Second of all, the assumption of homogeneity in point distribution by the second-class CSR functions applied here might be exaggerating the presence of clusters in inhomogeneous distribution patterns, which are prone to appear in the case of natural processes such as the ones studied here.

**Figure 6:**
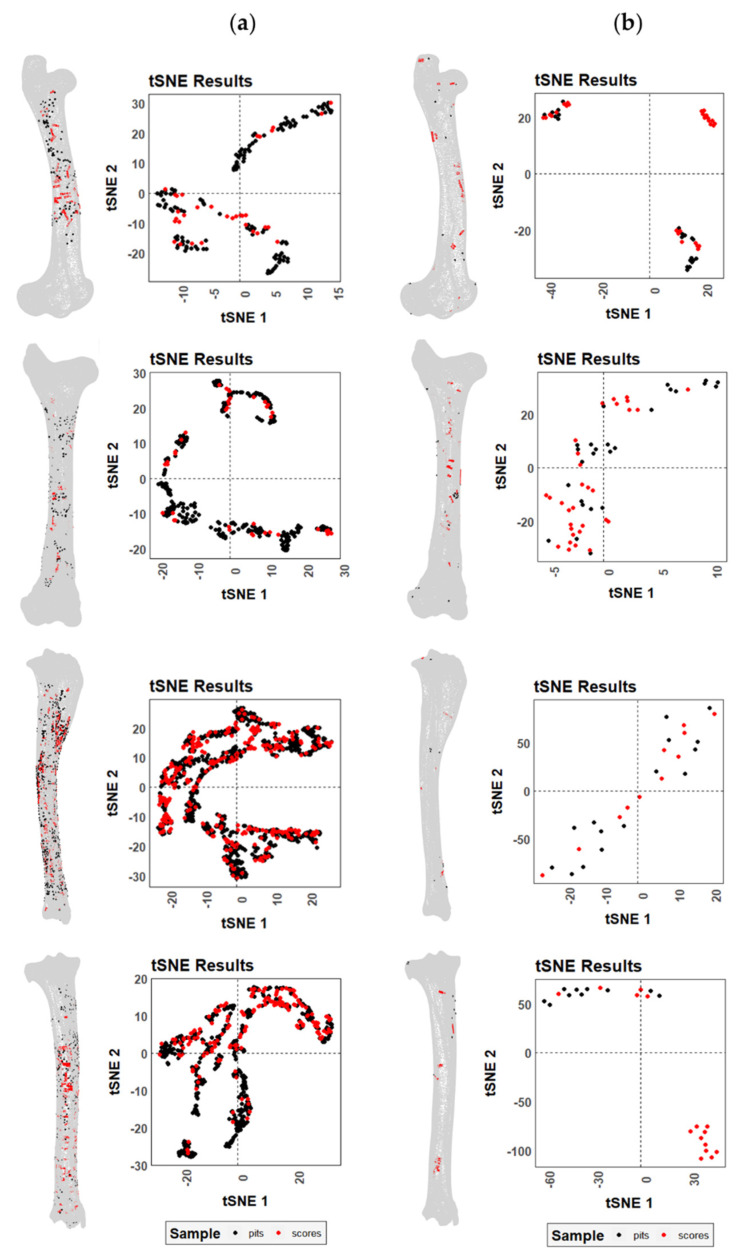
t-SNE scatter plots for both tooth scores and pits on the right and left femora and tibiae modified by (**a**) captive and (**b**) wild wolves.

When comparing the distribution of tooth scores and pits on the femora and tibiae modified by wild and captive wolves, two different trends can be observed depending on the living context of the animals. The t-SNE plots (Figure 6) show no clear distinction between pits (black) and scores (red) on the carcasses modified in captivity, whereas there are some slight differences regarding the location of tooth scores and pits generated by wild wolves. As highlighted by the graphs, both tibiae and femora present areas where scores appear in isolation, whereas pits always tend to be accompanied at least by some scores.

**Figure 7:**
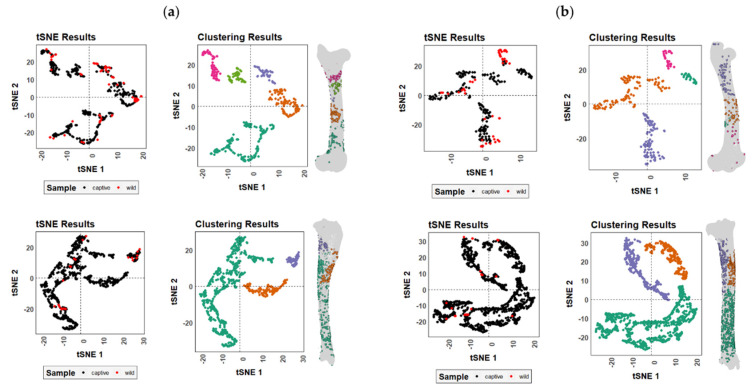
t-SNE scatter plots for both tooth marks on the (**a**) left and (**b**) right femora and tibiae modified by captive and wild wolves. Different colors in the clustering results plots indicate clustering patterns identified by DBSCAN.

Differences in BSM distribution patterns generated by wild and captive wolves are more striking, though not sufficient enough to distinguish two separate groups (Figure 7). As expected by the large number of BSMs documented on the carcasses modified by wolves in captivity, all those areas of high damage intensity in the wild sample were also modified in the captive sample. Nevertheless, slight differences in the anatomical distribution of marks along the femora can be observed (Figure S5), probably in relation to the lower bone survivorship in captive environments (Figure 2). The distal and proximal epiphyses are poorly represented in the femoral captive sample due to intensive gnawing. The lack of these bone portions and the preservation of midshaft areas in the captive sample resulted in the underrepresentation of BSM patterns in the bone ends. Meanwhile, the wild sample contains complete or almost complete elements with marks on the epiphyses and not-so-heavily modified diaphyseal portions. Similarly, the identification of patterns regarding the location of BSMs is clearer in the wild sample than in the captive sample where the intense overlap of marks might obscure the areas of interest (Figures S5 and S6).

**Figure 8:**
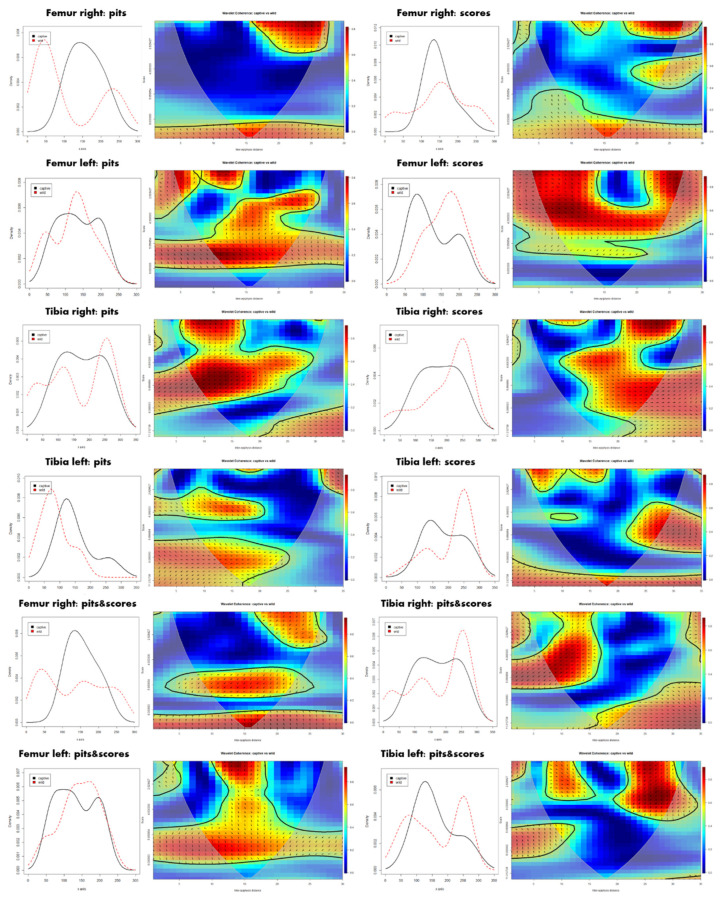
Pair histograms of tooth mark occurrence along the longitudinal bone axis and bivariate wavelet coherence plots showing the correlation of tooth modifications produced by wild and captive wolves on the proximal, midshaft, and distal portions of the analyzed femora and tibiae. The horizontal x-axis refers to the inter-epiphyseal distance from the distal to the proximal end of each bone, while the vertical y-axis refers to the scale. Correlation between both datasets is pseudo-color coded, with the correlation represented in red and no dependence indicated in blue. Arrows represent the lead/lag phase relations between the datasets. Arrows pointing to the right (→) indicate “time-series” to be in phase, i.e., they covary in the same direction. Arrows pointing to the left (←) indicate “time series” to be in anti-phase, i.e., samples change in opposite directions. Arrows pointing to the bottom-right or upper-left indicate that the first variable (captive) is leading, while arrows pointing to the upper-right or bottom-left indicate that the second variable (wild) is leading.

Differences in distribution patterns between the captive and the wild samples are more obvious when considering raw coordinate data extracted from the digitized BSMs. According to the histograms (Figure 8), captive wolves tend to generate a more balanced damage pattern along the whole carcass, which usually modifies the epiphyseal and metaphyseal portions more intensively. The occasional accumulation of BSMs in the mid-shaft segment in the captive sample might rather respond to the deletion of the epiphyses and the subsequent impossibility of registering marks on epiphyses. The more intensive modification of epiphyseal portions by wild populations of wolves, however, shows certain differences considering the location of tooth pits and scores, as well as bone lateralization (Figure 8). Differences in the cranio-caudal and medio-lateral axes are less pronounced since the portion considered is much smaller (Figures S7–S9).

Differences in the documentation of BSMs due to preservation processes, or due to intrinsic dissimilarities in relation to the element side, result in different correlation patterns. This is notable through the biwavelet plots in Figure 7. For instance, the right femora show the widest occurrence of randomness among all elements, especially when pits are considered in isolation. On the contrary, redundant clustering in the left femora is marked in a more continuous way along the whole element, particularly in the mid-diaphyseal portion. The correlation in tibiae is more dispersed, while a subtle pattern can be recognized when considering pits and scores in isolation. While redundancy among pits is more marked in the proximal half of the bone, the opposite is true for scores. The correlation among tooth mark patterns can be positive and negative, which means that sometimes results are in phase and thus changes are in the same direction (i.e., BSM intensity increases or decreases in both samples as in the distal portion of the right tibia), while other times patterns are in the so-called anti-phase, where BSM intensity changes in opposite directions (i.e., see pit distribution on the mid-shaft of the right femur).

**Figure 9:**
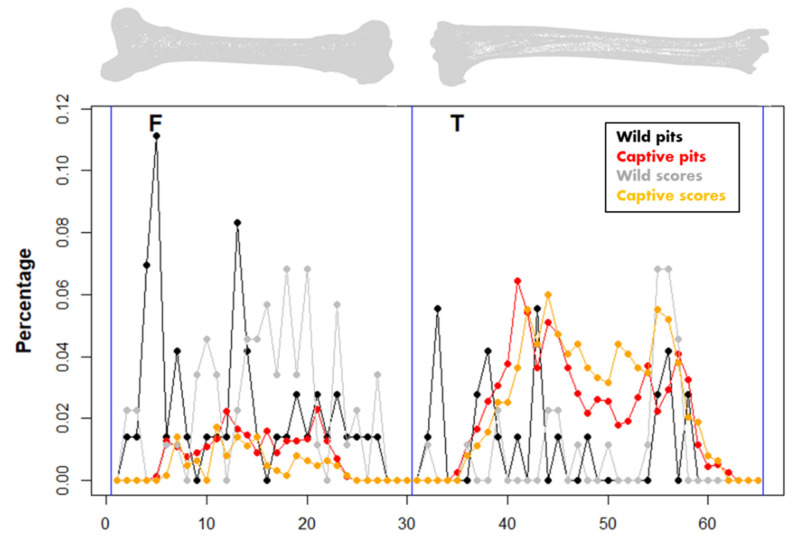
Average relative distribution of tooth marks obtained, including pits and scores, in the wild and captive wolf samples. The x-axis shows the longitudinal dimensions of the series of the left and right femur (**F**) and the tibia (**T**) from the proximal to the distal epiphysis sequentially.

Distribution patterns of the two bones show that carcasses modified by wild wolves present much more clustered BSM patterns than carcasses modified by captive populations, where damage occurrence is more balanced throughout the entire bone, but for the epiphyseal portions that have been deleted (Figure 9). Interestingly, BSM is also more variable depending on the type of tooth mark, score, or pit in wild samples, with more pits on the proximal half of the femur and tibiae, and an increase in tooth scores in the distal half of both elements. Notable differences can also be observed when elements are lateralized (Figure S10).

**Figure 10:**
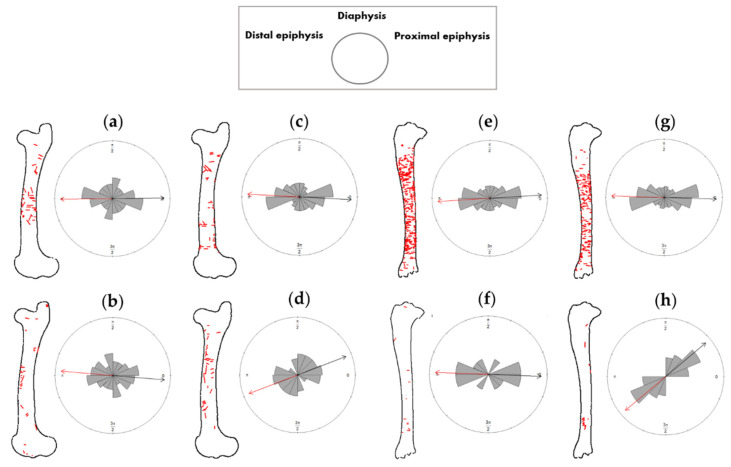
Rose diagrams obtained for the calculated orientation patterns between the documented tooth scores (red lines) and the corresponding longitudinal bone axis of the (**a**) right femora modified by captive wolves; (**b**) right femora modified by wild wolves; (**c**) left femora modified by captive wolves; (**d**) left femora modified by wild wolves; (**e**) right tibiae modified by captive wolves; (**f**) right tibiae modified by wild wolves; (**g**) left tibiae modified by captive wolves; (**h**) left tibiae modified by wild wolves. The box legend indicates the rose diagram preferential orientation (from distal to proximal long bone epiphysis).

The orientation patterns of tooth scores generated by wild wolves are quite variable regarding the bone side (Figure 10). Captive populations, on the contrary, seem to end up generating more standardized scores in terms of orientations when laterality is considered, which also usually results in less pronounced preferential orientations. Differences in this sense are more marked in the tibia sample, where robust statistics have verified the significance (*p*) value for the calculated preferential orientation of the wild score sample (FPR < 0.008%). On the other hand, the femora show higher degrees of randomness (e.g., the left femur shows opposite trends in comparison to the rest of the elements). Despite the differences observed, no significant differences were detected in the pairwise comparison between the left and right elements, all with *p*-values = 1, and neither were differences significant when comparing wild and captive samples (Table 3). In fact, in most elements analyzed here, tooth scores are commonly oriented either at right angles or diagonal to the long axis of the bone, regardless of the living context of the wolf packs.

## 4. Discussion

The *Ikhnos* 3D software provides an open-access tool to extract three-dimensional anatomical spatial information of BSMs by enabling their documentation, digitization, and statistical analysis. Thus, *Ikhnos* meets the demands stated by previous authors [36,45,102] regarding the necessity of increasing the registration specificity of BSMs in order to be able to correctly identify carcass access in the fossil record. 

In the present study, the use of this software has helped in the registration of the subtleties of BSM distribution on deer carcasses consumed by wild and captive wolf populations, as well as their subsequent analyses thanks to the incorporation of the *IkhnosToolBox* R library [73], where dependencies of the *spatstat* [88], *biwavelet* [103], *circular* [104], and *Rtsne* [105] libraries were included. The incorporation of this powerful analytical basis enables the extraction and identification of BSM distribution patterns, merely based on statistical analyses, as well as a comprehensive inter-element approach that enables the consideration of different skeletal elements in the analyses. In doing so, the present work offers a refined statistical depth in comparison to other methods. Additionally, *Ikhnos* can also help in calculating essential parameters for taphonomic studies such as the MNE and MNI, thanks to the “overlap of specimens” approach and heatmap calculations based on maximal and minimal bone portion survivorship.

Prior to the present study, Parkinson and colleagues [45] documented what is, to date, the largest experimental assemblage of canid (*C. lupus baileyi* and *C. rufus*) BSMs using a bidimensional GIS-based approach provided by ArcGIS. In their study, they identified a significant pit scattering variation across elements and across bone portions, that results in the appearance of preferential mark clusters on all long bones. Similarly, certain location preferences regarding tooth scores were observed among elements. The repeated location of BSMs is often in accordance with the position of meaty areas, and might ultimately correspond to a certain standardization during carcass consumption. For their feeding experiment, these authors selected captive wolf populations with minimal contact with humans, expecting that they might be a suitable analogue for wild groups and thus not generate the increased tooth mark frequencies previously observed in captive environments by other authors [57,60,65,71]. However, in these semi-wild environments group mobility is limited, which could also affect the population behavior. 

In their study, Parkinson and colleagues [45] insist on the “importance of separating analyses by element as well as bone portion” in order to be able to distinguish carnivores based on the BSM distribution patterns they produce, and consider their approach to be the best suited for comparison with other carnivore patterns or fossil samples. In the present work, apart from separating among elements, the registration of BSMs was performed considering lateralization, which might provide a more in-depth comprehension of consumption processes when considered alongside bone survivorship, as it might be relevant in cases of asymmetrical damage intensity or differential bone preservation. In fact, bone destruction has already been pointed out as particularly relevant for the identification of carnivore agency [9,14,64,106,107,108], and it appears to be so for the interpretation of BSM patterns as well. 

According to Parkinson and colleagues’ [45] results, the destruction of one or both epiphyses is a common signal of consumption by large canids in the wild. Our results agree that shaft cylinders are the most usual form of bone preservation, though epiphyseal deletion proportions differ among studies. In both experiments, the greater trochanter of the femur is very typically removed, while the head of the femur is more often preserved, especially in the present case. On the contrary, the distal epiphysis is more commonly deleted in Parkinson and colleagues’ study than in ours, where the femoral condyles are preserved at intermediate to high frequencies. From a more classical taphonomic perspective, these observations coincide to some extent with data known on wolf long bone modification patterns [109], supporting some of the previous data already published regarding these samples by Yravedra and colleagues [67].

In both Parkinson’s and our studies, there is a dense concentration of tooth pits on the femoral head, but an increased amount of tooth marks (pits and scores) along the midshaft could be observed in our sample when compared to [45]. Additionally, few pits and scores were also recorded on the distal epiphysis in contrast to the virtual lack of BSMs in this portion in previous studies. In some cases, the lack of BSMs can be explained by the common deletion of specific bone areas, as highlighted by the marked differences in BSM distribution patterns when comparing the wild and captive samples. The femur sample modified by captive groups includes no proximal epiphysis (greater trochanter and head included), and almost no distal epiphysis (only one condyle of one left femur was preserved). Therefore, despite the generalized higher frequency of tooth marks along the femoral shaft, no pit or score was recorded in any of the epiphyses. Experiments in captivity might thus not be adequate to establish carnivore BSM models that serve as a reference for archaeological or paleontological studies, not only because of the exaggerated increase in BSM frequencies due to “recreational gnawing”, but also due to the total disappearance of bone portions that in other contexts may persist and bear marks. Such observations were already noted by Campmas and Beauval [57], who found that the damage inflicted by captive wolf populations on cattle carcasses (*Bos taurus*) could not be distinguished from the pattern produced by wild hyenas. 

In [45], the proximal portion of the tibia is also typically deleted, while in our sample, tibiae consumed by wild wolves are intact. Nevertheless, such differences might be caused by the very low representation of tibiae in our sample (*n* = 2). The pattern previously observed by Parkinson and colleagues is nevertheless detected in the case of captive populations (*n* = 29), where bone damage is much more pronounced in general terms. The highest concentration of BSMs produced by wild wolves was recorded in the distal shaft portion, whereas Parkinson and colleagues found the densest concentrations on the proximal shaft portion of the tibia. However, they also recorded intensive tooth pitting in the distal end. BSM patterns in our sample differ when marks are divided into the mark type (score or pit) and when the elements are lateralized. In doing so, we recorded a concentration of tooth pits on the proximal shaft, while scores were mainly located on the distal portion in the left tibia. In the captive sample, two trends could be observed, though, in general, tibiae are characterized by displaying very high frequencies of marks along the entire shaft. While the right tibiae show a more balanced ratio of tooth pits to scores along the whole diaphysis, the left tibiae bear more marks (especially pits) in the proximal half. 

All in all, the identification of patterns regarding the location of BSMs is more straightforward based on feeding experiments on wild populations than on groups in captivity, since recreational gnawing might have produced an overrepresentation of BSMs in areas without nutritional interest. Additionally, according to the t-SNE plots, both tibiae and femora consumed by wild wolves indicate certain patterns in the location of tooth scores and pits that could be associated with different consumption activities (e.g., slicing or crushing) on specific portions of the elements. While there are some areas where scores appear in isolation, pits always tend to be accompanied by at least a few scores. These results are in line with previous observations based on tooth morphology [56], according to which current wolf populations (i.e., *C. lupus*) present an increased slicing activity in comparison to ancestral groups. Specific trends regarding the orientation of tooth scores with respect to the long bone axis, which were previously observed in other studies [45,64,107], could be assessed in the present study through statistical analyses. Results in this line agree with other authors’ observations in that tooth scores usually occur at right angles or diagonally to the longitudinal bone axis.

Apart from the possible behavioral differences between extant and extinct canid groups, also current and past trophic scenarios have to be accounted for before extrapolating feeding experiments to interpret the fossil record. Despite the remarkable differences in BSM frequencies reported here for the captive and wild samples, it must be also noted that even the total number of recorded marks among wild wolf populations might greatly differ from those found in the archaeological or paleoanthropological record (see for instance [66]). Fossil assemblages are not only commonly affected by abiotic processes that might impact bone survivorship, but there are usually other agents intervening in the assemblage (e.g., humans or other carnivores), which in turn increases feeding competition and thus might reduce meat exposure time and impede unique access to carcasses. 

The present work presents a novel way of documenting and analyzing carcass modifications that provides a more comprehensive (quantitative) approach to the identification of element-specific patterns which can be statistically identified. In doing so, *Ikhnos* is able to generate carnivore-specific taphonomic models beyond those established previously, either for canids [45,67], felids [9,46,110], or hyenidae [111,112]. The definition of the 3D taphonomic signature of modern carnivores may contribute to a better understanding of hominin–carnivore interactions. First of all, in opposition to bidimensional templates, BSM registration in *Ikhnos* is more accurate at the spatial level thanks to the use of a 3D bone template that is a replica of the real bone. This way of documenting marks also allows addressing BSM patterns as point processes by means of statistics. Second of all, previous GIS-based approaches are still dependent on visual comparisons and on manually set parameters [113], instead of on statistical techniques such as those provided by *Ikhnos*, or if maps were to be calculated using statistical libraries [88]. Users may thus play much a bigger role in the detection of clustering patterns within the point process when employing former methods. Analytical differences are also related to the measurement of intensity; while the kernel map approach available in software solutions such as ArcGIS is based on absolute intensity location, the methods included in the *Ikhnos* software consider BSM distribution proportionally. Thus, kernel maps might also underestimate the relevance of certain marks for pattern detections as weight is placed on clusters, whereas the consideration of relative mark distributions enables *Ikhnos* to detect patterns irrespective of sample size. Finally, the analytical tools incorporated in TIPZOO [52] do not specifically deal with BSM distribution patterns, as this software solution does not allow for point registration of marks, but it provides a more generalized documentation platform for zooarchaeological data (e.g., skeletal part representation, calculation of MAU indexes).

Regardless of the nature of the differences (e.g., feeding competition within populations, prey size, wolf group size, sample size) observed between previous studies and the present one regarding canid carcass modification, this study remarks the importance of (1) suitably selecting the sample provenance (e.g., carnivores in the wild versus in captivity), and (2) properly registering, quantifying, and analyzing mark distribution, in order to be able to address agency and behavioral questions. For instance, the accurateness provided by the use of 3D bone models to register BSMs and their analysis beyond the sample size provide a compact toolkit for the study of bone assemblages when statistical results are considered alongside anatomical (i.e., the location of muscles, the ergonomics of human butchering practices), ethological, and biomechanical data.

All in all, *Ikhnos* provides a novel and more objective way of registering, analyzing, and comparing BSM anatomical distributions, and thus the best way to elaborate comprehensive taphonomic databases and referential models (e.g., experiments on single or multiple carnivore carcass access, the impact of abiotic factors such as trampling) that can be then used to interpret the fossil record.

## 5. Conclusions

Several authors have stated the necessity of improving the documentation and analysis of bone surface modifications (BSM) in taphonomic studies as they are crucial in understanding site formation processes. Considering the current shortcomings of the field in this line, the present work has introduced a novel software for the digital registration and statistical analysis of BSM using 3D point patterns and long bone models.

The application of the functions provided by *Ikhnos* to a case study on wild and captive wolf populations has demonstrated not only the problems regarding the use of carnivores in captivity for the generation of referential models that may be used to make inferences on the fossil record, but it has particularly highlighted the utility of this novel software and the contributions its use may provide to future studies on BSM through the generation of accurate neotaphonomic referential models and the analysis of archeological and paleontological remains.

## Figures and Tables

**Table 1:** Comparison of software packages designed for the registration and analysis of BSMs.

Software	Bone Elements	Data Collection	Statistics	Access
ArcGis ^1^ (ArcMap) ^1^	All types	•Bone survivorship is registered via digital drawing over photographic 2D templates of complete bones •BSMs are plotted on separated rasterized 2D bone templates for anterior, posterior, mesial, and lateral views of each element	Spatial analyses including nearest neighbor distance, kernel density estimation	License fee
TIPZOO ^2^	All types	•Bone survivorship and BSM documented through codes according to skeletal element, segment and orientation	Data export for statistical analysis in separate software tools, not suited for point-based spatial statistics	Open Access
*Ikhnos*	Long bones	•Bone survivorship is registered via digital polygonal cropping on the 3D point mesh and the calculation of heatmaps •BSMs are documented through the selection of landmarks *(x,y,z*) on the 3D point mesh of each element	Integrated R library *IkhnosToolBox* that includes 3D spatial and multivariate statistical analyses, tests for point patterning identification and visualization, and circular analyses for linear BSMs	Open Access

^1^ License [53]. ^2^ License [52].

**Table 2:** Tooth marks identified in the El Hosquillo, Pico Vízcares and Villardeciervos samples.

Sample	Femur				Tibia			
	Right (C = 4, W = 6)		Left (C = 8, W = 3)		Right (C = 14, W = 1)		Left (C = 15, W = 1)	
	pits	scores	pits	scores	pits	scores	pits	scores
Wild wolves (W)	22	29	26	32	15	11	10	16
Captive wolves (C)	130	39	240	45	769	363	431	188

**Table 3:** Results obtained for the orientation analyses performed on the tooth scores identified on the deer femora and tibiae modified by captive and wild wolves.

Statistics	Right Femur		Left Femur		Right Tibia		Left Tibia	
Test	Captive	Wild	Captive	Wild	Captive	Wild	Captive	Wild
Skewness	−0.8651	0.3926	0.1974	0.9063	0.1824	−1.0769	−0.1009	−1.7587
Kurtosis	−0.1101	−0.6033	1.1583	0.9208	1.816	3.0222	3.6393	0.7149
Circular variance	0.2741	0.2523	0.1884	0.2535	0.1671	0.0827	0.1266	0.0681
Circular dispersion	0.6676	0.6451	0.3982	0.5497	0.3373	0.1609	0.2357	0.1392
Central orientation radians	−3.13/0.01	3.06/−0.08	−0.05/3.09	0.36/−2.78	0.06/−3.08	−0.04/3.1	−0.02/3.12	0.7/−2.44
Central orientation degrees	−179.3/0.7	175.2/−4.8	−3.1/176.9	20.9/−159.1	3.6/−176.4	−2.1/177.9	−1.2/178.8	39.9/−140.1
Preferential orientation	0.7258 (1.0 × 10^−9^)	0.7477 (3.5 × 10^−8^)	0.8116 (6.9 × 10^−13^)	0.7465 (1.0 × 10^−8^)	0.8329 (4.4 × 10^−110^)	0.9173 (0)	0.8734 (5.1 × 10^−63^)	0.9319 (5.0 × 10^−7^)
Orientation comparison	6.7226 × 10^−31^ (*p* = 1)		4.8088 × 10^−31^ (*p* = 1)		2.3845 × 10^−30^ (*p* = 1)		1.974 × 10^−31^ (*p* = 1)	
	Captive Femora: 6.1859 × 10^−31^ (*p* = 1) Wild Femora: 4.8418 × 10^−31^ (*p* = 1)				Captive Tibiae: 3.3887 × 10^−30^ (*p* = 1) Wild Tibiae: 4.8725 × 10^−31^ (*p* = 1)			

## Data Availability

To support open science, the *Ikhnos* Installer and the integrated R library *IkhnosToolBox* were uploaded to the research team GitHub page (https://github.com/TIDOP-USAL (accessed on 19 October 2022)) and are freely accessible. The *Ikhnos* installer is available on https://github.com/TIDOP-USAL/IkhnosApp/releases (accessed on 19 October 2022) and the *IkhnosToolBox* R library is available on https://github.com/TIDOP-USAL/IkhnosToolBox (accessed on 19 October 2022). User’s guides for both *Ikhnos* and *IkhnosToolBox* are provided as Supplementary Files.

## Supplementary Materials

The following supporting information can be downloaded at: https://www.mdpi.com/article/10.3390/ani12202861/s1, Supplementary File S1: *Ikhnos* User’s Guide, containing the instructions for the *Ikhnos* software setup and a brief explanation of the available functions [74,114]. Supplementary File S2: *IkhnosToolBox* Documentation, containing a brief documentation of the functions available in the *IkhnosToolBox* R library. Supplementary File S3: Case study, containing a more in-depth explanation of the sample provenance and additional figures (Figures S1–S10) on the wild and captive wolf populations case study[29,58,59].
