# Supplementary material for: Ikhnos: A Novel Software to Register and Analyze Bone Surface Modifications Based on Three-Dimensional Documentation
Source: Animals (Basel). 2022 Oct 20;12(20):2861. doi: 10.3390/ani12202861 (PMC9598086; doi:10.3390/ani12202861)
Supplement: Supplementary file 1 [file animals-12-02861-s001.zip › Animals_S1.pdf]

Ikhnos: a novel software to register and analyse bone surface modifications based on three-dimensional documentation

Supplementary File S1: *Ikhnos* User's Guide

Rocío Mora [1], Julia Aramendi [1,2], Lloyd A. Courtenay [1,3], Diego González-Aguilera [1*], José Yravedra [3,4], Miguel Ángel Maté-González [5,1], Diego Prieto-Herráez [6,1], José Mª Vázquez-Rodríguez [7], Isabel Barja [8,9]

[1] Department of Cartographic and Land Engineering, Higher Polytechnic School of Avila, Universidad de Salamanca, Hornos Caleros 50, 05003, Ávila, Spain

[2] Department of Geology, Facultad de Ciencia y Tecnología, Universidad del País Vasco - Euskal Herriko Unibertsitatea (UPV/EHU), Barrio Sarriena S/n, 48940 Leioa, Spain

[3] Department of Prehistory, Ancient History and Archaeology, Universidad Complutense de Madrid, Prof. Aranguren 8 s/n, 28040, Madrid, Spain

[4] C. A. I. Archaeometry and Archaeological Analysis, Universidad Complutense de Madrid, 28040, Madrid, Spain

[5] Department of Topographic and Cartography Engineering, Higher Technical School of Engineers in Topography, Geodesy and Cartography, Universidad Politécnica de Madrid, Mercator 2,28031 Madrid, Spain

[6] Institute of Fundamental Physics and Mathematics, Merced Building, Universidad de Salamanca, Plaza de la Merced 1, 37008, Salamanca, Spain

[7] Department of Prehistory and Archaeology, Humanities Faculty, UNED, C/Senda del Rey, 7, 28040 Madrid, Spain

[8] Zoology Unit, Department of Biology, Universidad Autónoma de Madrid, C/Darwin 2, Campus Universitario de Cantoblanco, 28049 Madrid, Spain

[9] Center of Investigation in Biodiversity and Global Change (CIBC-UAM), Universidad Autónoma de Madrid, 28049 Madrid, Spain

*Correspondence: daguilera@usal.es

The present document contains the instructions for the ***IKHNOS*** set up and a brief explanation of the available functions.

Contents

1. Software Setup
   1.1. IKHNOS Setup
   1.2. R and Database Configuration

2. *IKHNOS* utilities
   2.1. Documentation and registration
   2.2. Bone survivorship
   2.3. Statistics

1. Software Setup

In this guide, all the necessary steps to be able to install and use the *IKHNOS* software will be explained.

First of all, users must download the ***All in One*** Installation file, which includes *IKHNOS* and third-party software pieces such as R and XAMPP. The ***All in One Installer*** is available in the following link (https://github.com/TIDOP-USAL/IkhnosApp/releases). *IKHNOS* is nowadays only available for Windows operating systems.

1.1. *IKHNOS* Setup

All the necessary files are compressed in the single folder (**IKHNOS_5_0_Windows_x64**) available in the link mentioned above.

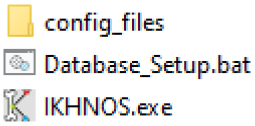

The **IKHNOS_5_0_Windows_x64 folder has to be located in the Documents Folder** of the PC before starting the installation process.

To start installing *IKHNOS*, users have to click on the **IKHNOS.exe** file using the **right mouse button** and select the option **Run as Administrator**. Once opened, the Installer will guide users through the installation process. Before installing *IKHNOS*, users must decide whether to create a Desktop shortcut or not.

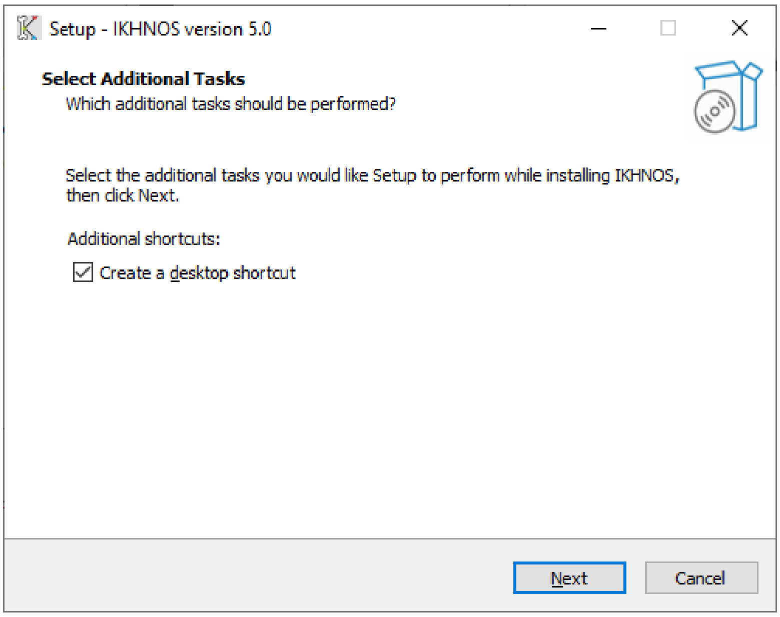

The installation will commence after clicking on the **Install** button.

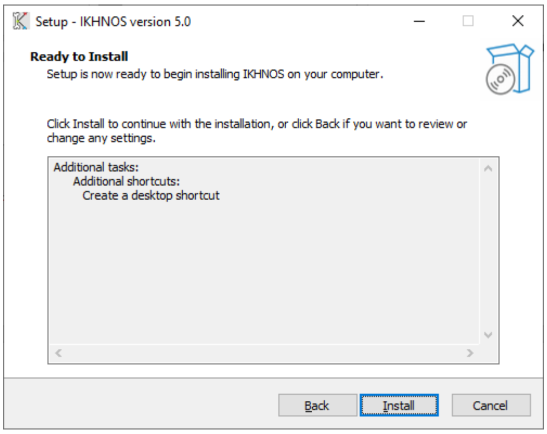

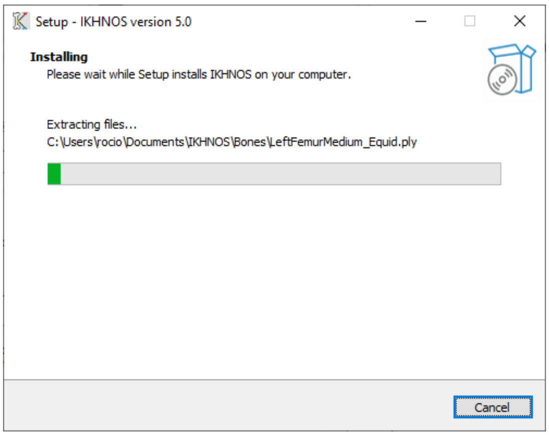

After installing *IKHNOS* main tools, the **R tools installation** starts. The R setup requires the **selection of the main language,** the **acceptance of the terms and conditions** and the **selection of the installation folder.**

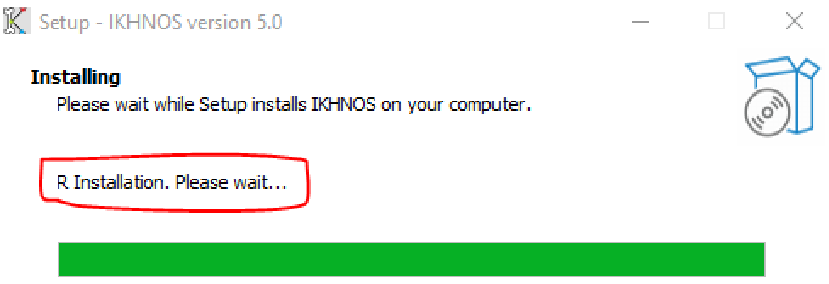

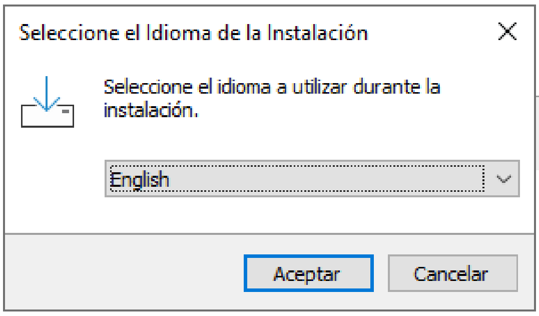

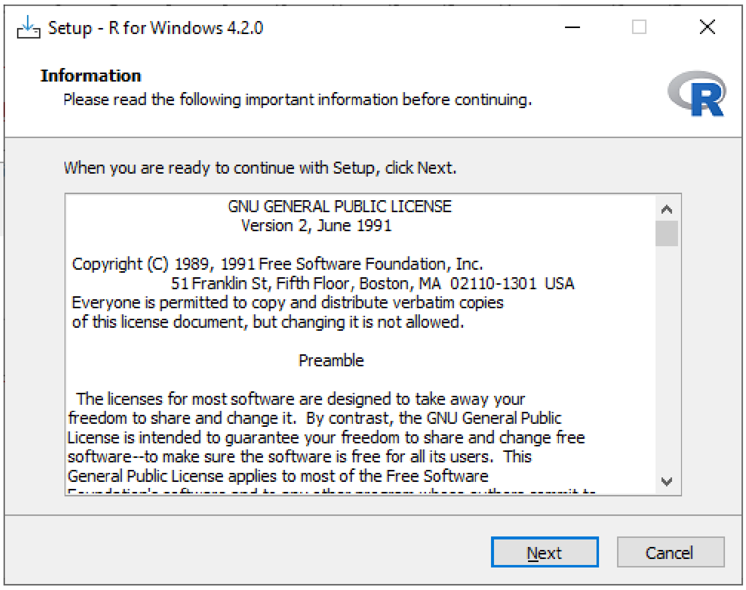

Users must **install R in the Documents folder (C:\Users\<username>).** The final path should be **C:\Users\<username>\Documents\ R-4.2.0**

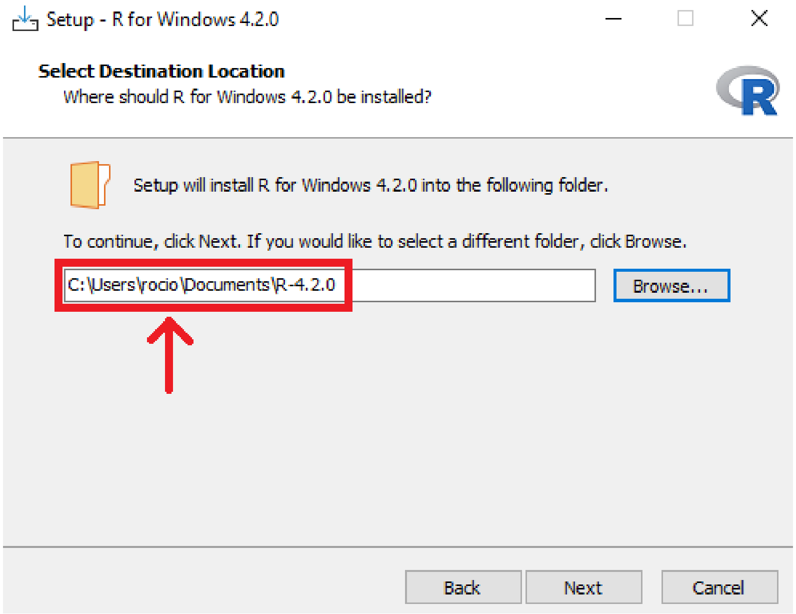

After selecting the installation path, users have to click on the **Next** button and continue to do so without altering any parameters in any of the coming windows, in order to **start the installation process**.

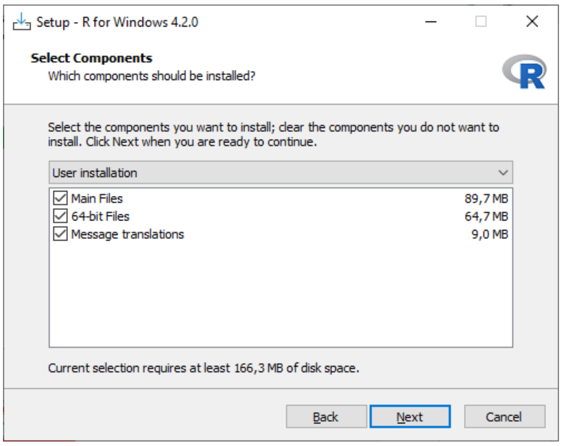

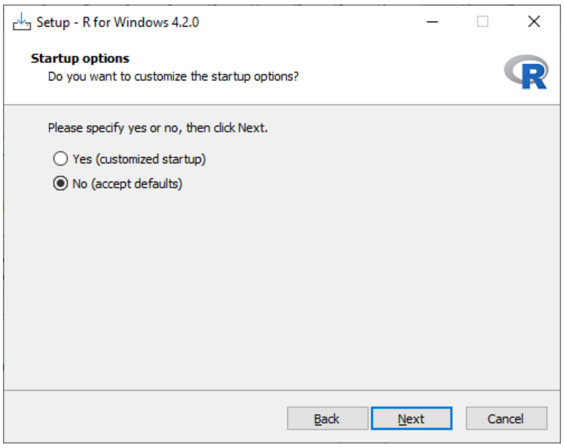

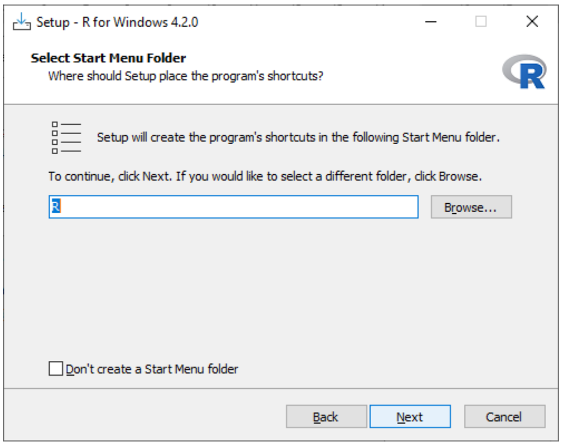

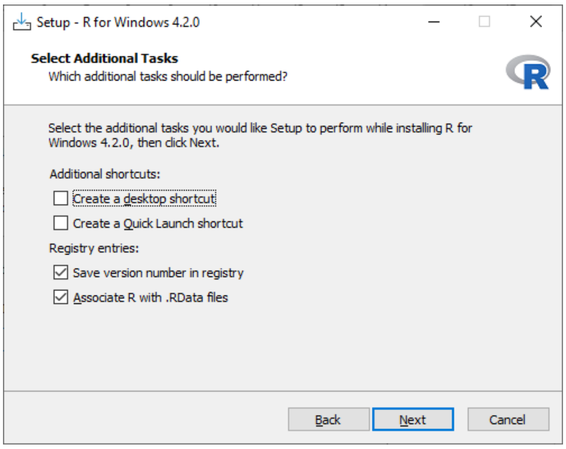

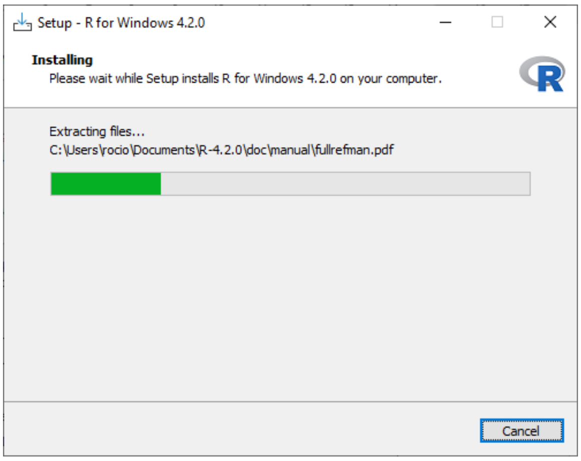

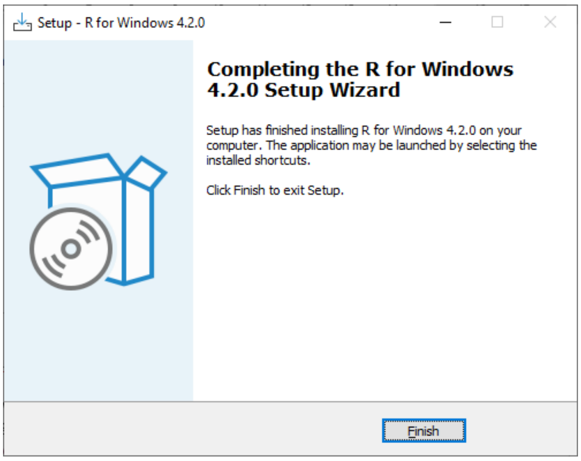

Once R has been installed, the ***All in One Installer*** will start the installation of the third-part software **XAMPP**.

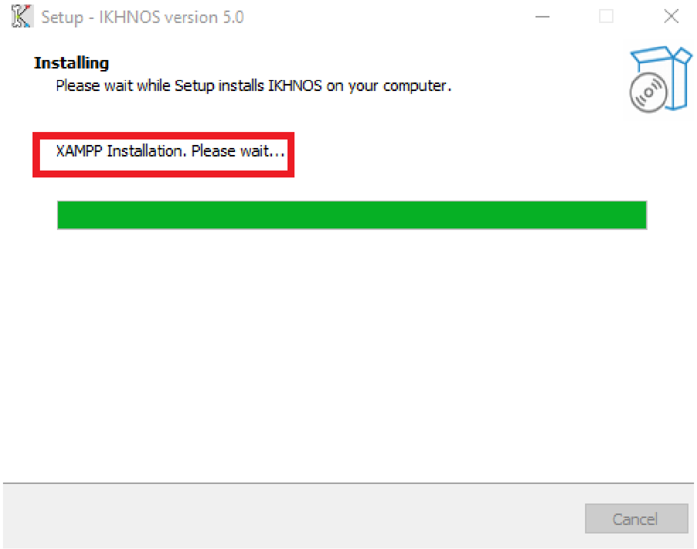

Warning messages will appear if an Antivirus software is running in the user's PC. In that case, users have to click on the **Yes** and **OK** buttons to continue the installation process.

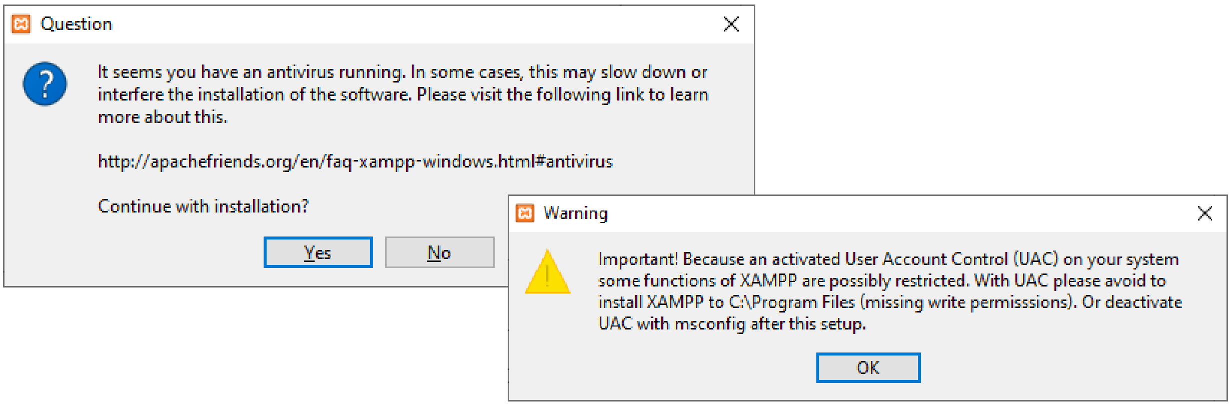

To install XAMPP, it is recommended to keep the **default options**, including the selected installation path **C:\xampp**

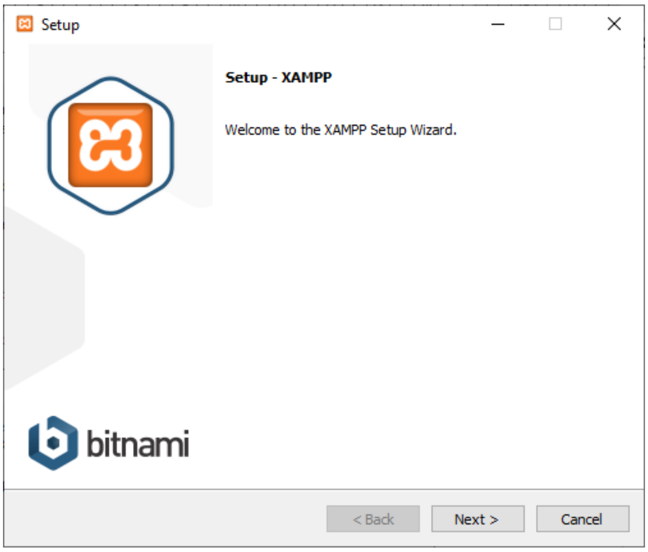

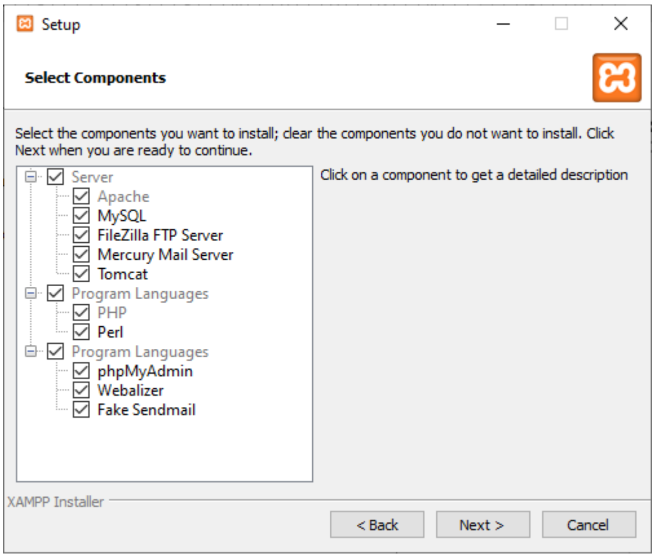

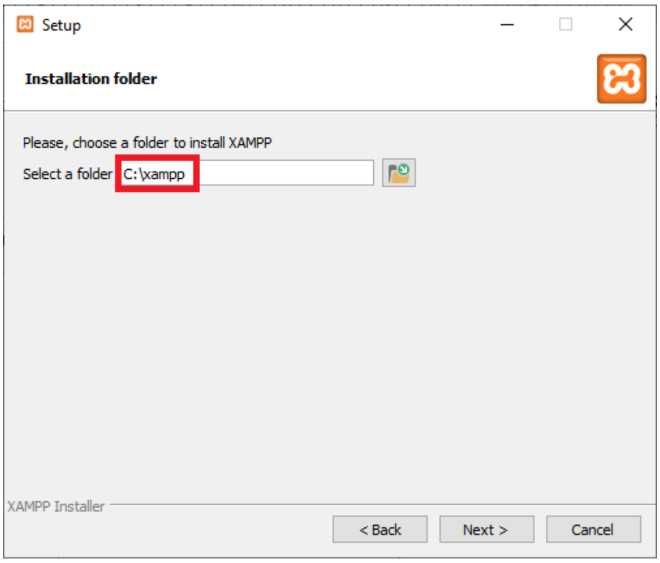

Users can select the **language** before starting the installation process that will begin after clicking on the **Next** button in the three coming windows.

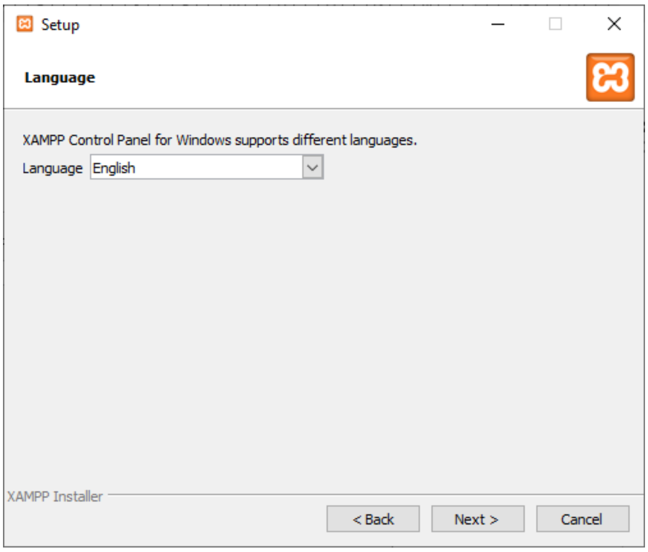

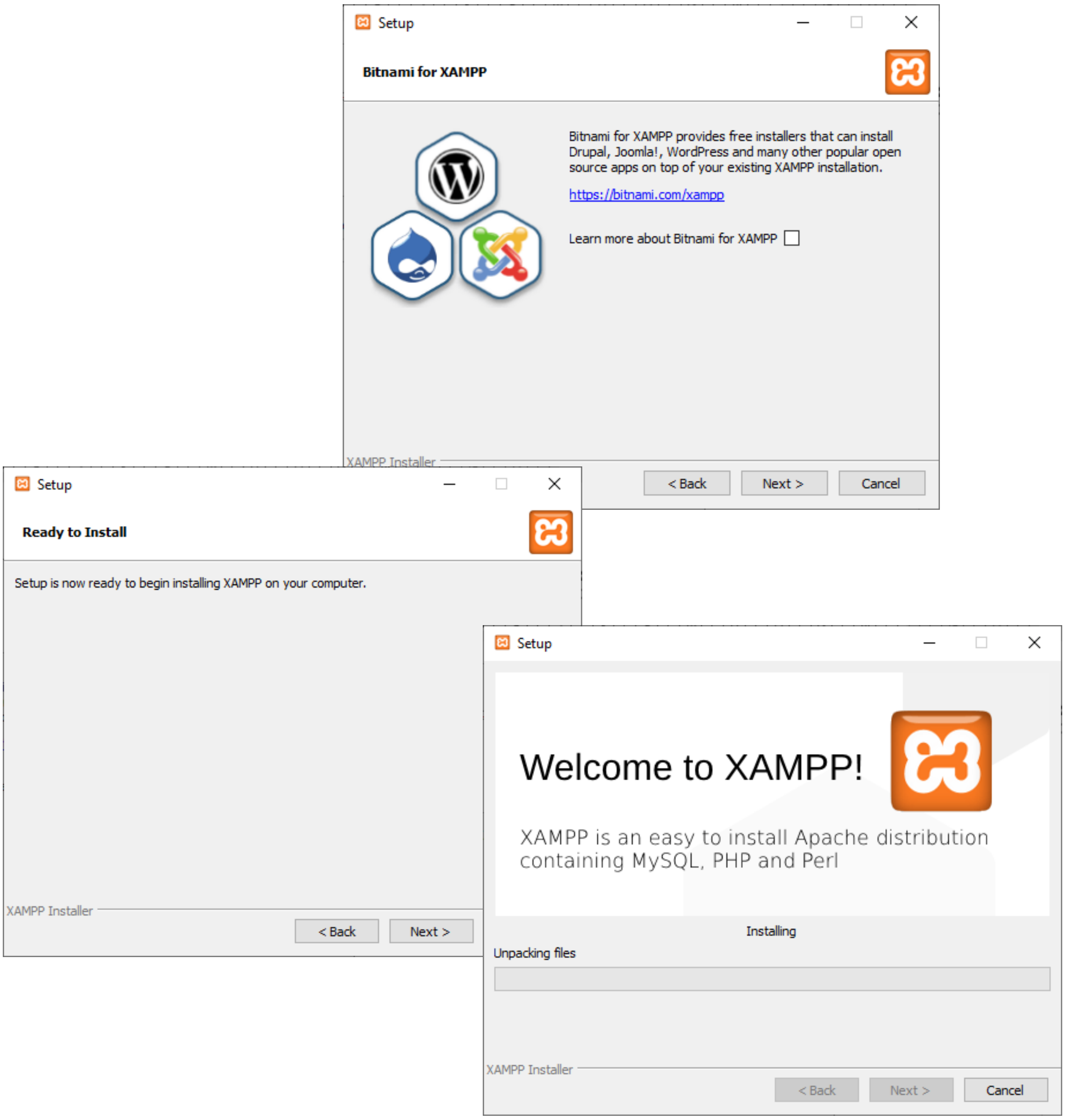

The XAMPP installer will give users the opportunity to **start the XAMPP control panel** after finishing the Setup installation. Users must tick that option and click on the **Finish** button to end the configuration.

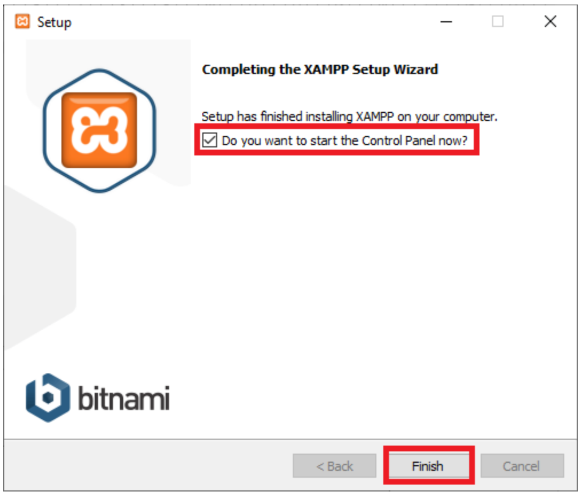

In the **XAMPP Control Panel** the **Apache Module** and the **MySQL Module** have to be **activated (green)** using the **Start/Stop** button.

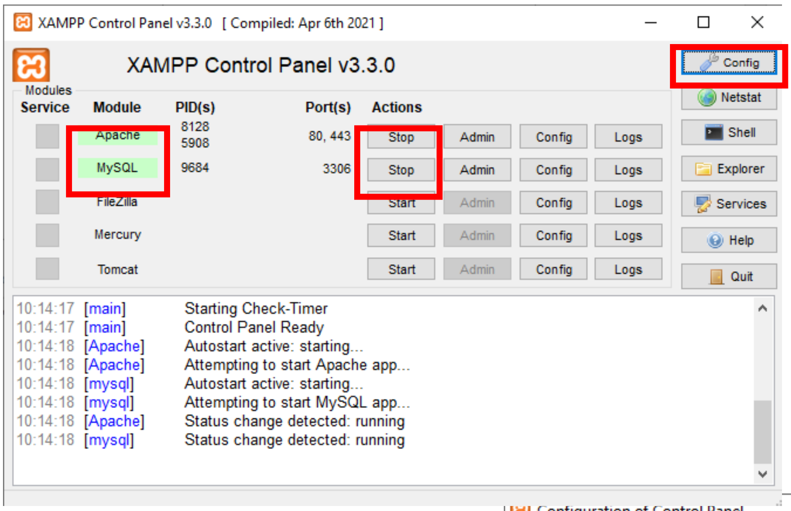

By clicking on the **Config** button (on the top right of the window) and checking the **Auto Start Modules** function for the **Apache** and **MySQL** modules, users can programme their automatic activation. Users have to click on the **Save** button before closing the control panel to save the new configuration.

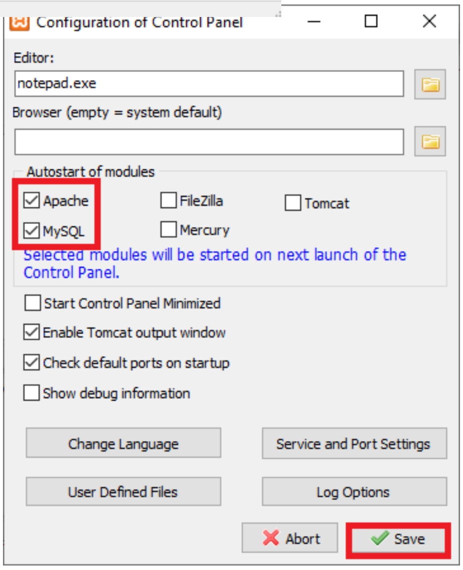

After performing all these steps, the main installation process will end.

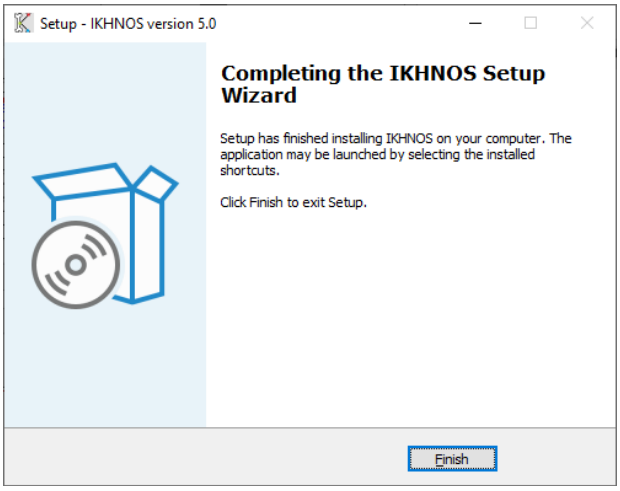

1.2. *Configuration of R and Database*

After installing *IKHNOS* and the third-part programmes, the required R packages and libraries and the database tables need to be installed using the **Database_Setup.bat** file available in the **IKHNOS_5_0_Windows_x64** folder.

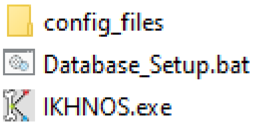

First, click on the **Database_Setup.bat** using the right mouse button to **Run as Administrator.** A **command (cmd) pop-up window** will appear, including different commands. The last line will ask users to **press any key to continue.** Users must press any key **but for the Intro key** to continue the configuration process:

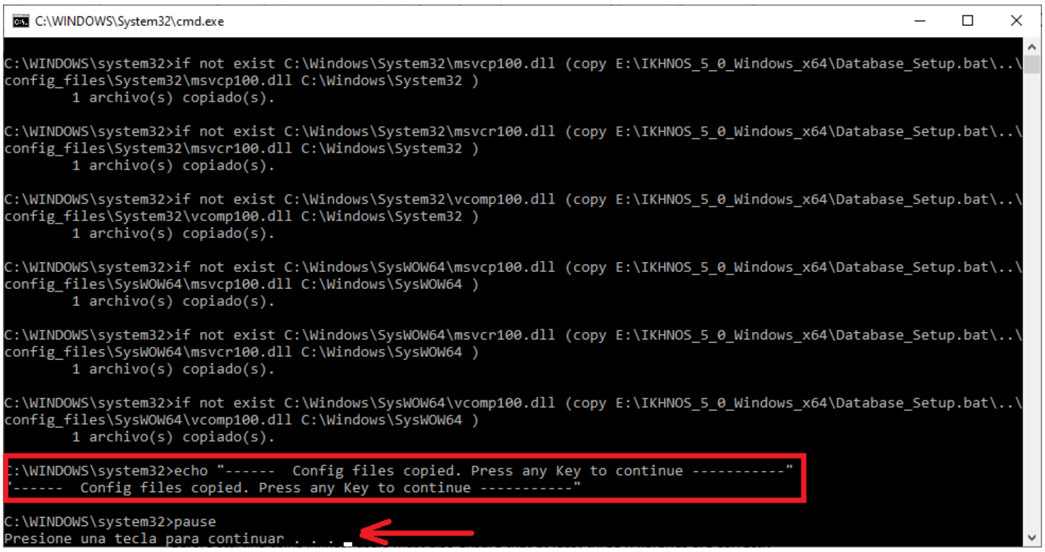

During this process all the necessary R libraries will be downloaded and installed. This process may take a while.

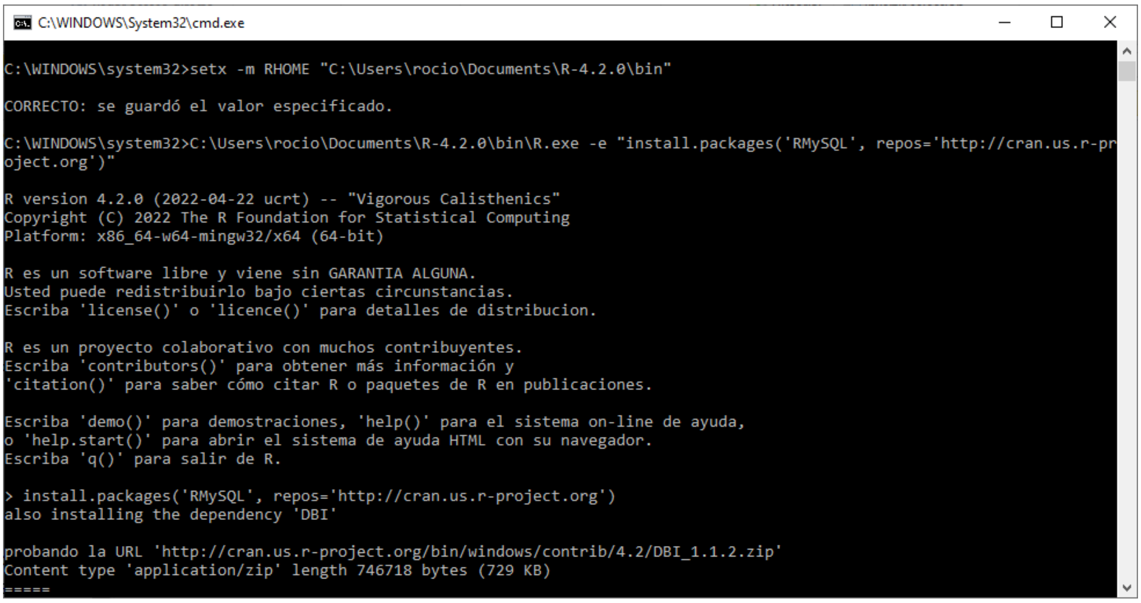

Once finished, a message will ask users to again **press any key to continue** (but for the Intro key)

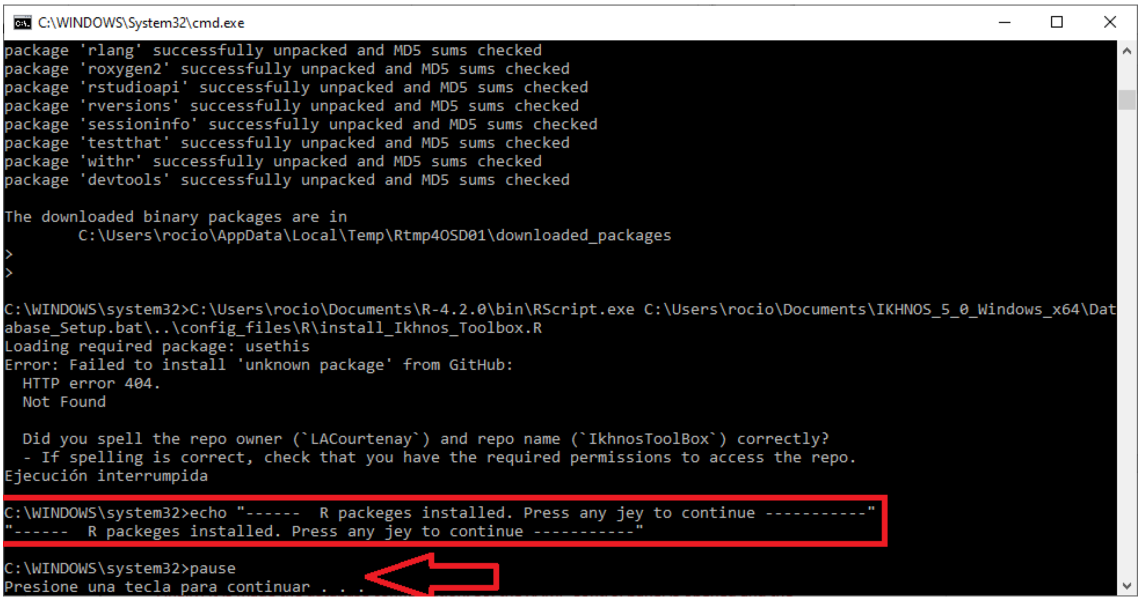

The configuration of the database in XAMPP will begin automatically by opening the control panel, which can be closed, as the database will be directly created under the name **taphonomy**, where data will be stored.

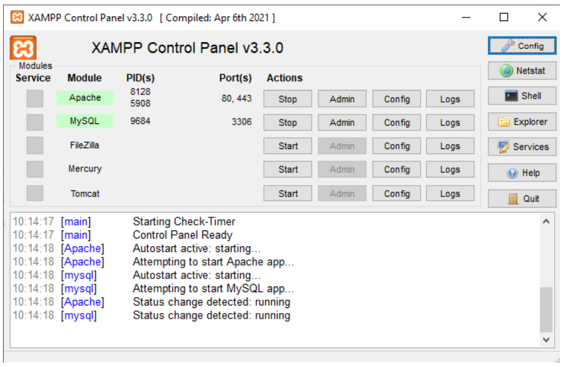

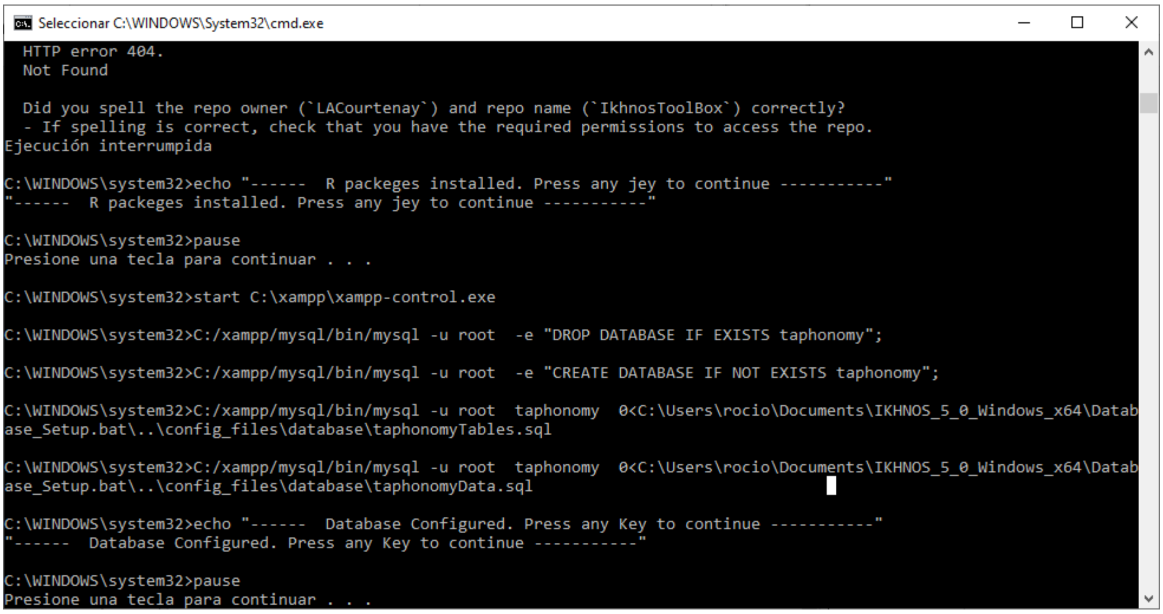

Once the taphonomy database has been created and saved in the database management system developed using MySQL, a message will ask users to **press any key to continue** (but for the Intro key). In doing so, the installation process will finish and users can start using *IKHNOS* and all the integrated tools.

2. *IKHNOS* utilities

Before using *IKHNOS*, users must always open the **XAMPP control panel**, as it creates the link between the *IKHNOS* graphical user interface (GUI) and the database management system.

The *IKHNOS* GUI includes three tabs: Bone 3D Model, Heatmap, Statistics R.

2.1. Documentation and registration

(A) Bone 3D Model: for documentation and edition of bone fragments and BSM

The main window is divided in two modules. One of the modules is devoted to the **interactive 3D viewer of each bone's 3D models** using different formats (i.e., point cloud, mesh model, polygonal model). 3D models of all limb long bones (including the left and right sides) of a horse, a deer and a human are available and serve as template for the documentation of fragmentation patterns and BSM presence and location.

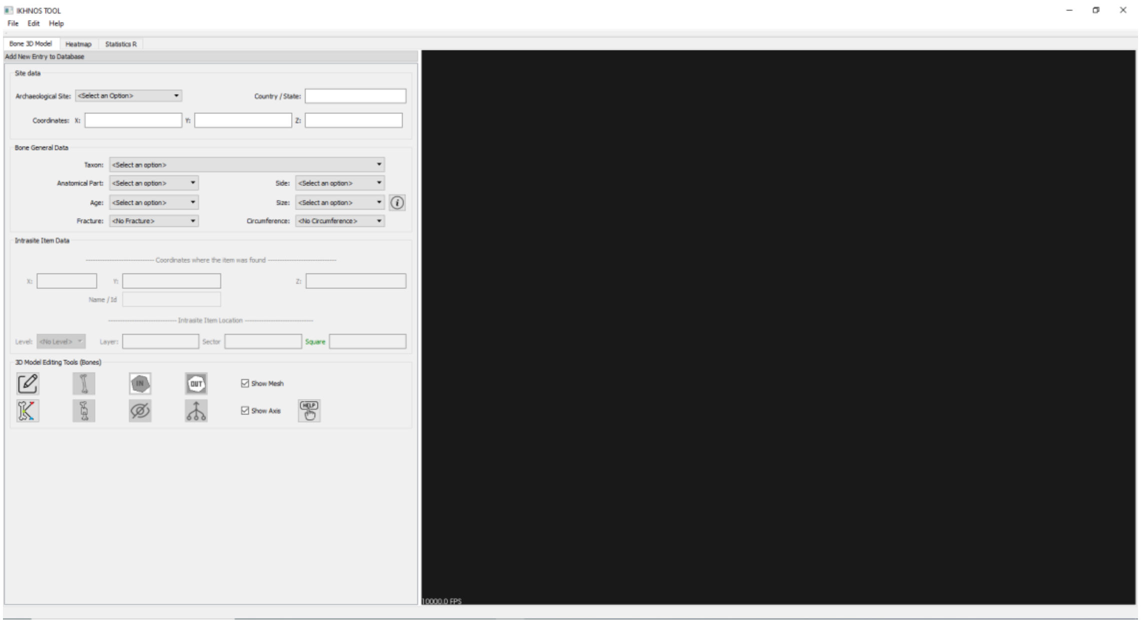

BSM digitisation requires the interaction with the second module in the documentation tab, where four main sections can be distinguished:

Site data: refers to the archaeological site where the item was found. All sites are stored in the database and made visible in a drop-down list. New sites can be manually added by introducing the name of the site and its spatial coordinates.

Bone general data: allows the selection of data regarding taxa (human, equid, cervid, bovid, ovicaprid, suid), anatomical part (humerus, radius, femur, tibia, metacarpus and metatarsus), age (sub-adult, adult), laterality (right, left) and animal size (1 to 6, being 1 and 2 tagged as small size, 3 as medium size, and 4 to 6 as large size, as explained in [1]). Data regarding bone breakage patterns can also be recorded here, including the type of breakage pattern (dry or green) and the shaft completeness according to Bunn's [2] types: (1) <50% of the shaft circumference is preserved; (2) >50% of the shaft circumference is preserved; (3) the complete cylinder is preserved. While *Fracture* and *Circumference* are not mandatory, the rest of the values are required in order to show the bone 3D model.

Intrasite item data: the user can add specific information about the specimen or information regarding the geological and archaeological references, such as the coordinates where it was found, the level etc. If chosen, the user can leave this information empty, with the exception of the item ID, the only mandatory identifier for the database.

3D model editing tools: allows the user to edit the 3D model and to segment the precise regions of interest, so as to represent the preserved bone portions in each case. For that purpose, several icons are available once the *Start Editing Model* is selected. Control specifications are available by clicking on the help button.

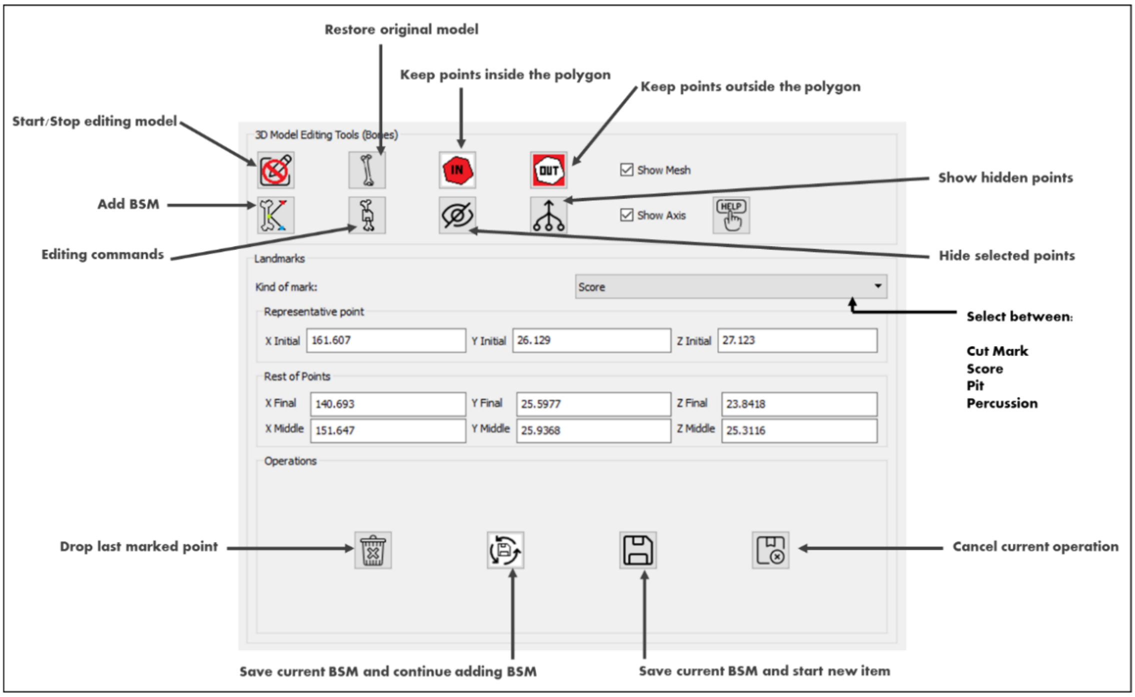

Bone survivorship is documented per specimen through **polygonal cropping** directly on the 3D point cloud representing the selected long bone. Cropped elements will be stored separately in the *CroppedCases* folder.
This section also enables the **digitisation of BSM** by clicking on the precise location of the observation on the 3D model. Two types of landmarks are available; circular marks consisting in a single point which can be assigned for **tooth (blue) or percussion pits**

(black); and linear marks, composed of three points (beginning, middle and end), that can be used to document **tooth scores (green) or cut marks (red)**. The coordinates for each of the landmarks are automatically outlined in the textbox and are stored in the database as a new registered spatial point. As many BSM as desired can be digitised on a single specimen.

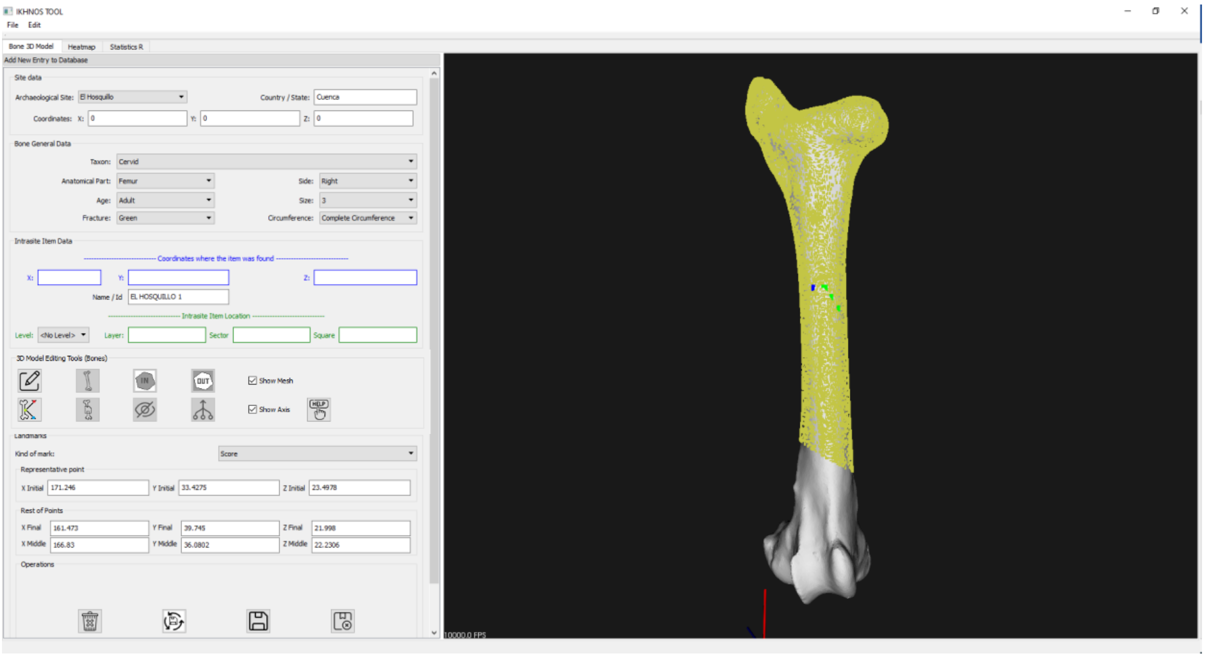

2.2. Bone survivorship

(B) Heatmap: for the extraction on the minimum number of elements (MNE) according to the generation of heatmaps based on bone survivorship.

3D models can be reloaded and superimposed in the *Heatmap* tab by selecting **specific conditions** such as the taxon, the element, or the side and size, among others. This window enables the generation of **colormaps** to visualise bone survivorship and calculate the **minimum number of elements (MNE)**. Heatmaps will consider all cropped elements saved in the *CroppedCases* folder, as well as the **complete specimens** (without cropping), as long as they meet the selected conditions. Heatmaps can be saved in case the user wants to open it in a different software piece.

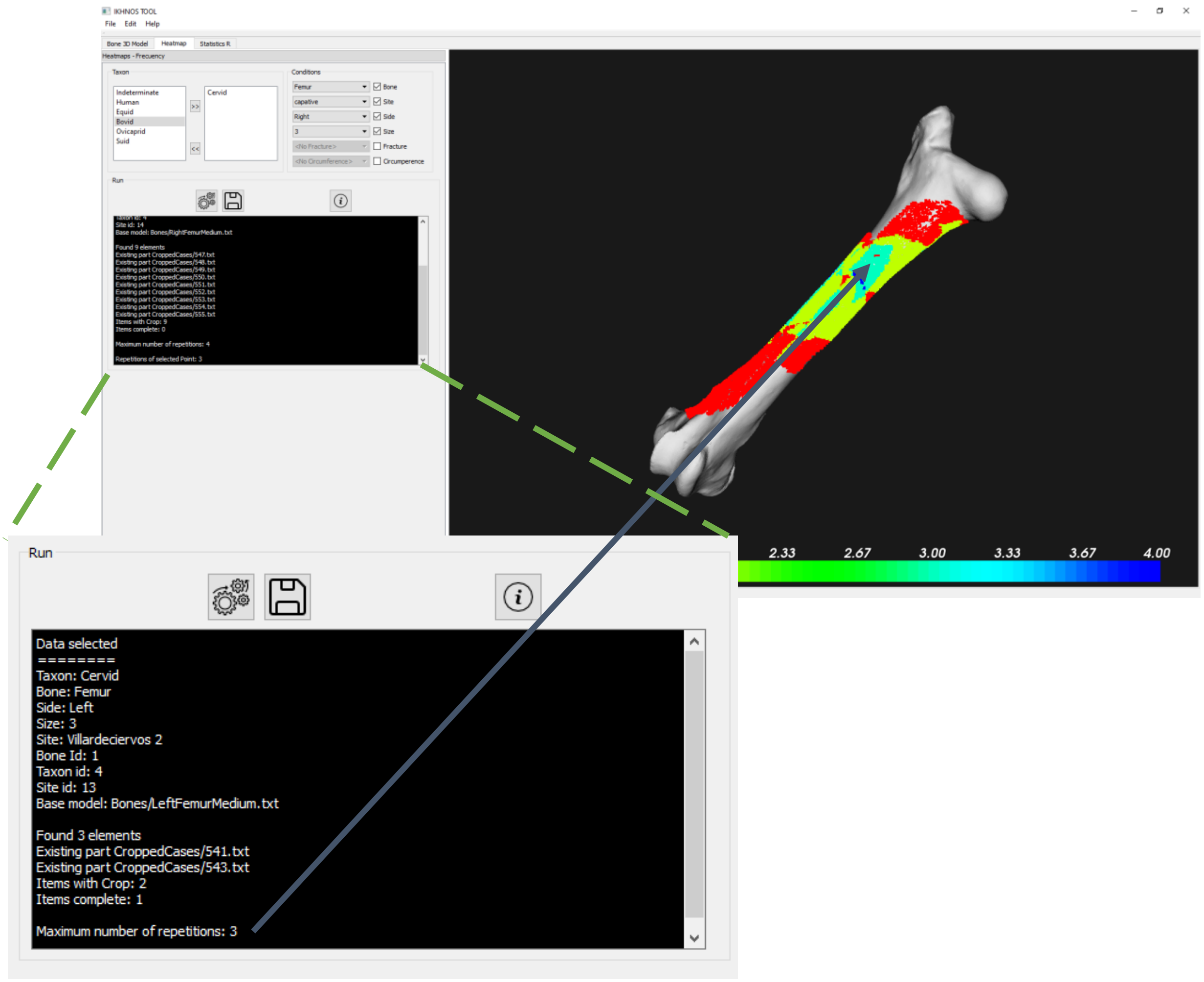

Different **controls** (explained in the ***i***nformation button) are available in this tab:

- u = show/hide scale
- a = show/hide 3D mesh (Warning: if the 3D mesh is visible the scale will not be accurate)
- SHIFT + left click = show individual number of repetitions for each colour

2.3. Statistics

(C) Statistics R: for data selection and statistical analyses.

The *Statistics R* tab includes three modules: one for the **selection of specific data** previously recorded in the documentation tab; a second one for the **selection of the statistical tests** to be performed; and a third module for the **visualisation of the operation reports** and of the numerical results.

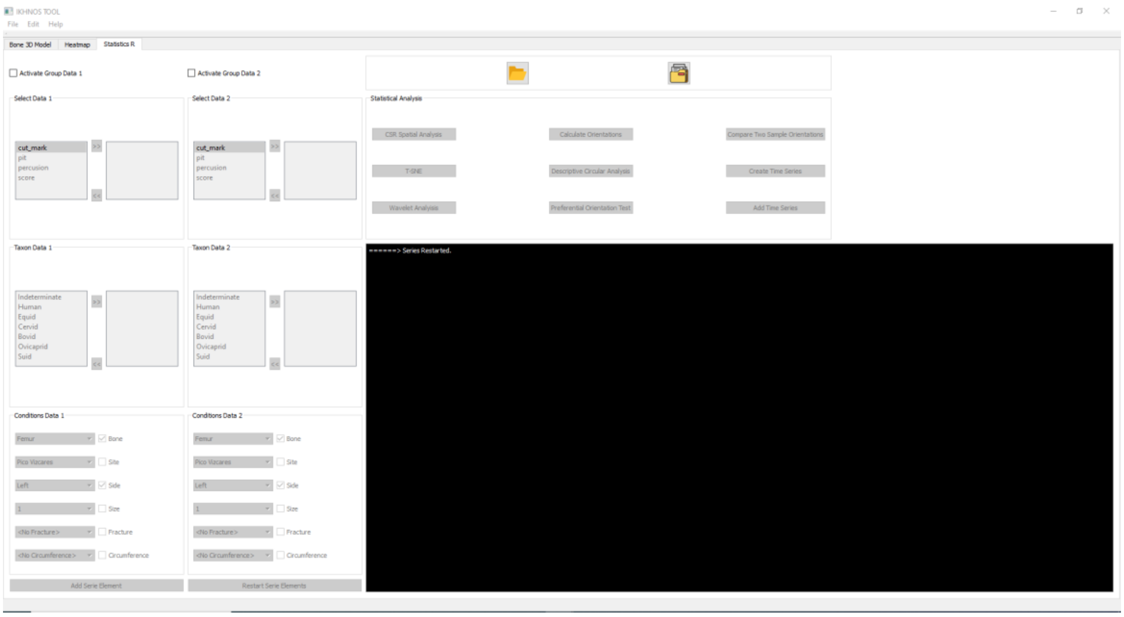

Graphical results are saved in the folders generated and stored in the selected directory after pressing on the ***Set Results Destination Folder*** (1) button available in the second module. Statistical analyses will only be activated once the destination folder is selected (3). Graphical results can be visualised either in the selected directory, or by clicking on the ***Open Results*** (2) button.

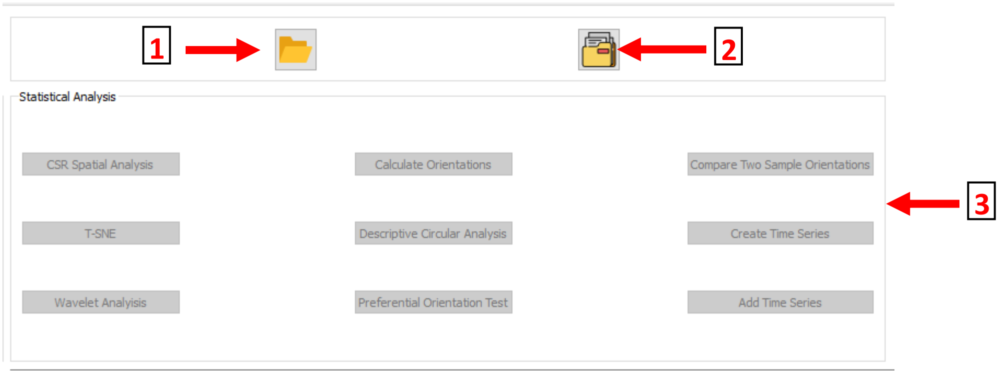

Several options are available for the analysis of individual datasets or the comparison between sets of bones formed for instance by different elements or carcass sizes.

- **Time Series**: visualisation of BSM distribution
- **Complete Spatial Randomness (CSR) analysis** & **Wavelet analysis**: statistical exploration of distribution patterns and comparison
- **t-Distributed Stochastic Neighbour Embedding (t-SNE)**: identification of patterns and clusters through very powerful dimension reduction techniques
- **Calculate Orientations, Descriptive Circular Analysis, Preferential Orientation Tests** & **Compare two sample Orientations**: orientation analyses including calculation, description and comparison of linear marks (i.e., cut marks and scores)

Details and other specifics regarding the statistical repertoire offered by *Ikhnos* are described in the *IkhnosToolBox* documentation (Suppl. File 2).

Statistical tests that require at least two samples:

- T-SNE
- Wavelet Analysis
- Compare Two Sample Orientations

Statistical tests that only accept linear marks:

- Calculate Orientations
- Descriptive Circular Analysis
- Preferential Orientation Test
- Compare Two Sample Orientations

Statistical tests that require multiple samples:

- Create Time Series: It can be used once samples on different elements have been selected using the *Add Serie Element* button.
- Add Time Series: It can be used once additional samples including the same elements included in the Create Time Series have been selected using the *Add Serie Element* button.

WARNING: It is recommended that the *Restart Series Elements* button is pressed before creating and adding data to the Time Series.

After selecting the desired statistical test, a **pop-up window** will appear so that the user can determine certain **parameters** related to the analysis and its graphical design, such as the number of iterations, group labels or titles. The selection of specific data from the 3D geospatial database requires the specification of certain parameters, such as the type of mark, the element, and its side. Other specifications such as site, size, breakage pattern etc. are optional and can be selected upon the user's criteria.

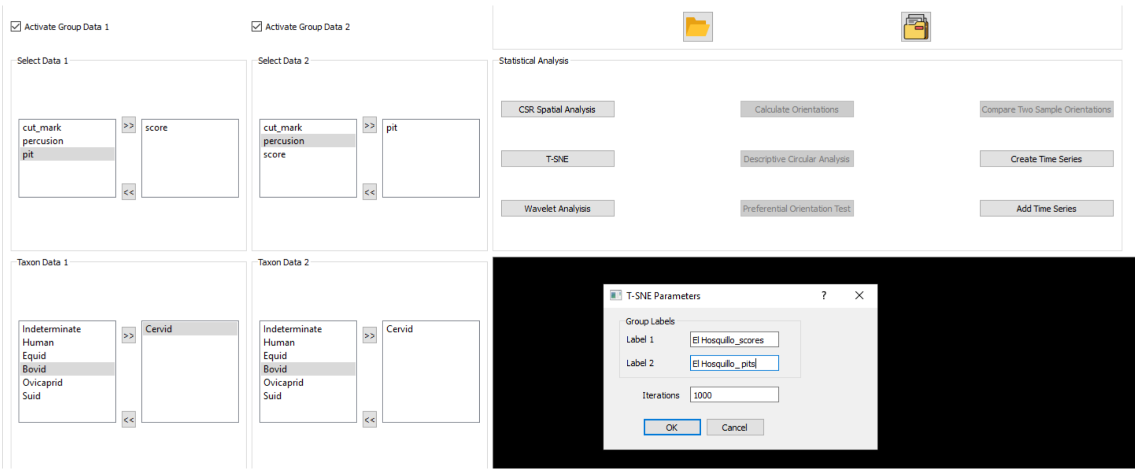

References

1. Bunn, H.T.; Bartram, L.E.; Kroll, L.M. Variability in bone assemblage formation from Hadza hunting, scavenging, and carcass processing. *Journal of Anthropological Archaeology* **1988**, *7*, 412-457.
2. Bunn, H.T. Meat-eating and Human Evolution: Studies on the Diet and Subsistence Patterns of Plio-pleistocene Hominids in East Africa. Ph.D. Dissertation. University of California, Berkeley, 1982.
