# Supplementary material for: Ikhnos: A Novel Software to Register and Analyze Bone Surface Modifications Based on Three-Dimensional Documentation
Source: Animals (Basel). 2022 Oct 20;12(20):2861. doi: 10.3390/ani12202861 (PMC9598086; doi:10.3390/ani12202861)

Ikhnos: a novel software to register and analyse bone surface modifications based on three-dimensional documentation

Supplementary File S2: IkhnosToolBox Documentation

Rocío Mora[1], Julia Aramendi[1,2], Lloyd A. Courtenay[1,3], Diego González-Aguilera[1, *], José Yravedra[3,4], Miguel Ángel Maté-González[1,5], Diego Prieto-Herráez[1,6], José Mª Vázquez-Rodríguez[7], and Isabel Barja[8,9]

[1]Department of Cartographic and Terrain Engineering, Higher Polytechnic School of Ávila, Universidad de Salamanca, Hornos Caleros 50, 05003, Ávila, Spain
[2]Department of Geology, Facultad de Ciencia y Tecnología, Universidad del País Vasco - Euskal Herriko Unibertsitatea (UPV/EHU), Barrio Sarriena s/n, 48940, Leioa, Spain
[3]Department of Prehistory, Ancient History and Archaeology, Universidad Complutense de Madrid, Prof. Aranguren 8 s/n, 28040, Madrid, Spain
[4]C. A. I. Archaeometry and Archaeological Analysis, Universidad Complutense de Madrid, 28040, Madrid, Spain
[5]Department of Topographic and Cartography Engineering, Higher Technical School of Engineers in Topography, Geodesy and Cartography, Universidad Politécnica de Madrid, Mercator 2,28031 Madrid, Spain.
[6]Institute of Fundamental Physics and Mathematics, Universidad de Salamanca, Casas del Parque 1, 05003, Salamanca, Spain
[4]Department of Prehistory and Archaeology, UNED. Humanities Faculty, C/ Senda del Rey, 7, 28040, Madrid, Spain
[8]Department of Biology, Zoology Unit, Universidad Autónoma de Madrid, C/ Darwin 2, Campus Universitario de Cantoblanco, 28049, Madrid, Spain
[9]Center of Investigation in Biodiversity and Global Change (CIBC-UAM), Universidad Autónoma de Madrid, Madrid, Spain.
[*]Corresponding author. Email: daguilera@usal.es

The present document contains a brief documentation of the functions available in the **IkhnosToolBox** R library.

Contents

IkhnosToolBox Software Details 3
Download and Installation 3
Example Datasets 4
Functions 5
load_bone 5
load_marks 6
save_3d_image 8
extract_spatial_data 9
perform_CSR_analyses 10
tsne_calculation 12
two_sample_histogram_distributions 14
wavelet_analysis 16
calculate_orientations 18

descriptive_circular_analysis 19
preferential_orientation_test 20
compare_two_sample_orientations 21
create_time_series 22
add_time_series 24
Example Code **26**

IkhnosToolBox Software Details

Authors	Lloyd Austin Courtenay [Author, Creator]<br>Julia Aramendi [Author]
Version	1.0.0
Date	16-05-2022
Title	R Tool Box for the Ikhnos Taphonomic Software
R Version	R (>= 2.10)
Dependencies	rgl (>= 0.107), plot3Drgl (>= 1.0), spatstat (>= 2.0), spatstat.core (>= 2.0), spatstat.geom(>= 2.0), biwavelet (>= 0.20), tidyverse (>= 1.3), ggplot2 (>= 3.0), RMySQL (>= 0.10), Rtsne (>= 0.15), Rcpp (>= 1.0), circular (>= 0.4), abind (>= 1.4), factoextra (>= 1.0), gridExtra (>= 2.3), alphahull(>= 2.2), RColorBrewer (>= 1.1), fpc (>= 2.2), sm (>= 2.2)
Suggests	FactoMineR (>= 2.4)
Description	Group of R functions for the statistical analysis of data derived from the Ikhnos taphonomic software. Functions in this package allow one to visualise the location and orientation of bone surface modifications such as tooth and percussion pits, cut marks and tooth scores on long bones and their analysis for the identification and comparison of orientation and distribution patterns along the bone surface. Functions are available to perform analyses within samples as well as across different samples.
License	MIT License, Copyright ©2021 Lloyd Austin David Courtenay

Download and Installation

The *IkhnosToolBox* library is available as a GitHub repository from the corresponding author's GitHub page. This can be found via the following link; https://github.com/TIDOP-USAL/IkhnosToolBox.

To install an R library from a GitHub repository the user must first have installed *devtools*. For those unfamiliar with this package, the *devtools* package is a set of tools used for the development and management of R packages. This package can simply be installed using;

```
install.packages("devtools")
```

Once *devtools* has been installed, we can use the *install_github* function to access the *IkhnosToolBox* repository and install the library.

```
library(devtools)
install_github("TIDOP-USAL/IkhnosToolBox")
```

Once installed, we can access the *IkhnosToolBox* library at any point by simply including the line

```
library(IkhnosToolBox)
```

in our code.

Example Datasets

The *IkhnosToolBox* library contains 18 datasets and 16 bone point clouds that correspond to the right and left long bones of a cervid and a bovid.

Dataset	Nº	Mark Type	Element	Side	Size
femur_right_circular1	123	Pit	Femur	Right	Large
femur_right_circular2	130	Pit	Femur	Right	Medium
femur_right_circular3	65	Pit	Femur	Right	Small
femur_right_linear1	87	Score	Femur	Right	Large & Medium
femur_right_linear2	99	Score	Femur	Right	Large & Medium
humerus_right_circular1	155	Pit	Humerus	Right	Large
humerus_right_circular2	150	Pit	Humerus	Right	Medium
humerus_right_circular3	37	Pit	Humerus	Right	Small
metacarpus_right_circular1	26	Pit	Metacarpus	Right	Large
metatarsus_right_circular1	86	Pit	Metatarsus	Right	Large
metatarsus_right_circular2	21	Pit	Metatarsus	Right	Medium
metatarsus_right_circular3	123	Pit	Metatarsus	Right	Small
radius_right_circular1	70	Pit	Radius	Right	Large
radius_right_circular2	119	Pit	Radius	Right	Medium
radius_right_circular3	134	Pit	Radius	Right	Small
tibia_right_circular1	154	Pit	Tibia	Right	Large
tibia_right_circular2	134	Pit	Tibia	Right	Medium
tibia_right_circular3	81	Pit	Tibia	Right	Small

Author

Julia Aramendi

See Also

right_femur, left_femur, right_humerus, left_humerus, right_metacarpus, left_metacarpus, right_radius, left_radius, right_tibia, left_tibia, right_metacarpus, left_metacarpus, for point cloud dataset

Functions

load_bone	*Load bone for visualisation*

Description

The present function creates a 3D popup window visualising the bone under study. This can then be combined with functions such as *load_marks* to visualise marks on the 3D model, and *save_3d_image* to save the window to a .png file.

Usage

```
load_bone <- function(bone_data)
```

Arguments

bone_data	An IkhnosToolBox data object (e.g., data(left_femur)) containing the 3D model of the bone being studied.

Details

This function returns a 3D popup window with the selected bone mesh points.

See Also

load_marks, save_3d_image, plot3d, aspect3d

Example

```
data("right_femur")
data("femur_right_circular1")
data("femur_right_linear1")
load_bone(right_femur)
load_marks(
    femur_right_circular1,
    mark_type = "circular",
    plot = TRUE,
    colour_value = "black"
)
load_marks(
    femur_right_linear1,
    mark_type = "linear",
    plot = TRUE,
    colour_value = "red"
)
save_3d_image("my_first_plot")
```

load_marks	*Load marks from Ikhnos files*

Description

The present function allows the user to locate a file and load data derived from *Ikhnos* for both visualisation and preparation for statistical applications. If the *load_bone* function has already been used, then marks are visualised directly on the surface of the bone that is currently in view. If no *load_bone* function has been called, then the plot will contain only the 3D distribution of marks. This can then be combined with functions such as *load_marks* to visualise marks on the 3D model, and *save_3d_image* to save the window to a .png file.

Usage

```
load_marks <- function(
    data,
    file_name = NULL,
    mark_type = "circular",
    delim = ",",
    plot = FALSE,
    colour_value = "black",
    mark_size = 6
)
```

Arguments

data	A data frame loaded externally from the library, using either data() or the user's own data.
file_name	A string containing the path of the Ikhnos product file. If no file name is specified, then a popup window will appear allowing the user to locate the file in their system.
mark_type	A lower case string specifying whether the marks under study are "circular" or "linear".
plot	A boolean TRUE or FALSE option (default = FALSE) defining whether to produce a 3D plot of the marks or not.
delim	A string specifying how marks are delimited within the Ikhnos product file.
colour_value	A lower case string specifying the colour of the marks in the case where plot = TRUE.
mark_size	The size of the points or lines produced in the 3D plot.

Details

The present function returns a data frame object containing the data extracted from Ikhnos. If plot = TRUE, then a 3D visualisation of marks is also created.

See Also

load_marks, save_3d_image, plot3d, aspect3d

Example

```
data("right_femur")
data("femur_right_circular1")
data("femur_right_linear1")
load_bone(right_femur)
```

```
load_marks(
    femur_right_circular1,
    mark_type = "circular",
    plot = TRUE,
    colour_value = "black"
)
load_marks(
    femur_right_linear1,
    mark_type = "linear",
    plot = TRUE,
    colour_value = "red"
)
save_3d_image("my_first_plot")
```

save_3d_image *Save 3D visualisation*

Description

Function to save visualisation of data.

Usage

```
save_3d_image <- function(file_name)
```

Arguments

file_name	A string specifying the file name where the .png image will be saved.

Details

This function saves the visualised 3D window as a .png file.

See Also

load_bone, save_3d_image, rgl.snapshot

Example

```
data("right_femur")
data("femur_right_circular1")
data("femur_right_linear1")
load_bone(right_femur)
load_marks(
    femur_right_circular1,
    mark_type = "circular",
    plot = TRUE,
    colour_value = "black"
)
load_marks(
    femur_right_linear1,
    mark_type = "linear",
    plot = TRUE,
    colour_value = "red"
)
save_3d_image("my_first_plot")
```

Description

The present function is used to prepare data for further spatial analysis.

Usage

```
extract_spatial_data <- function(
    input_data,
    mark_type = "circular",
    print_summary = TRUE,
    plot_results = FALSE,
    create_external_plot = FALSE
)
```

Arguments

input_data	A data frame containing spatial coordinates.
mark_type	A string specifying whether marks are "circular" or "linear".
print_summary	A boolean TRUE or FALSE option (default = TRUE) to print a summary of the data included in the spatial object.
plot_results	A boolean TRUE or FALSE option (default = FALSE) to plot the spatial information.
create_external_plot	A boolean TRUE or FALSE (default = FALSE) option to create a popup window with the plot for the mark frequency in the sample.

Details

The present function returns a spatial object for statistical analyses.

Author

Lloyd A. Courtenay

See Also

box3, pp3

Example

```
data("femur_right_circular1")
example_data <- load_marks(femur_right_circular1, mark_type = "circular")
example_sp_object <- extract_spatial_data( example_data, "circular")
```

Description

The present function conducts spatial analyses on the 3D point patterns representing the marks using the *K3est*, *F3est*, *G3est* and *pcf3est* functions that, respectively, estimate the K-function, the empty space function, the nearest-neighbour distance distribution function, and the pair correlation function from a 3D point pattern. Square root versions of the *K3est*, *F3est* and *G3est* functions are included to stabilise variance if needed.

Usage

```
perform_CSR_analyses <- function(
    spatial_object,
    n_permutations = 1000,
    create_external_plot = FALSE
)
```

Arguments

spatial_object	A pp3 object containing the 3D point pattern.
n_permutations	A positive, non-zero integer to define the number of permutations (default = 1000, min n = 101).
create_external_plot	A boolean TRUE or FALSE option (default = FALSE) to create three popup windows with the plots for the four functions and their respective square root versions.

Details

The present function returns plots for the output of the *K3est*, *F3est*, *G3est* and *pcf3est* functions, and for the square root versions of the *K3est*, *F3est* and *G3est* functions.

See Also

spatstat.core, K3est, F3est, G3est, pcf3est

Notes

Functions assume that point distribution is homogeneous and could therefore inflate the identification of clustering patterns when inhomogeneous distributions are present. Functions for inhomogeneous patterns are not available for 3D point patterns yet.

Bibliography

Baddeley, A.J, Moyeed, R.A., Howard, C.V. and Boyde, A. (1993) Analysis of a three-dimensional point pattern with replication. Applied Statistics 42, 641-668.

Baddeley, A.J. and Gill, R.D. (1997) Kaplan-Meier estimators of interpoint distance distributions for spatial point processes. Annals of Statistics 25, 263-292.

Borgefors, G. (1986) Distance transformations in digital images. Computer Vision, Graphics and Image Processing 34, 344-371.

Chiu, S.N. and Stoyan, D. (1998) Estimators of distance distributions for spatial patterns. Statistica Neerlandica 52, 239-246.

Hanisch, K.-H. (1984) Some remarks on estimators of the distribution function of nearest neighbour distance in stationary spatial point patterns. Mathematische Operationsforschung und Statistik, series Statistics 15, 409-412.

Ohser, J. (1983) On estimators for the reduced second moment measure of point processes. Mathematische Operationsforschung und Statistik, series Statistics, 14, 63-71.

Ripley, B.D. (1977) Modelling spatial patterns (with discussion). Journal of the Royal Statistical Society, Series B, 39, 172-212.

Example

```

data("femur_right_circular1")
example_data <- load_marks(femur_right_circular1, mark_type = "circular")
example_sp_object <- extract_spatial_data(example_data, "circular")
example_CSR_analyses <- perform_CSR_analyses(
    example_sp_object, n_permutations = 101
)
```

tsne_calculation	Calculate a t-Distributed Stochastic Neighbor Embedding (t-SNE)

Description

The present function provides a data exploration and visualisation technique based on a non-linear dimensionality reduction algorithm that constructs a low dimensional embedding of high-dimensional data for the identification of patterns and trends in the data. An additional non-supervised machine learning algorithm (DBSCAN) is used in this function to detect patterns in the new feature space, which can be used to define groups of marks.

Usage

```
tsne_calculation <- function(
    data,
    bone,
    labels = NULL,
    n_iterations = 1000,
    perplexity = NULL,
    cluster_eps = 3,
    plot_colours = NULL,
    point_size = 2,
    create_external_plot = FALSE
)
```

Arguments

data	An array containing the 3D points.
bone	A 3D model of the bone used for analyses provided by data().
labels	A factor containing the group association for each dataset.
n_iterations	A positive, non-zero integer to define the number of permutations for optimisation (default = 1000).
perplexity	A positive, non-zero integer to define the optimal number of neighbours. Larger datasets require a larger perplexity, typical values are between 5 and 50. By default the optimal parameter is calculated for each dataset.
cluster_eps	A positive, non-zero numeric value used to define the eps parameter in the DBSCAN clustering algorithm. If no value is provided, 3 is used as default.
plot_colours	A character vector to assign a specific colour to each label.
point_size	A non-negative integer to define the size of the points in the plot.
create_external_plot	A boolean TRUE or FALSE (default = FALSE) option to generate an external window for the t-SNE plot.

Details

The present function returns a data frame object containing the x and y coordinates, and the associated group, as well as two plots for point pattern visualisation. The first graph illustrates the point pattern trends observed in both groups by group association, while the second one shows the clustering of the point patterns calculated by the DBSCAN algorithm. The assigned colours in the second graph correspond to those observed in the 3D popup window where the selected bone mesh points and the registered marks can be visualised.

Author

Lloyd A. Courtenay

See Also

Rtsne, fpc

Bibliography

Hinton, G.E.; Roweis, S.T. (2003) Stochastic Neighbor Embedding, Advances in Neural Information Processing Systems. 857-864.

Esther, M.; Kriegel, H.P.; Sander, J.; Xu, X. (1996) A density based algorithm for discovering clusters in large spatial databases with noise. In: Proceedings of the 2nd International Conference on Knowledge Discovery and Data Mining, München, Germany. https://www.aaai.org/Papers/KDD/1996/KDD96-037.pdf

Notes

Due to the stochastic nature of the tSNE algorithm, the authors recommend executing this function a number of times to obtain the most reliable overview of the results.

Example

```
data("femur_right_circular1")
data("femur_right_linear1")
data("right_femur")
example_data1 <- load_marks(femur_right_circular1, mark_type = "circular")
example_data2 <- load_marks(femur_right_linear1, mark_type = "linear")
example_sp_object1 <- extract_spatial_data(example_data1, "circular")
example_sp_object2 <- extract_spatial_data(example_data2, "circular")
sample1_coords <- as.matrix(example_sp_object1)
sample2_coords <- as.matrix(example_sp_object2)
sample1_sample2 <- rbind(sample1_coords, sample2_coords)
group_labels <- as.factor(c(
    rep("circular", nrow(sample1_coords)),
    rep("linear", nrow(sample2_coords))
))
tsne_calculation(sample1_sample2, group_labels, bone = right_femur)
```

two_sample_histogram_distributions   *Create pair histograms*

Description

The present function is used to compare the frequency of marks along a specific bone axis recorded in two different samples.

Usage

```
two_sample_histogram_distributions <- function(
    group_1,
    group_2,
    sample_1_name,
    sample_2_name,
    dimension,
    bone_type,
    create_external_plot = FALSE
)
```

Arguments

group_1	A pp3 object containing a 3D point pattern.
group_2	A pp3 object containing a second 3D point pattern.
sample_1_name	A string to define the label of *group_1*.
sample_2_name	A string to define the label of *group_2*.
dimension	A character to define the axis; "x" refers to the proximodistal axis, "y" to the mediolateral axis, and "z" to the craniocaudal axis.
bone_type	A string to define the element; possible bones are "femur", "humerus", "metacarpus", "radius", "tibia, and "metatarsus".
create_external_plot	A boolean TRUE or FALSE option (default = FALSE) to create a popup window with the plot for the distribution of both samples.

Details

The present function returns a list containing the number of breaks and counts for both samples, and a plot for the visual comparison of the mark frequencies along the selected axis.

Author

Lloyd A. Courtenay

See Also

wavelet_analysis, sm.density.compare

Bibliography

Bowman, A.W. and Azzalini, A. (1997). Applied Smoothing Techniques for Data Analysis: the Kernel Approach with S-Plus Illustrations. Oxford University Press, Oxford.

Example

```
data("femur_right_circular1")
data("femur_right_linear1")
```

```
example_data1 <- load_marks(femur_right_circular1, mark_type = "circular")
example_data2 <- load_marks(femur_right_linear1, mark_type = "linear")
example_sp_object1 <- extract_spatial_data(example_data1, "circular")
example_sp_object2 <- extract_spatial_data(example_data2, "circular")
example_time_series <- two_sample_histogram_distributions(
    example_sp_object1,
    example_sp_object2,
    "circular",
    "linear",
    "x",
    "femur"
)
```

wavelet_analysis	*Bivariate wavelet analyses in frequency domains.*

Description

The present function is used to conduct wavelet coherence analyses to assess the dependence between two mark patterns.

Usage

```
wavelet_analysis <- function (
    first_ts,
    second_ts,
    sample_1_name,
    sample_2_name,
    n_iterations = 10000,
    x_lab = "Inter-epiphysis distance",
    create_external_plot = FALSE
)
```

Arguments

first_ts	A matrix (n rows x 2 columns) containing the time series of the first sample. The first column contains the time steps (breaks) and the second column contains the values (counts).
second_ts	A matrix (n rows x 2 columns) containing the time series of the second sample. The first column contains the time steps (breaks) and the second column contains the values (counts).
sample_1_name	A string to define the label that corresponds to *first_ts*.
sample_2_name	A string to define the label that corresponds to *second_ts*.
n_iterations	A positive, non-zero integer to define the number of Monte Carlo randomisation (default = 10000).
x_lab	A string to define the title of the x axis. It should coincide with the dimension selected for the two_sample_histogram_distributions function (e.g., proximal-distal, medial-lateral, cranio-caudal)
create_external_plot	A boolean TRUE or FALSE (default = FALSE) option to create a popup window with the plot for the wavelet coherence analysis.

Details

The present function returns a biwavelet plot to visualise the dependence between two samples.

See Also

wtc, plot.biwavelet, two_sample_histogram_distributions

Bibliography

Cazelles, B., M. Chavez, D. Berteaux, F. Menard, J. O. Vik, S. Jenouvrier, and N. C. Stenseth. 2008. Wavelet analysis of ecological time series. Oecologia 156:287-304.

Grinsted, A., J. C. Moore, and S. Jevrejeva. 2004. Application of the cross wavelet transform and wavelet coherence to geophysical time series. Nonlinear Processes in Geophysics 11:561-566.

Liu, Y., X. San Liang, and R. H. Weisberg. 2007. Rectification of the Bias in the Wavelet Power Spectrum. Journal of Atmospheric and Oceanic Technology 24:2093-2102.

Torrence, C., and G. P. Compo. 1998. A Practical Guide to Wavelet Analysis. Bulletin of the American Meteorological Society 79:61-78.

Torrence, C., and P. J. Webster. 1998. The annual cycle of persistence in the El Nino/Southern Oscillation. Quarterly Journal of the Royal Meteorological Society 124:1985-2004.

Veleda, D., R. Montagne, and M. Araujo. 2012. Cross-Wavelet Bias Corrected by Normalizing Scales. Journal of Atmospheric and Oceanic Technology 29:1401-1408.

Example

```

data("femur_right_circular1")
data("femur_right_linear1")
example_data1 <- load_marks(femur_right_circular1, mark_type = "circular")
example_data2 <- load_marks(femur_right_linear1, mark_type = "linear")
example_sp_object1 <- extract_spatial_data(example_data1, "circular")
example_sp_object2 <- extract_spatial_data(example_data2, "circular")
example_time_series <- two_sample_histogram_distributions(
    example_sp_object1,
    example_sp_object2,
    "circular",
    "linear",
    "x",
    "femur"
)
example_wavelet_analysis <- wavelet_analysis(
    example_time_series$first_sample_ts,
    example_time_series$second_sample_ts,
    "circular",
    "linear"
)
```

calculate_orientations	*Calculation of orientation of linear marks*

Description

Function to conduct orientation analyses on scores and/or cut marks.

Usage

```
calculate_orientations <- function(
    linear_data,
    bone,
    create_external_plot = FALSE
)
```

Arguments

linear_data	A string containing the path of the Ikhnos product file containing linear data (e.g., scores or cut marks). If no file name is specified, then a popup window will appear allowing the user to locate the file in their system.
bone	An IkhnosToolBox data object (e.g., data(left_femur)) containing the 3d model of the bone being studied.
create_external_plot	A boolean TRUE or FALSE (default = FALSE) option to create two popup windows; one for the visualisation of the location of the linear marks on the specified bone; and a second one with the rose diagram based on the orientations of the marks.

Details

The present function returns a circular object containing the angles calculated between the bone long axis and each linear mark. A rose diagram with the output of the calculated mark orientations, and a graph to visualise the location and orientation of the linear marks on the specified bone.

Author

Lloyd A. Courtenay

See Also

load_marks, ashape, circular, arrows.circular

Bibliography

Edelsbrunner, H., Kirkpatrick, D.G. and Seidel, R. (1983). On the shape of a set of points in the plane. IEEE Transactions on Information Theory, 29(4), pp.551-559.

Jammalamadaka, S. Rao and SenGupta, A. (2001). Topics in Circular Statistics, World Scientific Press, Singapore.

Example

```

data("right_femur")
data("femur_right_linear1")
data("femur_right_linear2")
example_circ_1 <- load_marks(femur_right_linear1, mark_type = "linear")
example_circ_2 <- load_marks(femur_right_linear2, mark_type = "linear")
example_calculate_orientations_1 <- calculate_orientations(example_circ_1, right_femur)
example_calculate_orientations_2 <- calculate_orientations(example_circ_2, right_femur)
```

descriptive_circular_analysis *Descriptive circular analysis*

Description

The present function contains several parameters to characterise the location and variability of linear marks along the bone, including normality distribution tests such as skewness (measure of asymmetry) and kurtosis (measure of tailedness and peakedness), circular variance and dispersion, and the orientation based on the calculation of radians and degrees.

Usage

```
descriptive_circular_analysis <- function(circular_object)
```

Arguments

circular_object	A circular object containing the angles calculated between the bone long axis and each linear mark.

Details

The present function returns a list with the results obtained for the standarised measures of Skewness and Kurtosis, the sample circular variance and dispersion, and the central orientation radians and degrees.

Author

Lloyd A. Courtenay

See Also

calculate_orientations, circular, trigonometric.moment

Bibliography

Jammalamadaka, S. Rao and SenGupta, A. (2001). Topics in Circular Statistics, World Scientific Press, Singapore.

Example

```
data("right_femur")
data("femur_right_linear1")
data("femur_right_linear2")
example_circ_1 <- load_marks(femur_right_linear1, mark_type = "linear")
example_circ_2 <- load_marks(femur_right_linear2, mark_type = "linear")
example_calculate_orientations_1 <- calculate_orientations(example_circ_1, right_femur)
example_calculate_orientations_2 <- calculate_orientations(example_circ_2, right_femur)
example_circ_analysis1 <- descriptive_circular_analysis(example_calculate_orientations_1)
example_circ_analysis2 <- descriptive_circular_analysis(example_calculate_orientations_2)
```

preferential_orientation_test	*Uniformity test for the orientation of linear marks*

Description

Function to conduct orientation analyses on scores and/or cut marks using the Rayleigh distribution test.

Usage

```
preferential_orientation_test <- function(circular_object)
```

Arguments

circular_object	A circular object containing the angles calculated between the bone long axis and each linear mark.

Details

The present function returns a list with the results for Rayleigh uniformity test and the associated p value, being a significant p value indicative of a non-uniform distribution.

See Also

calculate_orientations, rayleigh.test

Bibliography

Jammalamadaka, S. Rao and SenGupta, A. (2001). Topics in Circular Statistics, World Scientific Press, Singapore.

Example

```
data("right_femur")
data("femur_right_linear1")
data("femur_right_linear2")
example_circ_1 <- load_marks(femur_right_linear1, mark_type = "linear")
example_circ_2 <- load_marks(femur_right_linear2, mark_type = "linear")
example_calculate_orientations_1 <- calculate_orientations(example_circ_1, right_femur)
example_calculate_orientations_2 <- calculate_orientations(example_circ_2, right_femur)
example_pref_orientation1 <-preferential_orientation_test(example_calculate_orientations_1)
example_pref_orientation2 <- preferential_orientation_test(example_calculate_orientations_2)
```

compare_two_sample_orientations *Pair comparison for homogeneity*

Description

Function to compare the orientation patterns observed in two different samples of linear marks (scores and/or cut marks) using the randomised Mardia-Watson-Wheeler test.

Usage

```
compare_two_sample_orientations <- function (sample_1, sample_2)
```

Arguments

sample_1	A circular object containing the angles calculated between the bone long axis and each linear mark in the first dataset.
sample_2	A circular object containing the angles calculated between the bone long axis and each linear mark in the second dataset.

Details

The present function returns a list containing the results obtained for the Mardia-Watson-Wheeler test and the associated p value.

Author

Lloyd A. Courtenay

See Also

calculate_orientations, circular

Bibliography

Jammalamadaka, S. Rao and SenGupta, A. (2001). Topics in Circular Statistics, World Scientific Press, Singapore.

Example

```
data("right_femur")
data("femur_right_linear1")
data("femur_right_linear2")
example_circ_1 <- load_marks(femur_right_linear1, mark_type = "linear")
example_circ_2 <- load_marks(femur_right_linear2, mark_type = "linear")
example_calculate_orientations_1 <- calculate_orientations(example_circ_1, right_femur)
example_calculate_orientations_2 <- calculate_orientations(example_circ_2, right_femur)
example_comp_orientations <- compare_two_sample_orientations(
    example_calculate_orientations_1, example_calculate_orientations_2
)
```

create_time_series — *Creation of multiple time series plot.*

Description

The present function is used to generate a plot including the mark frequencies on several bone elements and to provide the basis for the addition of further samples.

Usage

```
create_time_series <- function(
    h = NULL, f = NULL, r = NULL, t = NULL, mt = NULL, mc = NULL,
    colour = "black",
    create_external_plot = FALSE
)
```

Arguments

h	A data frame containing the spatial coordinates representing the marks in the first humerus sample.
f	A data frame containing the spatial coordinates representing the marks in the first femur sample.
r	A data frame containing the spatial coordinates representing the marks in the first radius sample.
t	A data frame containing the spatial coordinates representing the marks in the first tibia sample.
mt	A data frame containing the spatial coordinates representing the marks in the first metatarsus sample.
mc	A data frame containing the spatial coordinates representing the marks in the first metacarpus sample.
colour	A lower case string specifying the colour of points and lines in the plot to represent the first set of samples.
create_external_plot	A boolean TRUE or FALSE (default = FALSE) option to create a popup window with the plot for mark frequency for all the samples.

Details

The present function returns a data frame and a plot including the sequence and mark frequencies of the loaded samples.

Author

Lloyd A. Courtenay

See Also

add_time_series, load_marks

Example

```
data("humerus_right_circular1")
data("femur_right_circular1")
data("radius_right_circular1")
data("tibia_right_circular1")
h1 <- load_marks(femur_right_circular1, mark_type = "circular", plot = FALSE)
f1 <- load_marks(femur_right_circular1, mark_type = "circular", plot = FALSE)
```

```
r1 <- load_marks(femur_right_circular1, mark_type = "circular", plot = FALSE)
t1 <- load_marks(femur_right_circular1, mark_type = "circular", plot = FALSE)
time_series_1 <- create_time_series(h = h1, f = f1, r = r1, t = t1)
```

add_time_series	*Add time series to a multiple time series plot.*

Description

The present function is used to add a time series plot to an already existing time series plot.

Usage

```
add_time_series <- function(h = NULL, f = NULL, r = NULL, t = NULL, mt =
    NULL, mc = NULL,
              colour = "red")
```

Arguments

h	A data frame containing the spatial coordinates representing the marks in an additional humerus sample.
f	A data frame containing the spatial coordinates representing the marks in an additional femur sample.
r	A data frame containing the spatial coordinates representing the marks in an additional radius sample.
t	A data frame containing the spatial coordinates representing the marks in an additional tibia sample.
mt	A data frame containing the spatial coordinates representing the marks in an additional metatarsus sample.
mc	A data frame containing the spatial coordinates representing the marks in an additional metacarpus sample.
colour	A lower case string specifying the colour of points and lines in the plot to represent the additional set of samples.

Details

The present function returns a data frame including the sequence and frequencies of all the additional samples. Mark frequencies are added to the plot previously created with the create_time_series function.

Author

Lloyd A. Courtenay

See Also

create_time_series, load_marks

Example

```r
data("humerus_right_circular1")
data("femur_right_circular1")
data("radius_right_circular1")
data("tibia_right_circular1")
h1 <- load_marks(femur_right_circular1, mark_type = "circular", plot = FALSE)
f1 <- load_marks(femur_right_circular1, mark_type = "circular", plot = FALSE)
r1 <- load_marks(femur_right_circular1, mark_type = "circular", plot = FALSE)
t1 <- load_marks(femur_right_circular1, mark_type = "circular", plot = FALSE)
time_series_1 <- add_time_series(h = h1, f = f1, r = r1, t = t1)

data("humerus_right_circular2")
```

```r
data("femur_right_circular2")
data("radius_right_circular2")
data("tibia_right_circular2")
h2 <- load_marks(femur_right_circular2, mark_type = "circular", plot = FALSE)
f2 <- load_marks(femur_right_circular2, mark_type = "circular", plot = FALSE)
r2 <- load_marks(femur_right_circular2, mark_type = "circular", plot = FALSE)
t2 <- load_marks(femur_right_circular2, mark_type = "circular", plot = FALSE)
time_series_2 <- add_time_series(h = h2, f = f2, r = r2, t = t2, colour = "red")

data("humerus_right_circular3")
data("femur_right_circular3")
data("radius_right_circular3")
data("tibia_right_circular3")
h3 <- load_marks(femur_right_circular1, mark_type = "circular", plot = FALSE)
f3 <- load_marks(femur_right_circular1, mark_type = "circular", plot = FALSE)
r3 <- load_marks(femur_right_circular1, mark_type = "circular", plot = FALSE)
t3 <- load_marks(femur_right_circular1, mark_type = "circular", plot = FALSE)
time_series_3 <- add_time_series(h = h3, f = f3, r = r3, t = t3, colour = "blue")
```

Example Code

```
# load library

library(IkhnosToolBox)

# load data

data("right_femur")
load_bone(right_femur)
data("femur_right_circular1")
data("femur_right_linear1")
data("femur_right_linear2")

example_c <- load_marks(
    femur_right_circular1,
    mark_type = "circular",
    plot = TRUE
)

example_l <- load_marks(
    femur_right_linear1,
    mark_type = "linear",
    plot = TRUE,
    colour_value = "red"
)

save_3d_image("femur_R_large_captive")

#
```

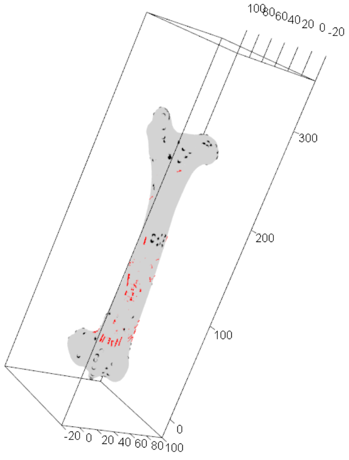

```
# BSM distribution:  CSR analyses

example_circular <- extract_spatial_data(example_1, mark_type = "circular")
example_linear <- extract_spatial_data(example_2, "linear")

perform_CSR_analyses(example_circular, n_permutations = 101)
perform_CSR_analyses(example_linear, n_permutations = 101)

#
```

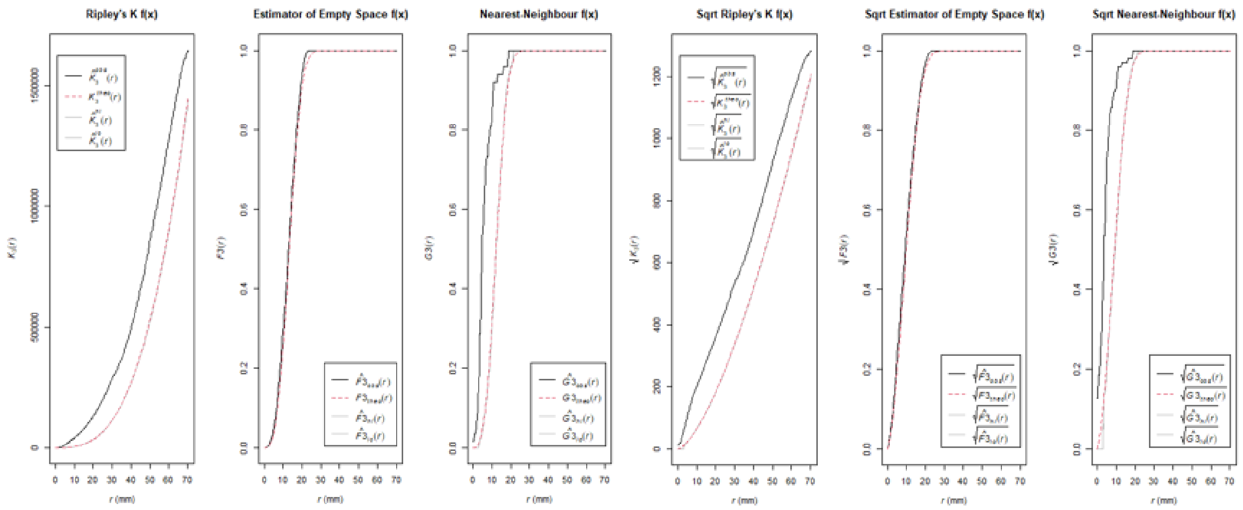
Ripley's K f(x)
Estimator of Empty Space f(x)
Nearest-Neighbour f(x)
Sqrt Ripley's K f(x)
Sqrt Estimator of Empty Space f(x)
Sqrt Nearest-Neighbour f(x)
r (mm)

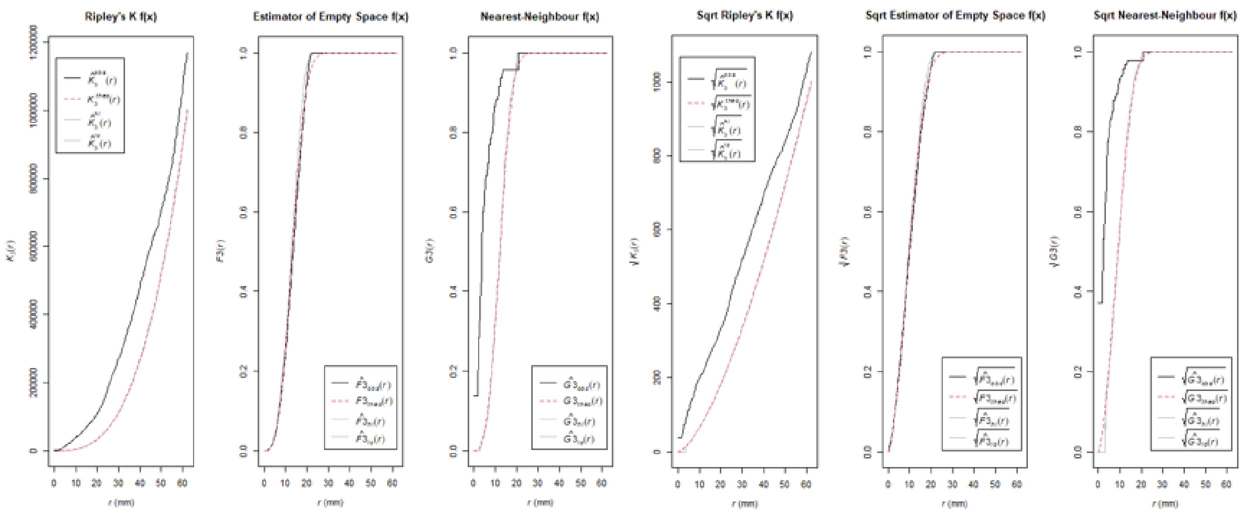
Ripley's K f(x)
Estimator of Empty Space f(x)
Nearest-Neighbour f(x)
Sqrt Ripley's K f(x)
Sqrt Estimator of Empty Space f(x)
Sqrt Nearest-Neighbour f(x)
r (mm)

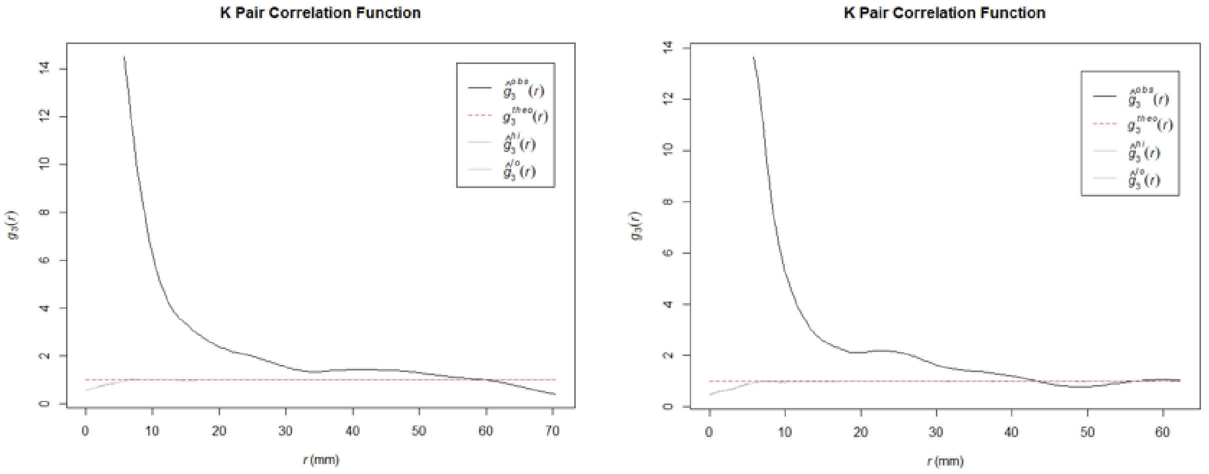
K Pair Correlation Function
K Pair Correlation Function
r (mm)
r (mm)

```
# BSM distribution:  tSNE

sample1_coords <- as.matrix(example_circular)
sample2_coords <- as.matrix(example_linear)
sample1_sample2 <- rbind(sample1_coords, sample2_coords)
group_labels <- as.factor(c(
  rep("circular", nrow(sample1_coords)),
  rep("linear", nrow(sample2_coords))
))
tsne_calculation(sample1_sample2, group_labels, bone = right_femur)

#
```

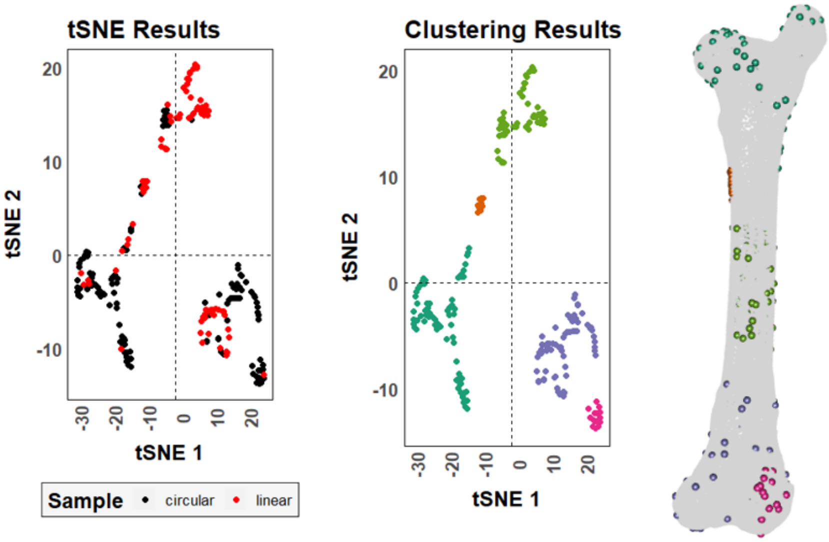


```
# BSM distribution:  Wavelet analysis

example_time_series <- two_sample_histogram_distributions(
  example_circular, example_linear,
  "circular", "linear",
  "x", "femur"
)

wavelet_analysis(
  example_time_series$first_sample_ts,
  example_time_series$second_sample_ts,
  "circular", "linear"
)

#
```

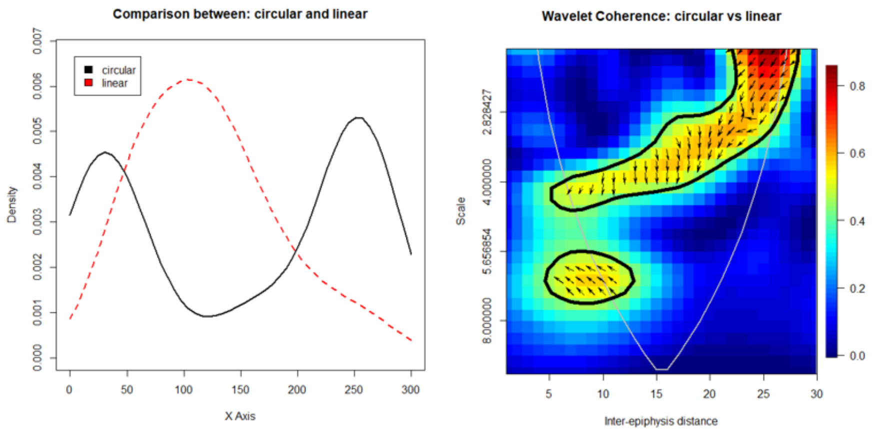


```
# Orientations

data("right_femur")

example_lin_1 <- load_marks(
  femur_right_linear1,
  mark_type = "linear",
  plot = FALSE
)

example_lin_2 <- load_marks(
  femur_right_linear2,
  mark_type = "linear",
  plot = FALSE
)

right_1 <- calculate_orientations(example_lin_1, right_femur)
```

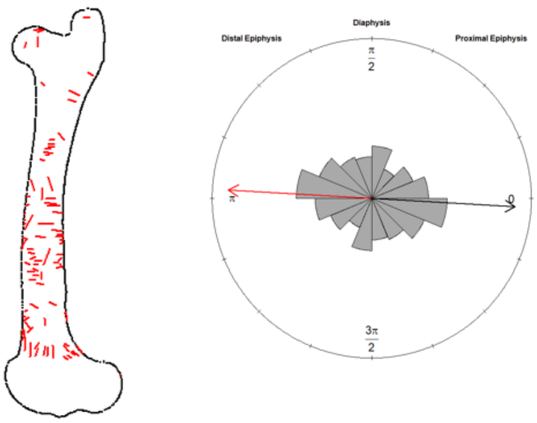


```
right_2 <- calculate_orientations(example_lin_2, right_femur)
```

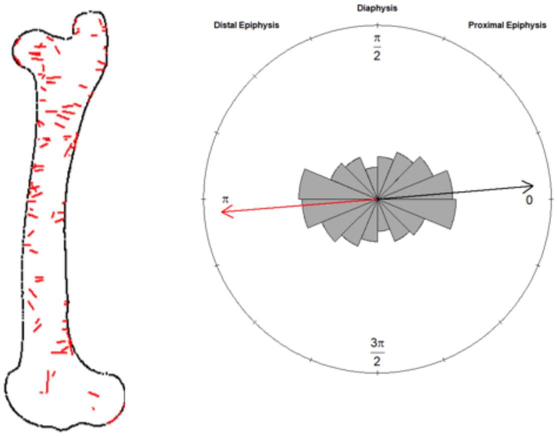


```
descriptive_circular_analysis(right_1)
```

```
$Standardised_Skewness
[1] -0.7537931

$Standardised_Kurtosis
[1] -0.1745812

$Sample_Circular_Variance
[1] 0.2792985

$Sample_Circular_Dispersion
[1] 0.693778

$Central_Orientation_Radians
[1] -0.05886981  3.08272284

$Central_Orientation_Degrees
[1]  -3.372992 176.627008
```

```
descriptive_circular_analysis(right_2)
```

```
$Standardised_Skewness
[1] -0.01957915

$Standardised_Kurtosis
[1] -0.3873598

$Sample_Circular_Variance
[1] 0.2094471

$Sample_Circular_Dispersion
[1] 0.5011373

$Central_Orientation_Radians
[1]  0.08365084 -3.05794181

$Central_Orientation_Degrees
[1]    4.79284 -175.20716
```

```
preferential_orientation_test(right_1)
```

```
$test_statistic
[1] 0.7207015

$p_value
[1] 2.3702e-20
```

```r
preferential_orientation_test(right_2)
```

```
$test_statistic
[1] 0.7905529

$p_value
[1] 1.34632e-27
```

```r
compare_two_sample_orientations(right_1, right_2)
```

```
$test_statistic
[1] 7.709033e-31

$p_value
[1] 1
```

```r

# Skeletal profile analysis

data("humerus_right_circular1")
data("humerus_right_circular2")
data("humerus_right_circular3")
data("femur_right_circular1")
data("femur_right_circular2")
data("femur_right_circular3")
data("tibia_right_circular1")
data("tibia_right_circular2")
data("tibia_right_circular3")
data("radius_right_circular1")
data("radius_right_circular2")
data("radius_right_circular3")
data("metatarsus_right_circular1")
data("metatarsus_right_circular2")
data("metatarsus_right_circular3")

# Series 1

h1 <- load_marks(
  humerus_right_circular1,
  mark_type = "circular", plot = FALSE
)
f1 <- load_marks(
  femur_right_circular1,
  mark_type = "circular", plot = FALSE
)
r1 <- load_marks(
  radius_right_circular1,
  mark_type = "circular", plot = FALSE
)
t1 <- load_marks(
  tibia_right_circular1,
  mark_type = "circular", plot = FALSE
)
mt1 <- load_marks(
  metatarsus_right_circular1,
  mark_type = "circular", plot = FALSE
)

# Series 2

h2 <- load_marks(
  humerus_right_circular2,
  mark_type = "circular", plot = FALSE
)
f2 <- load_marks(
  femur_right_circular2,
  mark_type = "circular", plot = FALSE
)
r2 <- load_marks(
```

```r
  radius_right_circular2,
  mark_type = "circular", plot = FALSE
)
t2 <- load_marks(
  tibia_right_circular2,
  mark_type = "circular", plot = FALSE
)
mt2 <- load_marks(
  metatarsus_right_circular2,
  mark_type = "circular", plot = FALSE
)

# Series 3

h3 <- load_marks(
  humerus_right_circular3,
  mark_type = "circular", plot = FALSE
)
f3 <- load_marks(
  femur_right_circular3,
  mark_type = "circular", plot = FALSE
)
r3 <- load_marks(
  radius_right_circular3,
  mark_type = "circular", plot = FALSE
)
t3 <- load_marks(
  tibia_right_circular3,
  mark_type = "circular", plot = FALSE
)
mt3 <- load_marks(
  metatarsus_right_circular3,
  mark_type = "circular", plot = FALSE
)


time_series_1 <- create_time_series(h = h1, f = f1, r = r1, t = t1, mt = mt1, create_external_plot =
    TRUE)
time_series_2 <- add_time_series(h = h2, f = f2, r = r2, t = t2, mt = mt2, colour = "red")
time_series_3 <- add_time_series(h = h3, f = f3, r = r3, t = t3, mt = mt3, colour = "blue")

#
```

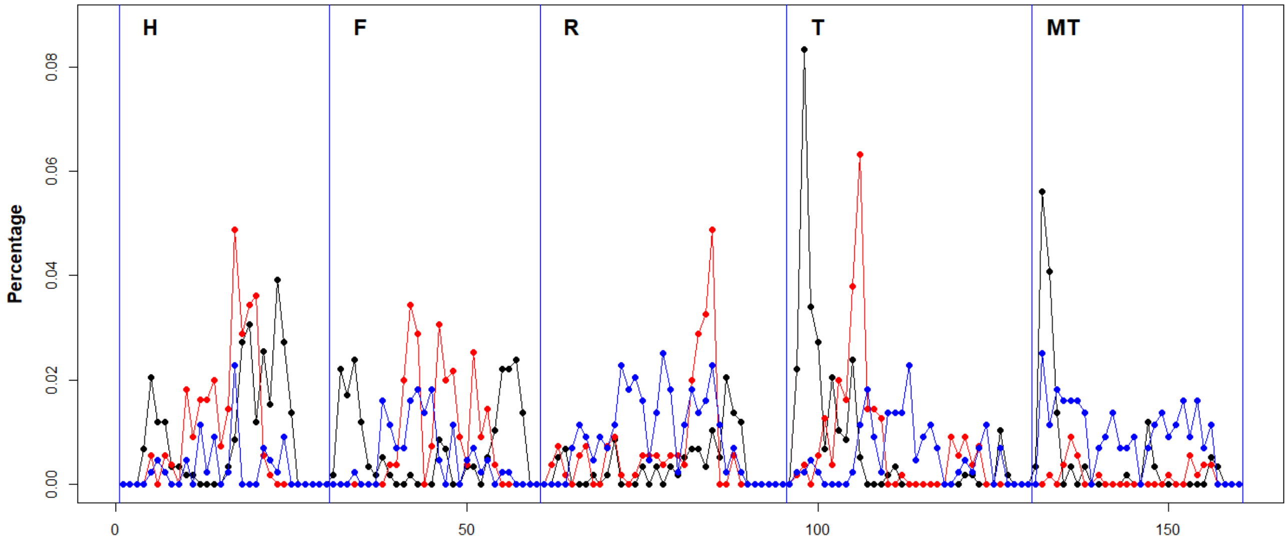

Supplement: Supplementary file 1 [file animals-12-02861-s001.zip › Animals_S2.pdf]
