# Supplementary material for: Ikhnos: A Novel Software to Register and Analyze Bone Surface Modifications Based on Three-Dimensional Documentation
Source: Animals (Basel). 2022 Oct 20;12(20):2861. doi: 10.3390/ani12202861 (PMC9598086; doi:10.3390/ani12202861)
Supplement: Supplementary file 1 [file animals-12-02861-s001.zip › Animals_S3.pdf]

# *Ikhnos*: a novel software to register and analyse bone surface modifications based on three-dimensional documentation

# Supplementary File S3: Case Study

Rocío Mora [1], Julia Aramendi [1,2], Lloyd A. Courtenay [1,3], Diego González-Aguilera [1*], José Yravedra [3,4], Miguel Ángel Maté-González [5,1], Diego Prieto-Herráez [6,1], José Mª Vázquez-Rodríguez [7], Isabel Barja [8,9]

[1] Department of Cartographic and Land Engineering, Higher Polytechnic School of Avila, Universidad de Salamanca, Hornos Caleros 50, 05003, Ávila, Spain

[2] Department of Geology, Facultad de Ciencia y Tecnología, Universidad del País Vasco - Euskal Herriko Unibertsitatea (UPV/EHU), Barrio Sarriena S/n, 48940 Leioa, Spain

[3] Department of Prehistory, Ancient History and Archaeology, Universidad Complutense de Madrid, Prof. Aranguren 8 s/n, 28040, Madrid, Spain

[4] C. A. I. Archaeometry and Archaeological Analysis, Universidad Complutense de Madrid, 28040, Madrid, Spain

[5] Department of Topographic and Cartography Engineering, Higher Technical School of Engineers in Topography, Geodesy and Cartography, Universidad Politécnica de Madrid, Mercator 2,28031 Madrid, Spain

[6] Institute of Fundamental Physics and Mathematics, Merced Building, Universidad de Salamanca, Plaza de la Merced 1, 37008, Salamanca, Spain

[7] Department of Prehistory and Archaeology, Humanities Faculty, UNED, C/Senda del Rey, 7, 28040 Madrid, Spain

[8] Zoology Unit, Department of Biology, Universidad Autónoma de Madrid, C/Darwin 2, Campus Universitario de Cantoblanco, 28049 Madrid, Spain

[9] Center of Investigation in Biodiversity and Global Change (CIBC-UAM), Universidad Autónoma de Madrid, 28049 Madrid, Spain

*Correspondence: daguilera@usal.es

## Materials

The tooth mark samples originated from wild wolf packs from two areas: Villardeciervos (Zamora, Spain) and Pico Vízcares (Asturias, Spain). The red deer (*C. elaphus*) sample from Villardeciervos was collected between the months of May and September of 2010 by one of the authors (J.Y.), who was aided by forest rangers where necessary. The sample from Pico Vízcares was collected in August of 2020 in an area for livestock grazing located 1.3km South from Pico Vízcares (1421mts above sea level). The bones belong to a single carcass that could not be fully recovered despite intensive search in the surrounding area by J.M.V.R. Control of whether only wolves had intervened in samples was based on the current knowledge about the ecology of both areas.

Tooth mark samples from captive wolves were obtained from El Hosquillo (Cuenca, Castilla-La Mancha). Samples were collected during the winter months of 2010 and 2011. See previous works [29,58,59] for further details on the sample. The sample was produced by five adult individuals. Animals were fed disarticulated limb elements with meat attached. Bones were exposed to animals for three months. The wolf enclosure in El Hosquillo is 10,000 $m^2$, approximately 0.1% of the 910 ha of the entire natural park. El Hosquillo, is accessible to visitors (14,426 in 2015 and 20,000 in 2018) by motor vehicles, but it provides a sensible distance between the asphalt tracks and each enclosure. The park is only open to the public on weekends or festive days, with week days generally being reserved for scholars, thus ensuring less exposure of the wolves to the public. All experiments involving carnivores were performed in accordance with the relevant guidelines as set forth by park keepers and general park regulations. Collection of chewed bones was performed directly by park staff, assisted by one of the co-authors (J.Y.). Bone samples were provided directly by the park in accordance with their standardised feeding protocols.

No animals were sacrificed specifically for the purpose of these experiments. Carnivores were not manipulated or handled at any point throughout the collection of samples. No licenses or permits were required to perform these experiments. Once collected, all bone samples were cleaned in boiling water without the use of additional chemical agents.

# Results

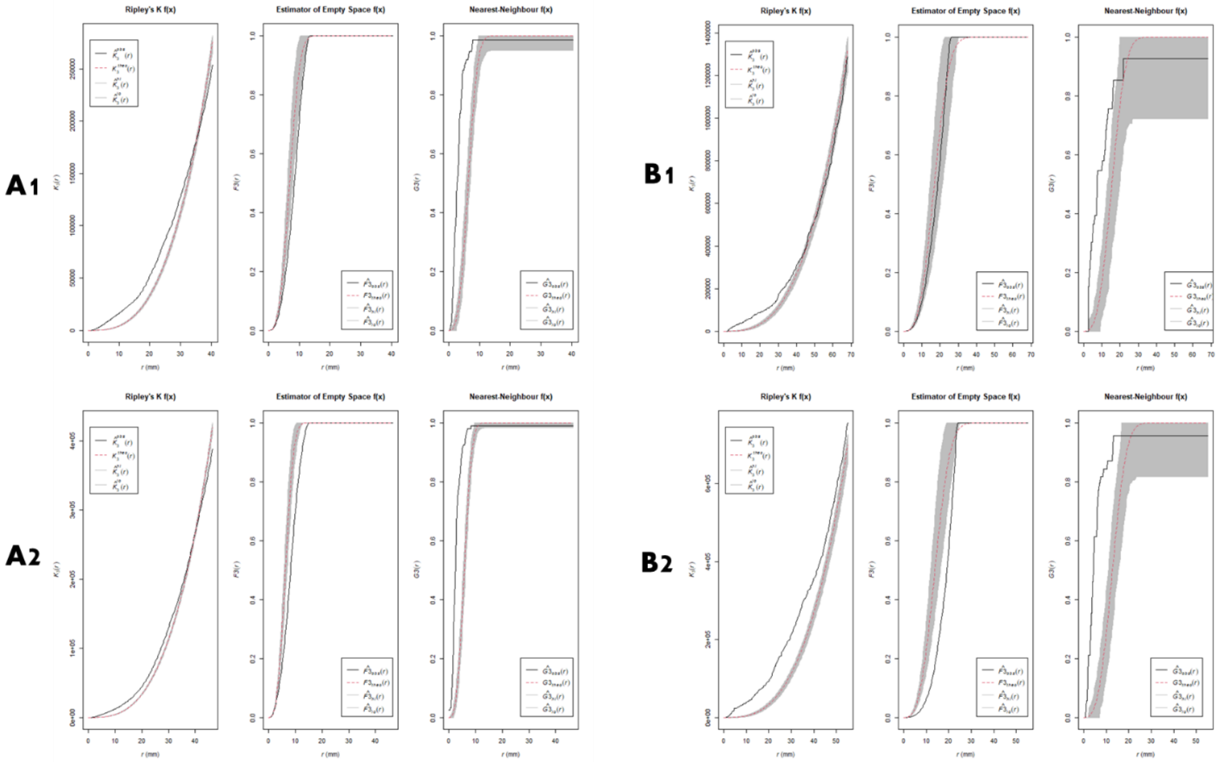


**Supplementary Figure S1:** Plots of the *K*-function, *F* empty space function and *G* near-neighbour function for A1) tooth marks on right femora generated by captive wolves; A2) tooth marks on left femora generated by captive wolves; B1) tooth marks on right femora generated by wild wolves; B2) tooth marks on left femora generated by wild wolves; Dotted red line shows the Poisson Complete Spatial Random (CSR) process, the black line shows the empirical point process of the target sample, and the grey band shows simulated confidence intervals

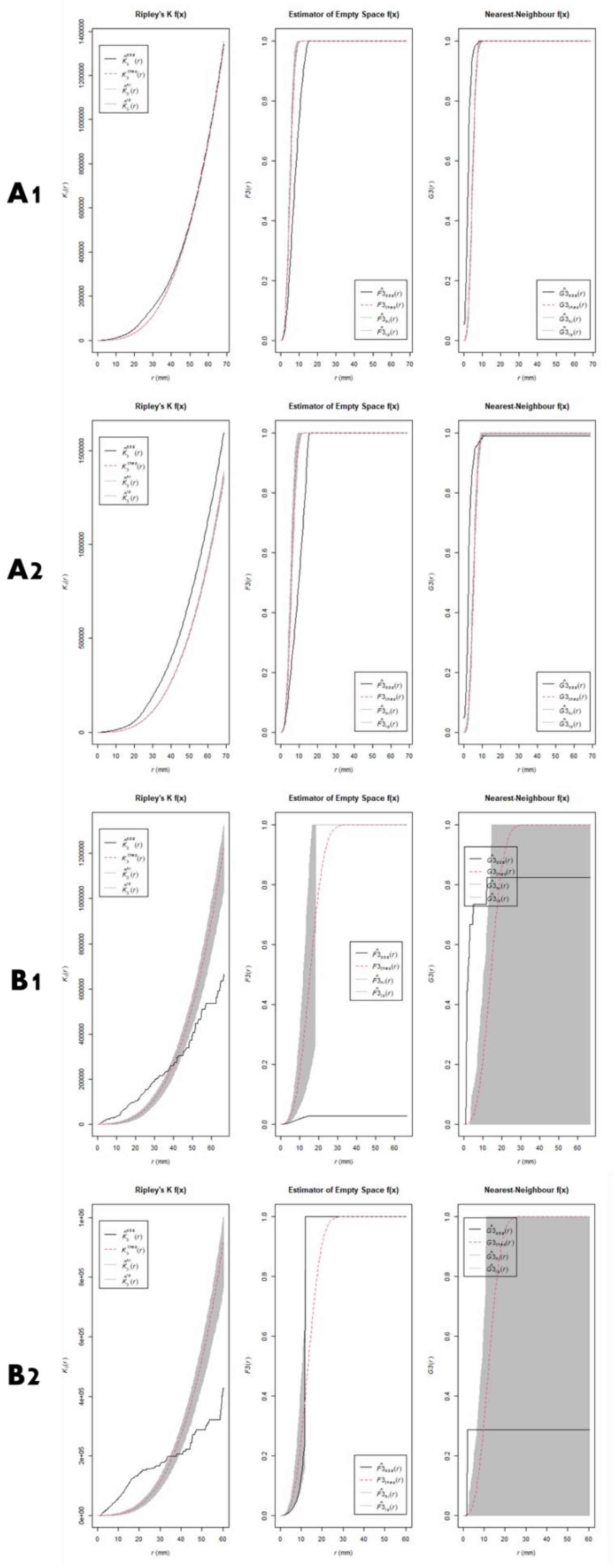


**Supplementary Figure S2:** Plots of the *K*-function, *F* empty space function and *G* near-neighbour function for A1) tooth marks on right tibiae generated by captive wolves; A2) tooth marks on left tibiae generated by captive wolves; B1) tooth marks on right tibiae generated by wild wolves; B2) tooth marks on left tibiae generated by wild wolves. Dotted red line shows the Poisson Complete Spatial Random (CSR) process, the black line shows the empirical point process of the target sample, and the grey band shows simulated confidence intervals.

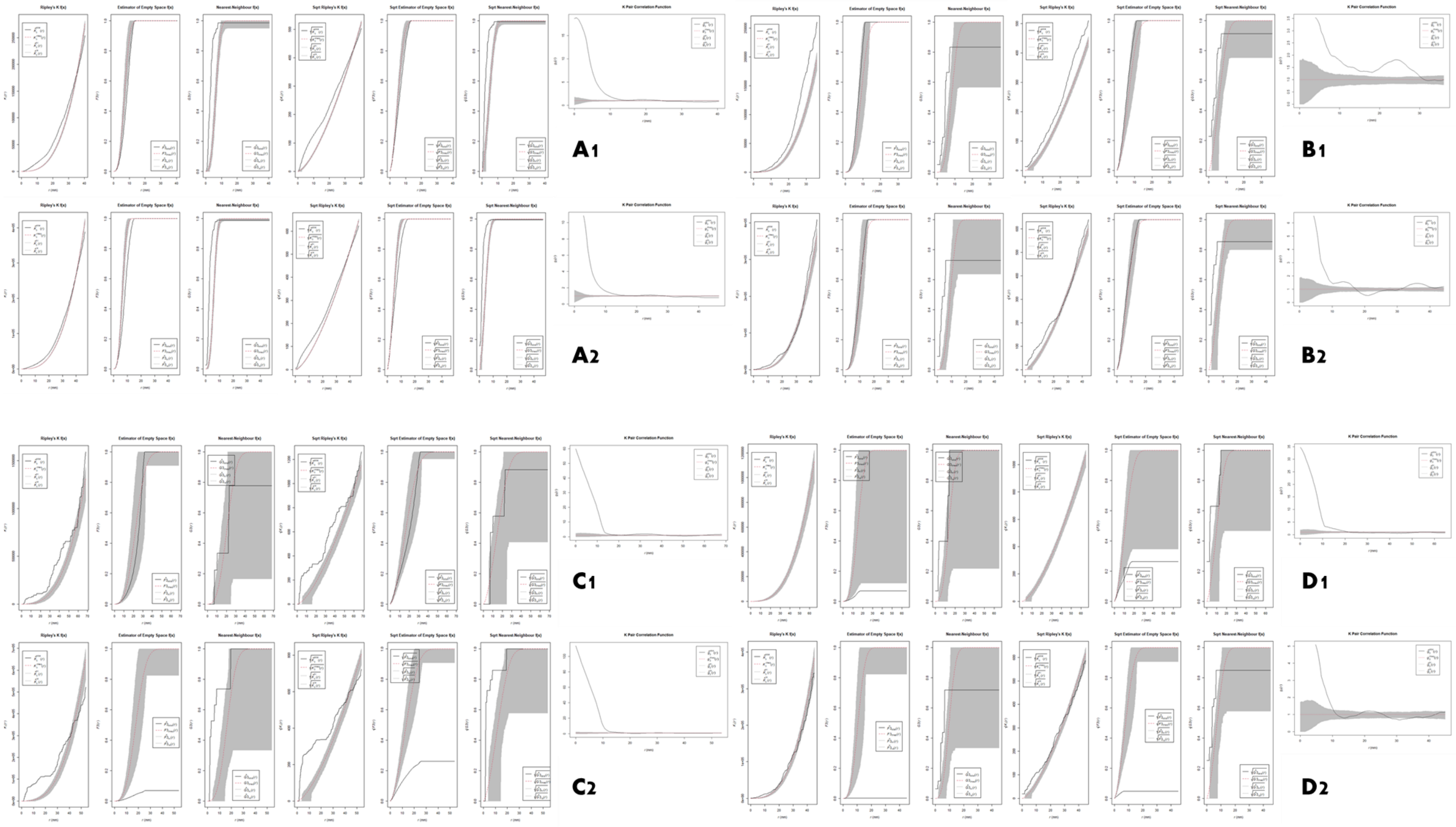


**Supplementary Figure S3:** Plots of the K-function, F empty space function, G near-neighbour function and Pair-correlation function, and the square root version of the first three ones for A1) pits on right femora generated by captive wolves; A2) pits on left femora generated by captive wolves; B1) scores on right femora generated by captive wolves; B2) scores on left femora generated by captive wolves; C1) pits on right femora generated by wild wolves; C2) pits on left femora generated by wild wolves; D1) ) scores on right femora generated by wild wolves; D2) scores on left femora generated by wild wolves. Dotted red line shows the Poisson Complete Spatial Random (CSR) process, the black line shows the point process of the target sample and the grey band shows its confidence envelope. For the K and G functions, a distribution above the Poisson process region indicates clustering, whereas for the F function clustering is highlighted by distribution below the Poison process

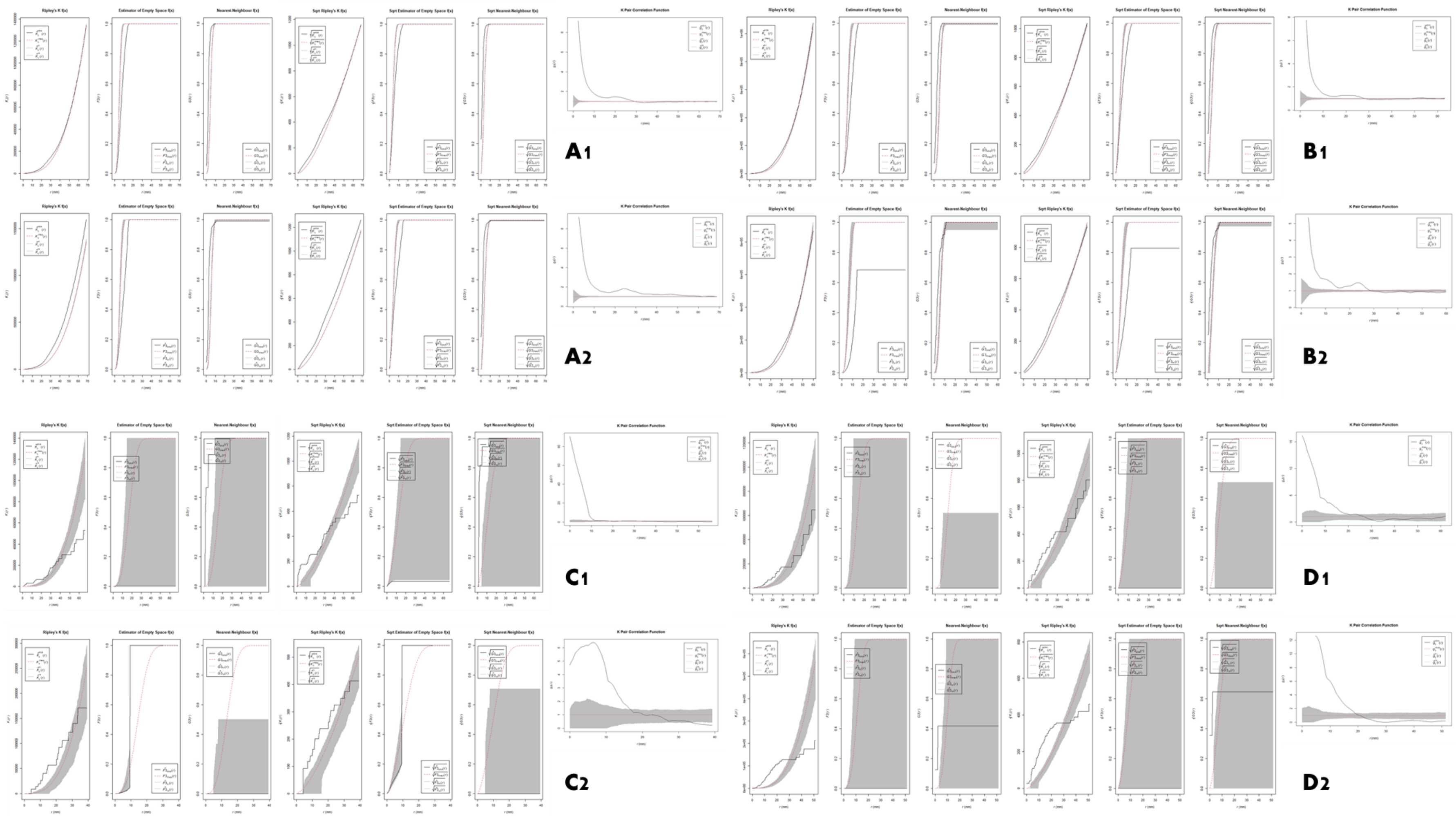


**Supplementary Figure S4:** Plots of the K-function, F empty space function, G near-neighbour function and Pair-correlation function, and the square root version of the first three ones for A1) pits on right tibiae generated by captive wolves; A2) pits on left tibiae generated by captive wolves; B1) scores on right tibiae generated by captive wolves; B2) scores on left tibiae generated by captive wolves; C1) pits on right tibiae generated by wild wolves; C2) pits on left tibiae generated by wild wolves; D1) ) scores on right tibiae generated by wild wolves; D2) scores on left tibiae generated by wild wolves. Dotted red line shows the Poisson Complete Spatial Random (CSR) process, the black line shows the point process of the target sample and the grey band shows its confidence envelope. For the K and G functions, a distribution above the Poisson process region indicates clustering, whereas for the F function clustering is highlighted by distribution below the Poison process

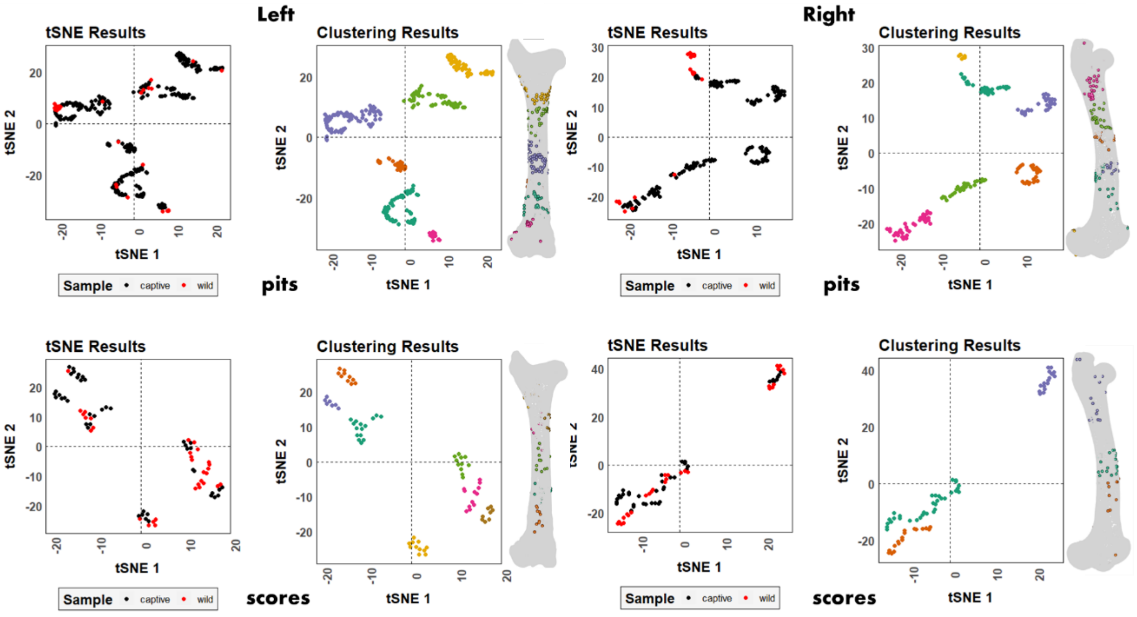


**Supplementary Figure S5:** t-SNE scatter plots for tooth pits and scores on the femora modified by captive and wild wolves

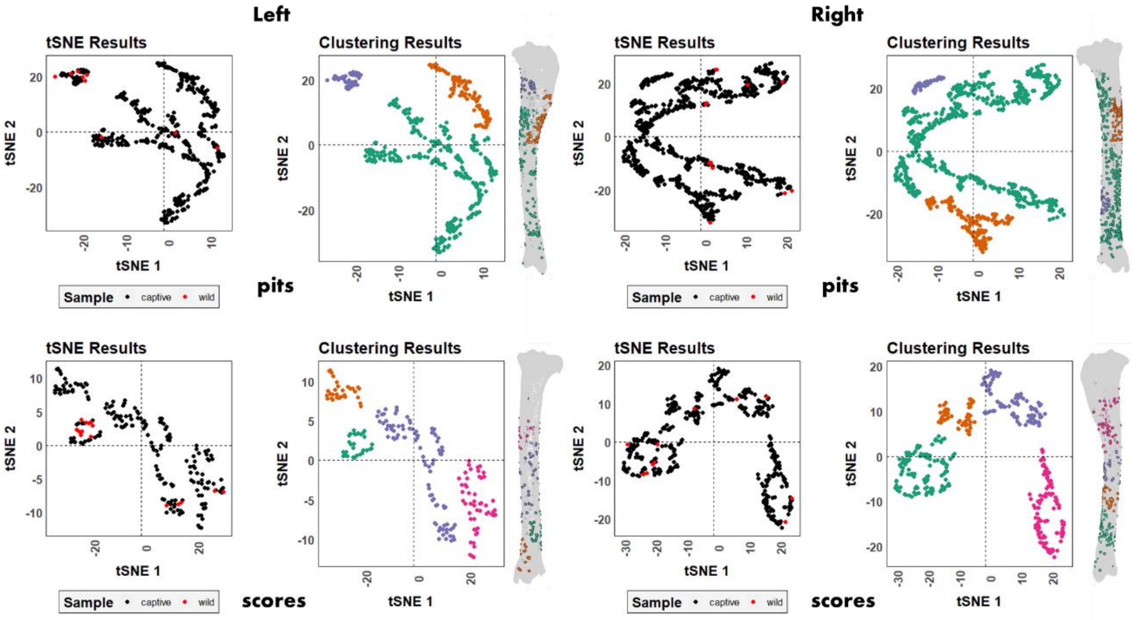


**Supplementary Figure S6:** t-SNE scatter plots for tooth pits and scores on the tibiae modified by captive and wild wolves

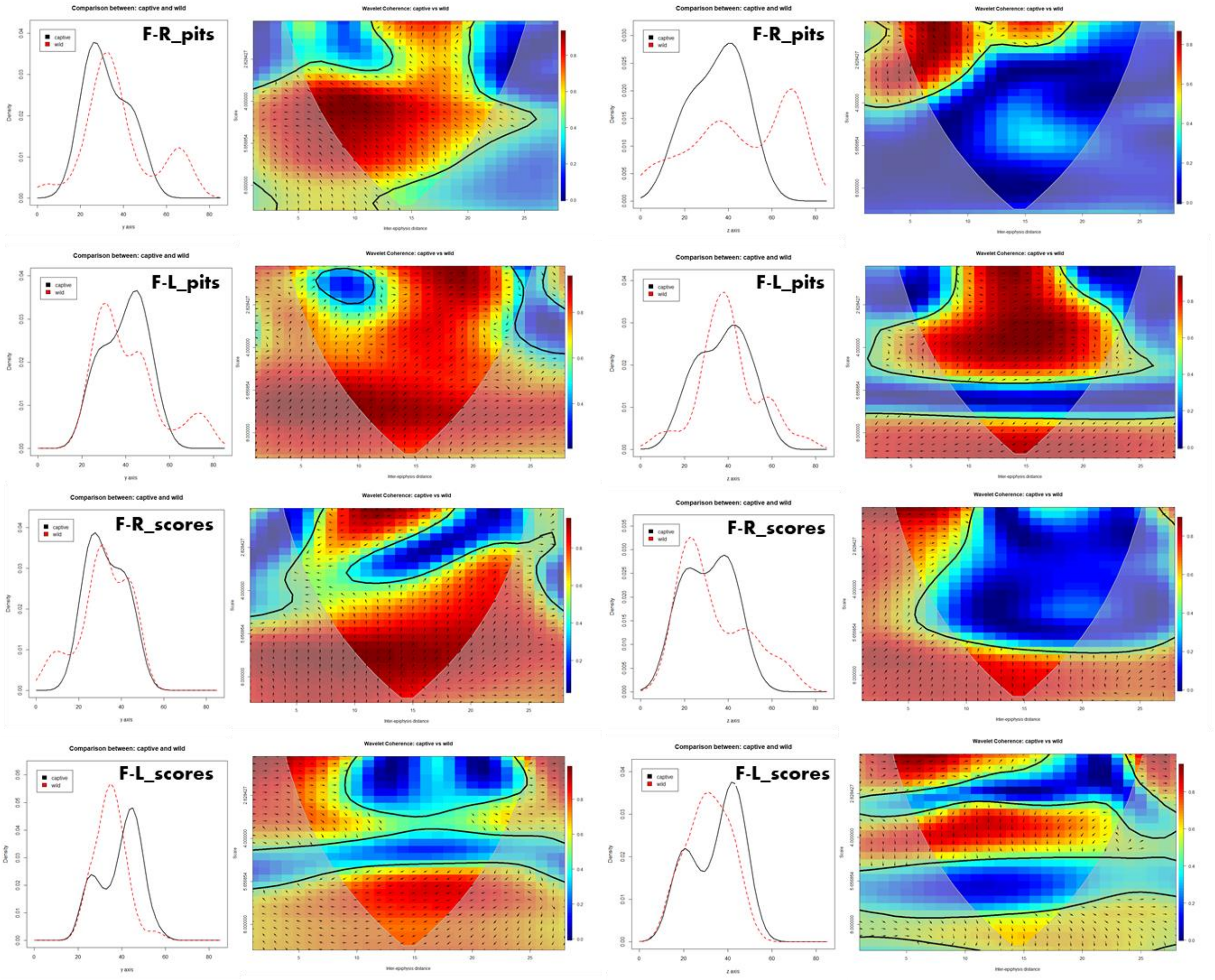


**Supplementary Figure S7:** Pair histograms of tooth pit and score occurrence along the y and z bone axes and bivariate wavelet coherence plots showing the correlation of tooth modification produced by wild and captive wolves on the medio-lateral axis (left) and the cranio-caudal axis (right) of the analysed femora. Correlation between both datasets is colour coded, with correlation represented in red and no dependence indicated in blue. Arrows represent the lead/lag phase relations between the datasets. Arrows pointing to the right (→) means that the time series are in phase, that is they covary in the same direction. Arrows pointing to the left (←) means that the time series are in anti-phase, that is the samples change in opposite directions. Arrows pointing to the right-down or left-up indicate that the first variable (captive) is leading, while arrows pointing to the right-up or left-down indicate that the second variable (wild) is leading

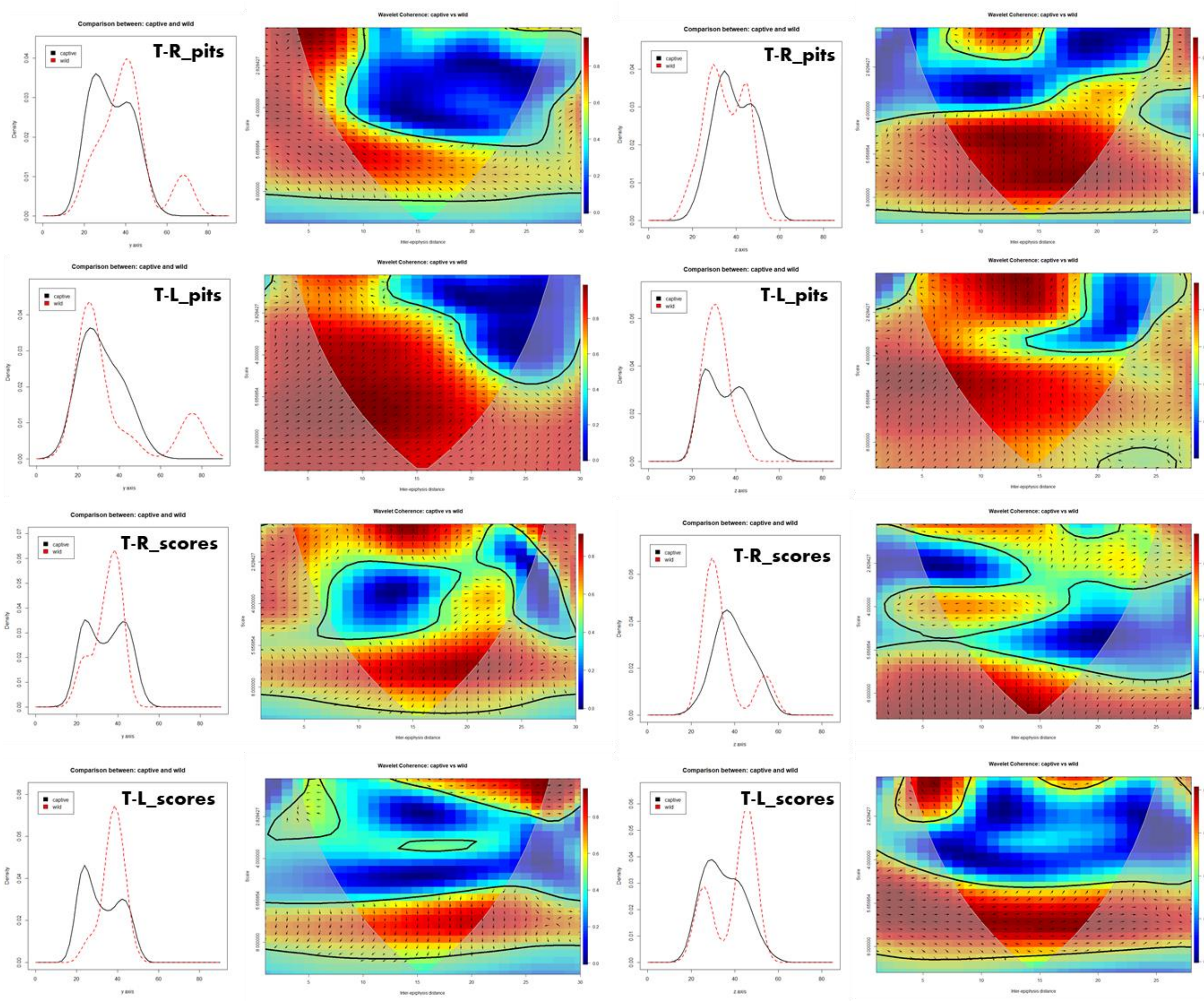


**Supplementary Figure S8:** Pair histograms of tooth pit and score occurrence along the y and z bone axes and bivariate wavelet coherence plots showing the correlation of tooth modification produced by wild and captive wolves on the medio-lateral axis (left) and the cranio-caudal axis (right) of the analysed tibiae. Correlation between both datasets is colour coded, with correlation represented in red and no dependence indicated in blue. Arrows represent the lead/lag phase relations between the datasets. Arrows pointing to the right (→) means that the time series are in phase, that is they covary in the same direction. Arrows pointing to the left (←) means that the time series are in anti-phase, that is the samples change in opposite directions. Arrows pointing to the right-down or left-up indicate that the first variable (captive) is leading, while arrows pointing to the right-up or left-down indicate that the second variable (wild) is leading

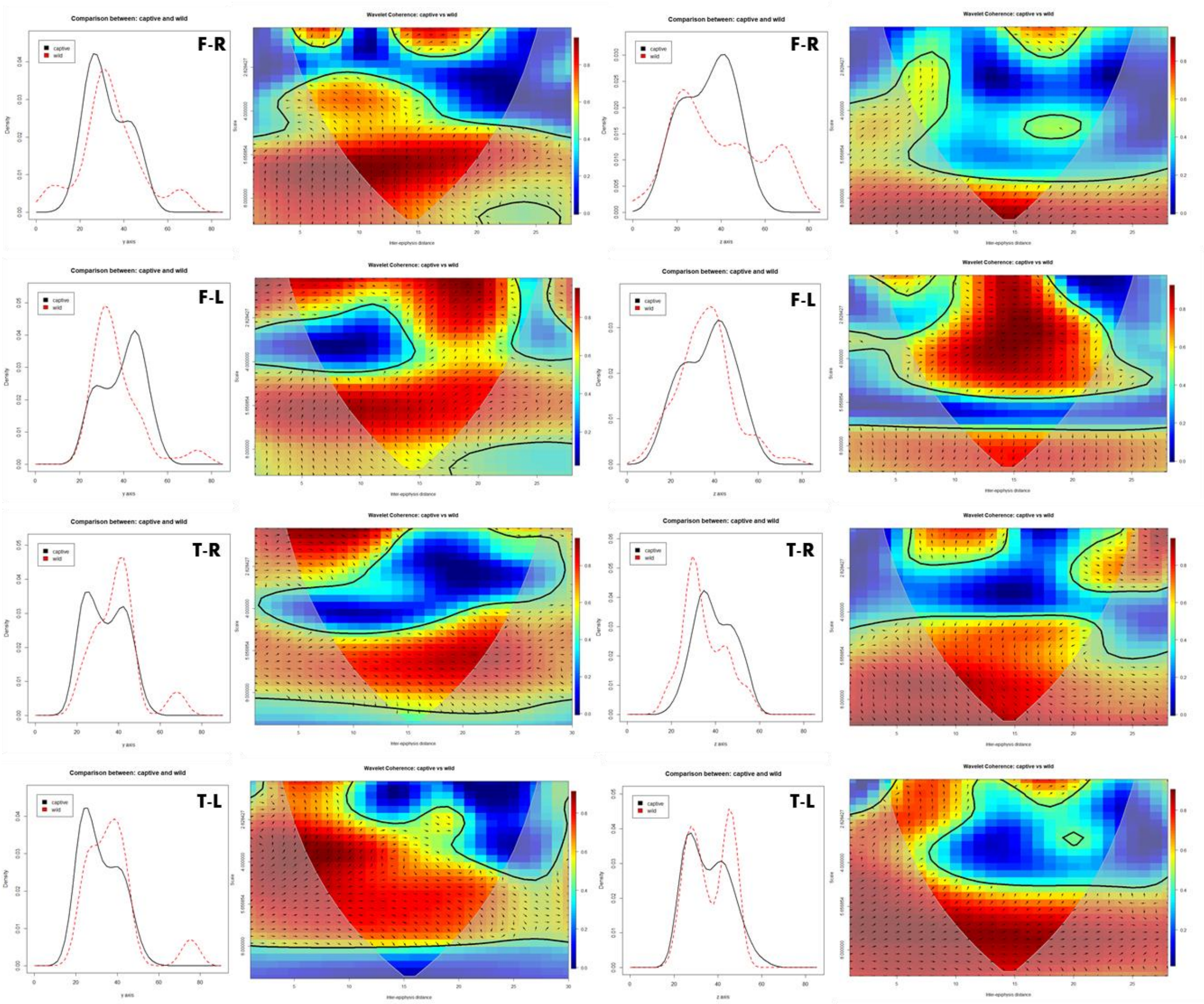


**Supplementary Figure S9:** Pair histograms of tooth mark occurrence along the y and z bone axes and bivariate wavelet coherence plots showing the correlation of tooth modification (including both pits and scores) produced by wild and captive wolves on the medio-lateral axis (left) and the cranio-caudal axis (right) of the analysed femora and tibiae. Correlation between both datasets is colour coded, with correlation represented in red and no dependence indicated in blue. Arrows represent the lead/lag phase relations between the datasets. Arrows pointing to the right (→) means that the time series are in phase, that is they covary in the same direction. Arrows pointing to the left (←) means that the time series are in anti-phase, that is the samples change in opposite directions. Arrows pointing to the right-down or left-up indicate that the first variable (captive) is leading, while arrows pointing to the right-up or left-down indicate that the second variable (wild) is leading

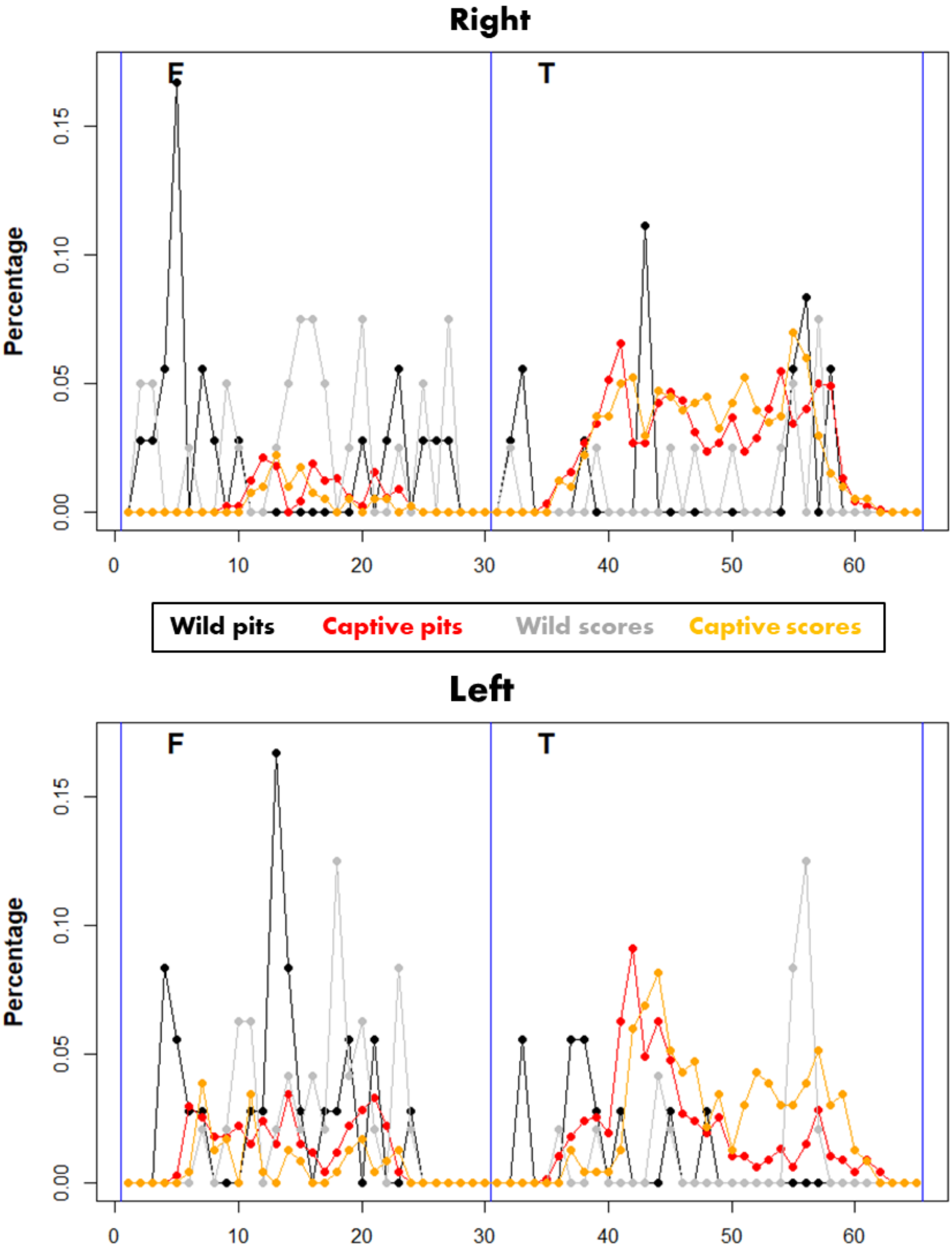


**Supplementary Figure S10:** Averaged relative distribution of tooth scores, including pits and scores, in the wild and captive wolf samples. The X axis shows the longitudinal dimensions of the series of the femur and the tibia placed sequentially

# References


29. Courtenay, L.A.; Herranz-Rodrigo, D.; González-Aguilera, D.; Yravedra, J. Developments in Data Science Solutions for Carnivore Tooth Pit Classification. *Scientific Reports* **2021**, *11*, 10209.
58. Courtenay, L.A.; Yravedra, J.; Maté-González, M.A.; Vázquez-Rodríguez, J.M.; Fernández-Fernández, M.; González-Aguilera, D. The effects of prey size on carnivore tooth mark morphologies on bone; the case study of Canis lupus signatus. *Historical Biology* **2020**, *33*, 2760-2772.
59. Courtenay, L.A.; Herranz-Rodrigo, D.; Yravedra, J.; Vázquez-Rodríguez, J.M.; Huguet, R.; Barja, I.; Maté-González, M.A.; Fernández-Fernández, M.; Muñoz-Nieto, A.L.; González-Aguilera, D. 3D Insights into the Effects of Captivity on Wolf Mastication and Their Tooth Marks; Implications in Ecological Studies of Both the Past and Present. *Animals* **2021**, *11*, 2323.
